# Supplementary material for: Preserving precise choreography of bonds in Z-stereoretentive olefin metathesis by using quinoxaline-2,3-dithiolate ligand
Source: Nat Commun. 2024 Oct 17;15:8981. doi: 10.1038/s41467-024-52876-4 (PMC11487267; doi:10.1038/s41467-024-52876-4)
Supplement: Supplementary file 1 — Supplementary Information [file 41467_2024_52876_MOESM1_ESM.pdf]

Supplementary Information

Preserving Precise Choreography of Bonds in Z-Stereoretentive Olefin Metathesis by Using Quinoxaline-2,3-dithiolate Ligand

Łukasz Grzesiński,[1] Maryana Nadirova,[1] Jannick Guschlbauer,[1] Artur Brotons-Rufes,[2] Albert Poater,[2,*] Anna Kajetanowicz,[1,*] and Karol Grela[1,*]

[1] Biological and Chemical Research Centre, Faculty of Chemistry, University of Warsaw, Żwirki i Wigury 101, 02-089 Warsaw, Poland, a.kajetanowicz@uw.edu.pl, klgre@uw.edu.pl
[2] Institut de Química Computacional i Catàlisi and Departament de Química, Universitat de Girona, c/ Mª Aurèlia Capmany 69, 17003 Girona, Catalonia, Spain, albert.poater@udg.edu

1. Supplementary Methods 4
1.1. General Remarks 4
2. Supplementary discussion 6
2.1. Synthesis of Ruthenium complexes 6
2.1.1. Synthesis of 3,6-dichloro-1,2-dithiobenzeneethylenodiamine zinc complex (46)6
2.1.2. Synthesis of Ru2 6
2.1.3. Synthesis of 1,4-dihydroquinoxaline-2,3-dione (4) 7
2.1.4. Synthesis of 2,3-dichloroquinoxaline (6) 8
2.1.5. Synthesis of 2,3-dithoqunoxaline (10) 8
2.1.6. Synthesis of dithioquinoxaline-diamine zinc complex (8) 9
2.1.7. Synthesis of Ru3 9
2.2. Catalyst stability 10
2.2.1. Stability at solid state in air at room temperature 10
2.2.2. Stability at 60 °C 11
2.2.3. Stability at 110 °C 13
2.3. General procedure for mRCM in reactive distillation conditions 15
2.3.1. Synthesis of (*Z*)-oxacyclohexadec-10-en-2-one (2) 16
2.3.2. Synthesis of (*Z*)-oxacyclotridec-10-en-2-one (11, Yuzu Lactone) 16
2.3.3. Synthesis of (*Z*)-oxacyclononadec-10-ene (12) 17
2.3.4. Synthesis of (*Z*)-cycloheptadec-9-en-1-one (13, Civetone) 18
2.4. Self-metathesis “dimerisation” reactions 19
2.4.1. Synthesis of (*Z*)-hex-3-ene-1,6-diol (16) 19
2.4.2. Synthesis of (*Z*)-dodec-6-ene-1,6-diol (18) 19
2.4.3. Synthesis of (*Z*)-5-hydroxypent-2-en-1-yl acetate (20) – self-CM reaction and subsequent CM reaction 20
2.4.4. Synthesis of (*Z*)-dodec-6-enedial (22) 20
2.5. Cross-Metathesis Reactions 21
2.5.1. Solvents scope 21
2.5.2. General procedure for cross-metathesis reactions 22
2.5.3. (*Z*)-4-(4-methoxyphenyl)but-2-en-1-yl acetate (24) 22
2.5.4. Synthesis of (*Z*)-7-hydroxyhept-2-en-1-yl acetate (25) 22
2.5.5. Synthesis of (*Z*)-4-phenylbut-2-en-1-yl benzoate (26) 23
2.5.6. Synthesis (*Z*)-9-hydroxynon-7-enal (27) 23
2.6. Synthesis of (*Z*)-tridec-2-en-1-ol 24
2.7. Cross-Metathesis Reactions of API Derivatives 24
2.7.1. Synthesis of estrone derivative (29) 25
2.7.2. (*Z*)-4-((2R,3S)-3-((S)-1-((tert-butyldimethylsilyl)oxy)ethyl)-4-oxoazetidine-2-yl)but-2-en-1-yl acetate (30) 25
2.7.3. Synthesis of (*Z*)-(1-(14-hydroxytetradec-8-en-1-yl)-1*H*-indol-3-yl) (2,2,3,3-tetramethylcyclopropyl)methanone (31) 26
2.7.4. Synthesis of Sildenafil derivative (32) 27
2.7.5. Synthesis of (*Z*)-9-hydroxynon-3-en-1-yl 3-(4-(7*H*-pyrrolo[2,3-d]pyrimidin-4-yl)-1*H*-pyrazol-1-yl)-1-(ethylsulfonyl)azetidine-3-carboxylate (33) 29
2.8. Pheromones or pheromone precursors obtained in the CM reaction 31
2.8.1. Synthesis of methyl (*Z*)-tetradec-9-enoate (40) 31
2.8.2. Synthesis of (Z)-octadec-9-en-1-yl acetate (41) 31
2.8.3. Synthesis of (*Z*)-dodec-9-en-1-yl acetate (42) 32
2.8.4. Synthesis of oct-7-en-1-yl acetate 32

2.8.5. Synthesis of (7*E*,9*Z*)-dodeca-7,9-dien-1-yl acetate (44) 33
2.8.6. Synthesis of (7*E*,9*Z*)-dodeca-7,9-dien-1-yl acetate (44) using the Wittig reaction (as a product standard for preparation of the calibration curve) 33
2.8.7. Synthesis of (7*E*,9*Z*)-dodeca-7,9-dien-1-yl acetate (44) in CM reaction 33
2.9. Reproduction of NMR spectra 35
2.9.1. Compound for catalysts synthesis 35
2.9.2. Products of metathesis reactions 43
2.10. X-Ray 59
2.11. Computational Study 60
2.11.1. Computational Details 60
2.11.2. Computational Results 61
3. Supplementary References 72

1. Supplementary Methods

1.1. General Remarks

All reactions requiring the exclusion of oxygen and moisture were performed in dry glassware with dry solvents (SPS MBraun) under a moisture- and oxygen-free argon atmosphere using standard Schlenk technique. The addition of dry solvents or reagents was carried out using argon flushed stainless steel cannulas and plastic syringes. For high-concentration ring-closing metathesis reactions (HC-RCM) the following vacuum pumps were used (as noted in descriptions of reaction procedures):

- Rotary Vane Pump (RVP): Vacuumbrand RZ 2.5 (maximum nominal vacuum of 1×10^{-3} mBar).
- Oil Diffusion Pump (ODP): Vacuumbrand High-Vacuum Pumping Unit HP 40 B2/RZ 6 (maximum nominal vacuum of 1×10^{-6} mBar).

For spectroscopic and analytic characterisations, the following devices were used:

Analytical thin layer chromatography (TLC) was performed on Merck Silica gel 60 F254 pre-coated aluminium sheets. Components were visualised by observation under UV light (254 nm or 365 nm) or dyed with aqueous $KMnO_4$ or anisaldehyde reagent.

Flash column chromatography was carried out using silica gel 60 (230 – 400 mesh), purchased from Merck.

GC chromatograms were recorded using a PerkinElmer Clarus 580 model. As a capillary column, an IntertCap 5MS-Sil column was used with helium as carrier gas. GC conversions were determined based on the ratio of an internal standard (durene or tetradecane) and the starting material.

^{1}H NMR spectra were recorded in dichloromethane-d_2, chloroform-d_1, or DMSO-d_6 at room temperature on Agilent Mercury spectrometers (400 MHz). The data were interpreted in first order spectra. Chemical shifts δ are reported in parts per million (ppm) downfield from trimethylsilane as reference to residual solvent signal: dichloromethane-d_2 [δH = 5.32 ppm], chloroform-d_1 [δH = 7.26 ppm], DMSO-d_6 [δH = 2.50 ppm]. The following abbreviations are used to indicate the signal multiplicity: s (singlet), d (doublet), t (triplet), q (quartet), quin (quintet), sext (sextet), dd (doublet of doublet), dt (doublet of triplet), ddd (doublet of doublet of doublet), etc., br. s (broad signal), m (multiplet). Coupling constants (*J*) are given in Hz and refer to H,H-couplings.

^{13}C NMR spectra were recorded at room temperature on Agilent Mercury 400 MHz spectrometers. Spectra were recorded in dichloromethane-d_2, chloroform-d_1 or DMSO-d_6. Chemical shifts

are reported in δ units relative to the solvent signal: dichloromethane-d_2 [δC = 53.84 ppm (central line of the quintet)] chloroform-d_1 [δC = 77.16 ppm (central line of triplet)], DMSO-d_6 [δC = 40.96 ppm (central line of heptet)]. If no coupling constants are given, the multiplicity refers to ^{1}H-decoupled spectra; otherwise, the coupling constants belong to heteroatoms.

Mass spectra (MS): high resolution mass spectroscopy was performed on QExactive spectrometer (ThermoScientific) and low resolution mass spectroscopy was performed on Q-TOF Premier spectrometer (Waters).

Elemental Analyses were performed at the Polish Academy of Sciences, Institute of Organic Chemistry.

IR spectra were recorded on a Perkin-Elmer Spectrum One FTIR spectrometer. Substances were applied as a film, solid or in solution. The obtained data was processed with the software Omni32. Wavenumbers are given in cm^{-1}.

Reagents and Solvents

All reagents were purchased from Sigma-Aldrich, and POCH and used without further purification unless stated otherwise.

PAO 6 (Synfluid® PAO 6 cSt) were purchased from Chevron Phillips Chemical Company LP and purified prior use by filtration through pad of neutral aluminum oxide and purging with argon.

SnatchCat metal scavenger was purchased from Apeiron Synthesis [1–5]

Mo1, **Mo2**, **Mo3** (in paraffin pills or as a powder) was purchased from XiMo and used as received.

Ru1 and **Ru4** was purchased from Merck and used as received

API derivatives was bought from Polpharma

2. Supplementary discussion

2.1. Synthesis of Ruthenium complexes

2.1.1. Synthesis of 3,6-dichloro-1,2-dithiobenzeneethylenodiamine zinc complex (**46**)

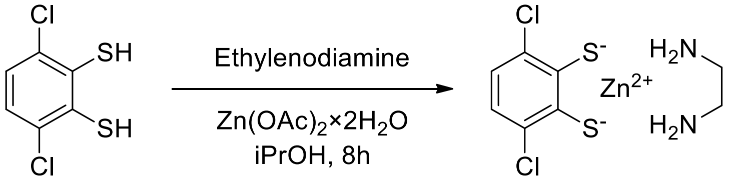

The flask was charged with 3,6-dichloro-1,2-dithiobenzene (1.0 g, 4.74 mmol, 1.0 equiv.), zinc acetate dihydrate (4.16 g, 18.9 mmol, 4.0 equiv.), iPrOH (50 mL) and ethylenediamine (1.9 mL, 1.71 g, 28.4 mmol, 6.0 equiv.). Reaction mixture were stirred in RT for 2 days, and then formed precipitate was collected, washed with methanol (10 mL), hot chloroform (20 mL) and dried in vacuo to obtain product as yellowish solid (1.3 g, 3.89 mmol, 82% yield)

^{1}H NMR (400 MHz, DMSO-*d*$_6$**)** δ 6.78 (s, 2H), 4.07 (br. s, 4H), 2.65 (s, 4H).

^{13}C NMR (101 MHz, DMSO-*d*$_6$**)** δ 147.9, 132.2, 121.9, 40.9.

The spectra correspond to those described in the literature[6]

2.1.2. Synthesis of **Ru2**

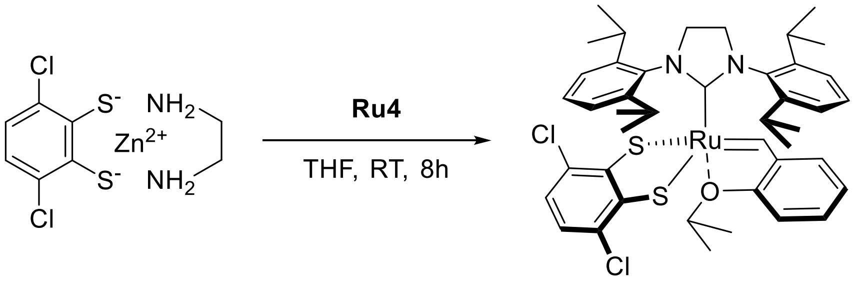

In a glovebox, to a flask charged with 3,6-dichloro-1,2-dithiobenzeneethylenodiamine zinc complex (169 mg, 0.5 mmol, 1.5 equiv.) and **Ru4** (240 mg, 0.34 mmol, 1.0 equiv.), dry THF (20 mL) was added. The reaction mixture was stirred at room temperature for 8 h, concentrated *in vacuo*, and the residue was diluted in dry DCM (50 mL). The formed suspension was filtered through a Celite pad (0.5 cm high, 3 cm diameter), the filtrate was evaporated to dryness and co-evaporated with pentane (3 × 10 mL) three times. Product was obtained as a light brown solid (243 mg, 0.29 mmol, 85% yield).

^{1}H NMR (400 MHz, CD_2Cl_2) δ 14.53 (s, 1H), 7.43 (q, *J* = 7.7 Hz, 2H), 7.37 (t, *J* = 7.8 Hz, 2H), 7.31 (d, *J* = 7.6 Hz, 1H), 7.21 (d, *J* = 7.9 Hz, 1H), 6.95 (d, *J* = 8.4 Hz, 1H), 6.92 (d, *J* = 8.2 Hz, 1H), 6.82 (dt, *J* = 6.9, 3.1 Hz, 2H), 6.75 (d, *J* = 7.7 Hz, 1H), 6.55 (d, *J* = 7.5 Hz, 1H), 4.97 (hept, *J* = 6.1 Hz, 1H), 4.37 (q, *J* = 10.4 Hz, 1H), 4.18 (q, *J* = 10.2 Hz, 1H), 4.02 (q, *J* = 9.7 Hz, 1H), 3.97 – 3.72 (m, 3H), 3.16 – 3.02 (m, 1H), 2.54 – 2.37 (m, 1H), 1.92 (d, *J* = 6.4 Hz, 3H), 1.44 (d, *J* = 5.9 Hz, 3H),

1.38 (d, J = 6.4 Hz, 3H), 1.33 – 1.20 (m, 6H), 1.07 (t, J = 6.8 Hz, 6H), 0.95 (d, J = 6.7 Hz, 3H), 0.55 (d, J = 6.6 Hz, 3H), 0.04 (d, J = 6.6 Hz, 3H).

^{13}C NMR (101 MHz, CD_2Cl_2) δ 259.9, 219.8, 155.4, 153.2, 149.1, 148.3, 146.8, 145.2, 142.6, 141.3, 138.3, 135.5, 130.9, 130.0, 129.5, 128.7, 128.5, 125.5, 125.4, 125.0, 124.9, 124.3, 123.9, 122.9, 122.3, 121.4, 114.5, 76.0, 29.5, 28.9, 28.6, 28.2, 27.0, 26.8, 26.2, 25.5, 23.6, 22.8, 21.3, 20.7, 19.8.

The spectra correspond to those described in the literature.[7]

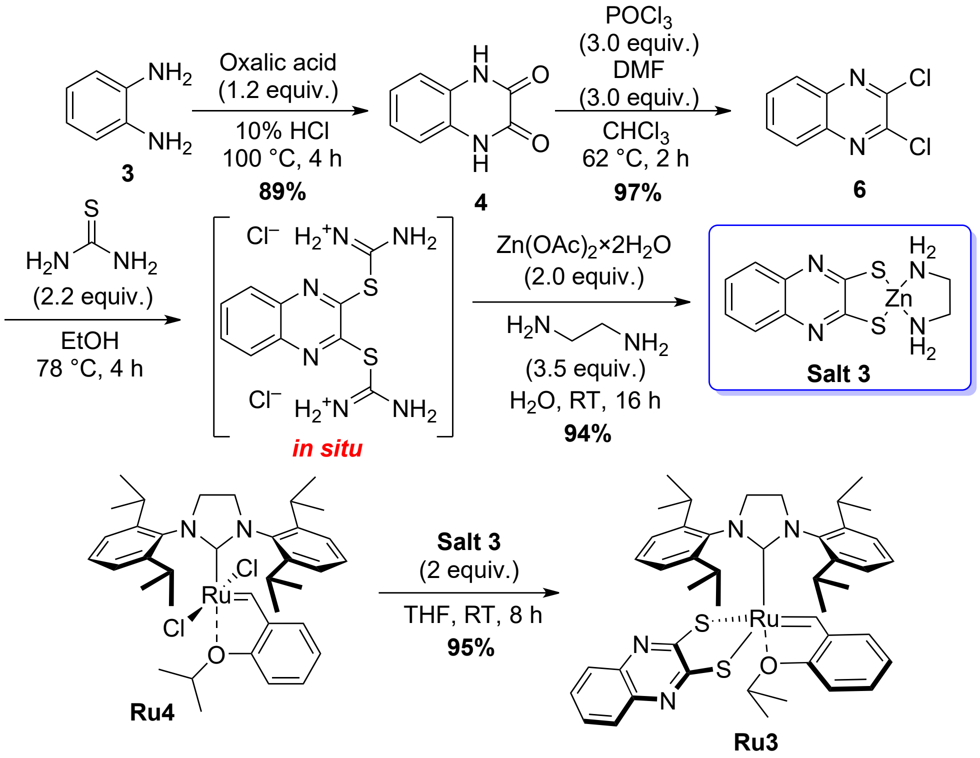

Figure S1. Synthesis pathway for **Ru3**

2.1.3. Synthesis of 1,4-dihydroquinoxaline-2,3-dione (**4**)

The 500 mL flask was charged with *o*-phenylenediamine (**3**, 50 g, 460 mmol, 1.0 equiv.), oxalic acid dihydrate (69.6 g, 552 mmol, 1.2 equiv.) and 10% HCl (300 mL) in water. Reaction mixture was heated at 100 °C for 4 h. Then mixture was cooled down, precipitate was filtered, washed with water (50 mL), EtOH (50 mL), and diethyl ether (50 mL), and evaporated in vacuo to obtain 1,4-dihydroquinoxaline-2,3-dione (**4**) as red crystals (66.2 g, 406 mmol, 89% yield).

^{1}H NMR (400 MHz, DMSO-d_6) δ 11.91 (s, 2H), 7.24 – 6.93 (m, 4H).

^{13}C NMR (101 MHz, DMSO-d_6) δ 155.2, 125.6, 123.0, 115.1.

The spectra correspond to those described in the literature.[8]

2.1.4. Synthesis of 2,3-dichloroquinoxaline (**6**)

The 500 mL flask was charged with 1,4-dihydroquinoxaline-2,3-dione (**4**, 66.6 g, 408 mmol, 1.0 equiv.), chloroform (300 mL) and DMF (89.5 g, 95 mL, 1.225 mol, 3.0 equiv.). Then, $POCl_3$ (114 mL, 188 g, 1.225 mol, 3.0 equiv.) was added dropwise. Next, the obtained mixture was refluxed for 2 h (dissolving of the materials), cooled down and concentrated *in vacuo*. The residue was carefully poured onto ice and diluted with water. The formed precipitate was filtered off, washed with water (100 mL), ethanol (50 mL), diethyl ether (50 mL) and dried on air to obtain 2,3-dichloroquinoxaline (**6**) as grey crystals (79.1 g, 398 mmol, 97% yield).

^{1}H NMR (400 MHz, $CDCl_3$) δ 8.17 – 7.95 (m, 2H), 7.95 – 7.74 (m, 2H)

^{13}C NMR (101 MHz, $CDCl_3$) δ 145.4, 140.6, 131.3, 128.2.

The spectra correspond to those described in the literature.[9]

2.1.5. Synthesis of 2,3-dithoqunoxaline (**10**)

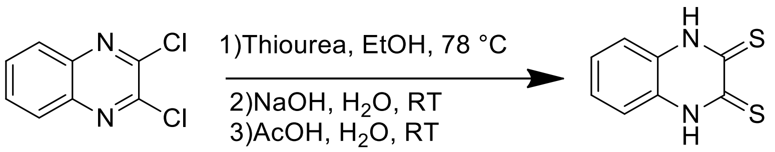

The 50 mL flask was charged with 2,3-dichloroquinoxaline (2.1 g, 10.6 mmol, 1.0 equiv.), thiourea (2.1 g, 27.4 mmol, 2.6 equiv.) and 30 mL of ethanol. After stirring for 4 h at reflux, the solution was cooled to room temperature and the formed precipitate was collected, washed with ethanol (20 mL), transferred to a beaker and suspended in 50 mL of water. Then 20% solution of NaOH in water was added dropwise until pH of solution reach ~10 (dissolving of solid was observed). Then reaction was stirred for additional 10 minutes, and glacial acetic acid was added dropwise until pH reach ~4 which affected forming of dark brown precipitate.. This solid was collected, washed with water (30 mL), ethanol (30 mL), diethyl ether (30 mL) and dried in vacuo to obtain 1.5 g of dark brown powder (7.72 mmol, 73% yield)

^{1}H NMR (400 MHz, DMSO-*d*$_6$) δ 14.25 (s, 1H), 7.40 (dd, *J* = 6.1, 3.4 Hz, 1H), 7.25 (dd, *J* = 6.1, 3.4 Hz, 1H).

^{13}C NMR (101 MHz, DMSO-*d*$_6$) δ 179.7, 128.3, 126.0, 116.0.

The spectra correspond to those described in the literature.[10]

2.1.6. Synthesis of dithioquinoxaline-diamine zinc complex (**8**)

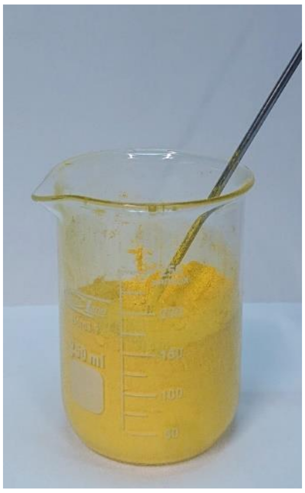

Figure S2. Picture of dithioquinoxaline-diamine zinc complex (**8**)

The 500 mL flask was charged with 2,3-dichloroquinoxaline (**6**, 50 g, 251 mmol, 1.0 equiv.), thiourea (42.1 g, 553 mmol, 2.2 equiv.), and ethanol (300 mL). After stirring for 4 h at reflux, the solution was cooled to room temperature and the formed precipitate was filtered off, washed with ethanol (100 mL), transferred to a 1 L flask and suspended in water (750 mL). Then, to the obtained mixture, ethylenediamine (59.4 mL, 879 mmol, 3.5 equiv.) was added dropwise followed by the addition of zinc acetate dihydrate (110 g, 502 mmol, 2.0 equiv.). The mixture was stirred overnight, the formed precipitate was filtered off, washed with water (200 mL), ethanol (250 mL), diethyl ether (100 mL), and dried to give the title compound as a yellow powder (74.7 g, 235 mmol, 94%).

^{1}H NMR (400 MHz, DMSO-d_6) δ 7.91 – 7.45 (m, 2H), 7.45 – 7.04 (m, 2H), 4.30 (s, 4H), 2.72 (s, 4H).

^{13}C NMR (101 MHz, DMSO-d_6) δ 169.1, 138.2, 125.9, 125.7, 40.3.

HRMS (ESI) Calcd. for $C_{10}H_{13}N_4S_2Zn$ [M+H$^+$]: 316.98676. Found: 316.98704.

EA Calcd. for $C_{10}H_{12}N_4S_2Zn$: C, 37.80; H, 3.81; N, 17.63; S, 20.58. Found: 37.52; 3.73; 17.40; 20.38.

IR (cm^{-1}): 3332, 3247, 3206, 3062, 2950, 2881, 2411, 1591, 1562, 1488, 1457, 1373, 1268, 1158, 1097, 1020, 996, 978, 926, 800, 742, 661, 604, 497, 434.

2.1.7. Synthesis of Ru3

In a glovebox, to a flask charged with 2,3-dithioqunoxalineethylenodiamine zinc complex **8** (593 mg, 1.86 mmol, 2,0 equiv.) and **Ru4** (660 mg, 0.93 mmol, 1.0 equiv.), dry THF (50 mL) was added. The reaction mixture was stirred at room temperature for 8 h, concentrated *in vacuo*, and the residue was diluted in dry DCM (50 mL). The formed suspension was filtered through a Celite pad (0.5 cm high, 3 cm diameter), the filtrate was evaporated to dryness and co-evaporated with pentane (3 × 10 mL) three times. Product was obtained as a dark brown solid with 0.5 equiv. of DCM per formula unit as co-solvent (768 mg, 0.88 mmol, 95% yield).

^{1}H NMR (400 MHz, $CDCl_3$) δ 15.02 (s, 1H), 7.85 (dd, *J* = 8.2, 1.5 Hz, 1H), 7.79 (dd, *J* = 8.1, 1.4 Hz, 1H), 7.47 – 7.30 (m, 7H), 7.28 (dd, *J* = 7.7, 1.6 Hz, 1H), 7.21 (dd, *J* = 7.7, 1.5 Hz, 1H), 6.90 (d, *J* = 8.4 Hz, 1H), 6.82 (td, *J* = 7.4, 0.8 Hz, 1H), 6.73 (dd, *J* = 7.6, 1.6 Hz, 1H), 6.58 (dd, *J* = 7.6, 1.7 Hz, 1H), 4.94 (hept, *J* = 6.2 Hz, 1H), 4.40 – 4.20 (m, 1H), 4.20 – 4.05 (m, 2H), 4.05 –

3.90 (m, 2H), 3.90 – 3.77 (m, 1H), 3.09 (hept, *J* = 6.8 Hz, 1H), 2.39 (hept, *J* = 6.7 Hz, 1H), 1.91 (d, *J* = 6.5 Hz, 3H), 1.39 (d, *J* = 5.9 Hz, 3H), 1.33 (d, *J* = 6.8 Hz, 3H), 1.28 (d, *J* = 7.0 Hz, 3H), 1.26 (d, *J* = 6.9 Hz, 2H), 1.04 (d, *J* = 6.8 Hz, 3H), 0.99 (d, *J* = 6.3 Hz, 3H), 0.92 (d, *J* = 6.7 Hz, 3H), 0.67 (d, *J* = 6.6 Hz, 3H), 0.01 (d, *J* = 6.7 Hz, 3H).

^{13}C NMR (101 MHz, THF-*d*$_8$) δ 262.2, 220.9, 171.5, 163.4, 156.5, 150.2, 149.3, 147.6, 1456.0, 142.1, 139.2, 139.0, 138.9, 136.6, 131.1, 130.7, 129.3, 129.3, 128.8, 128.0, 127.4, 126.2, 126.2, 126.0, 125.9, 125.6, 125.3, 125.1, 124.5, 123.0, 115.5, 29.9, 29.4, 29.28, 29.0, 27.3, 26.9, 26.6, 24.2, 22.8, 22.2, 20.9, 20.3, 14.3.

HRMS (ESI) Calcd. for $C_{45}H_{55}N_4ORuS_2$ [M+H]$^+$: 833.28553. Found: 833.28593.

EA Calcd. for $C_{45}H_{54}N_4ORuS_2$ × 0.5 CH_2Cl_2: C, 62.49; H, 6.34; N, 6.41; S, 7.33. Found: C, 62.45; H, 6.50; N, 6.27; S, 7.38.

IR (cm^{-1}) 3061, 2961, 2924, 2866, 2656, 2451, 2323, 2289, 1682, 1589, 1474, 1453, 1440, 1408, 1385, 1364, 1324, 1255, 1236, 2272, 1119, 1094, 1046, 1016, 919, 802, 752, 621, 599, 566, 457, 427.

2.2. Catalyst stability

2.2.1. Stability at solid state in air at room temperature

Solid sample of **Ru2** was taken out from the glovebox and stored for 24 h in open vessel in air. After this time they were taken back to the glovebox, dissolved in degassed CD_2Cl_2, and their NMR spectra were recorded. The decomposition of the catalysts was estimated by ratio of alkylidene signal (at 15.02 ppm) and aldehyde signal (product of the oxidative decomposition of the catalyst at 14.47 ppm). After 24 h **Ru3** shown ca. 5% decomposition

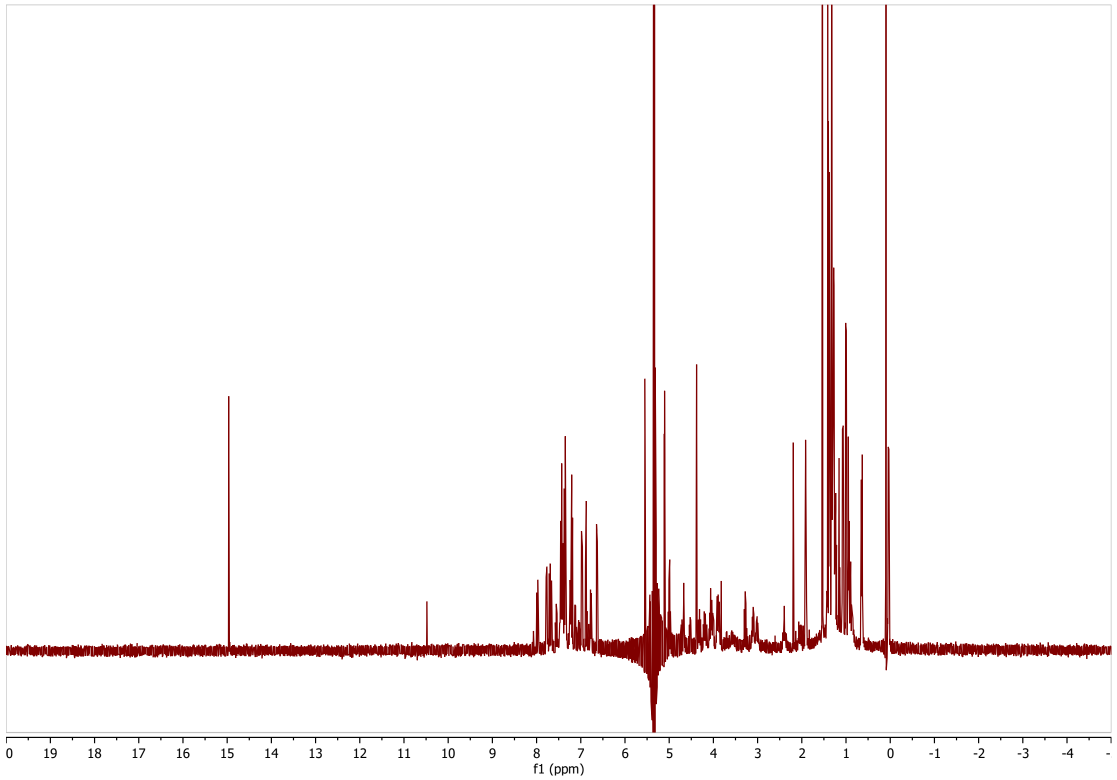

Figure S3. ^{1}H NMR (400 MHz, CD_2Cl_2)Spectrum of **Ru3** after 24 h of exposure to atmospheric oxygen in solid state.

2.2.2. Stability at 60 °C

A tightly closed screw-cap NMR tube with a solution of Ru-catalyst (0.005 mmol) and anthracene (internal standard, 0.05 mmol) in THF-d_8 (0.5 mL) was examined by ^{1}H NMR at time t = 0 h and then placed in an oil bath set at 60 °C. Samples were measured after a given period of time. The degradation of the catalyst was observed by the disappearance of the benzylidene signal relative to the anthracene in corresponding ^{1}H NMR spectra.

% of remaining **Ru2**	Time [h]	% of remaining **Ru3**	Time [h]
100	0	100	0
96	24	97	24
93	48	94	48
85	104	88	104
82	128	85	128
79	152	83	152
76	176	80	176
70	242	75	242

Table S1. Results for stability comparison of **Ru2** and **Ru3** in 60 °C

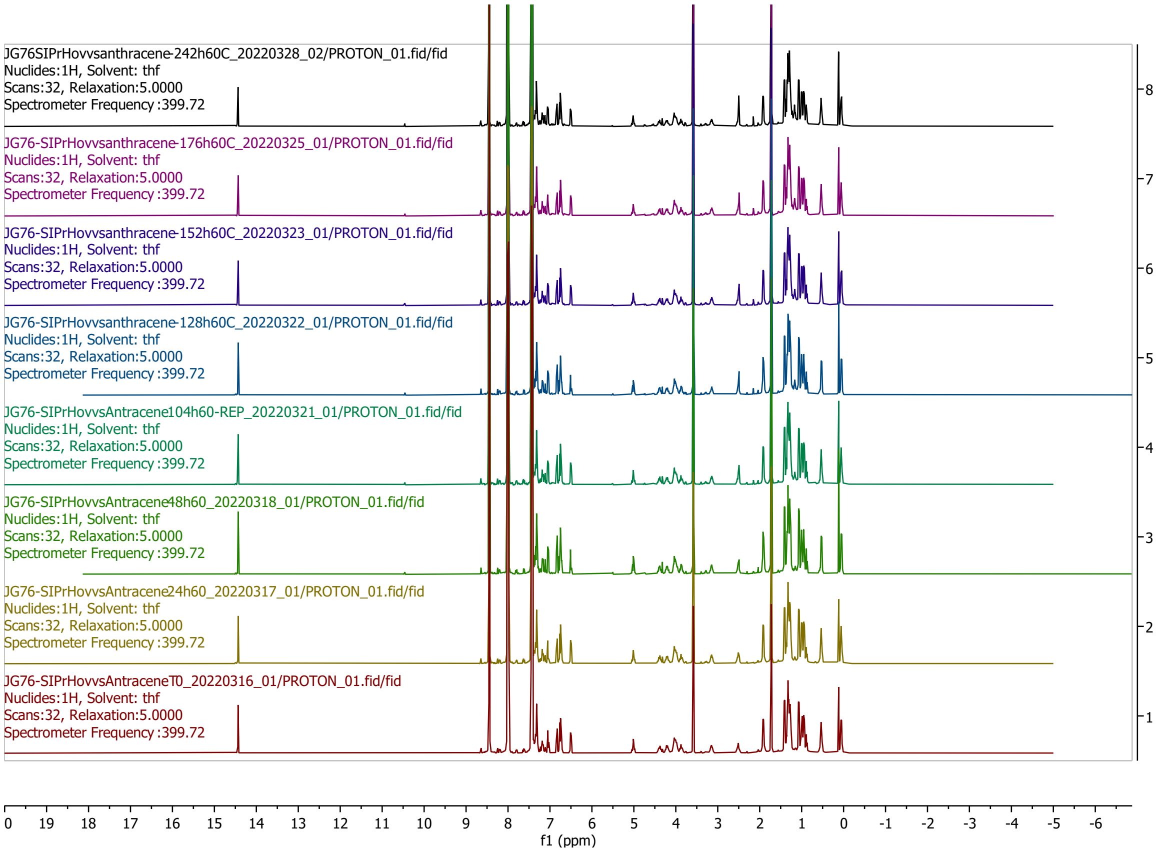

Figure S4. ^{1}H **NMR (400 MHz, THF-*d₈*)**Stacked spectra for stability test of **Ru2** at 60 °C.

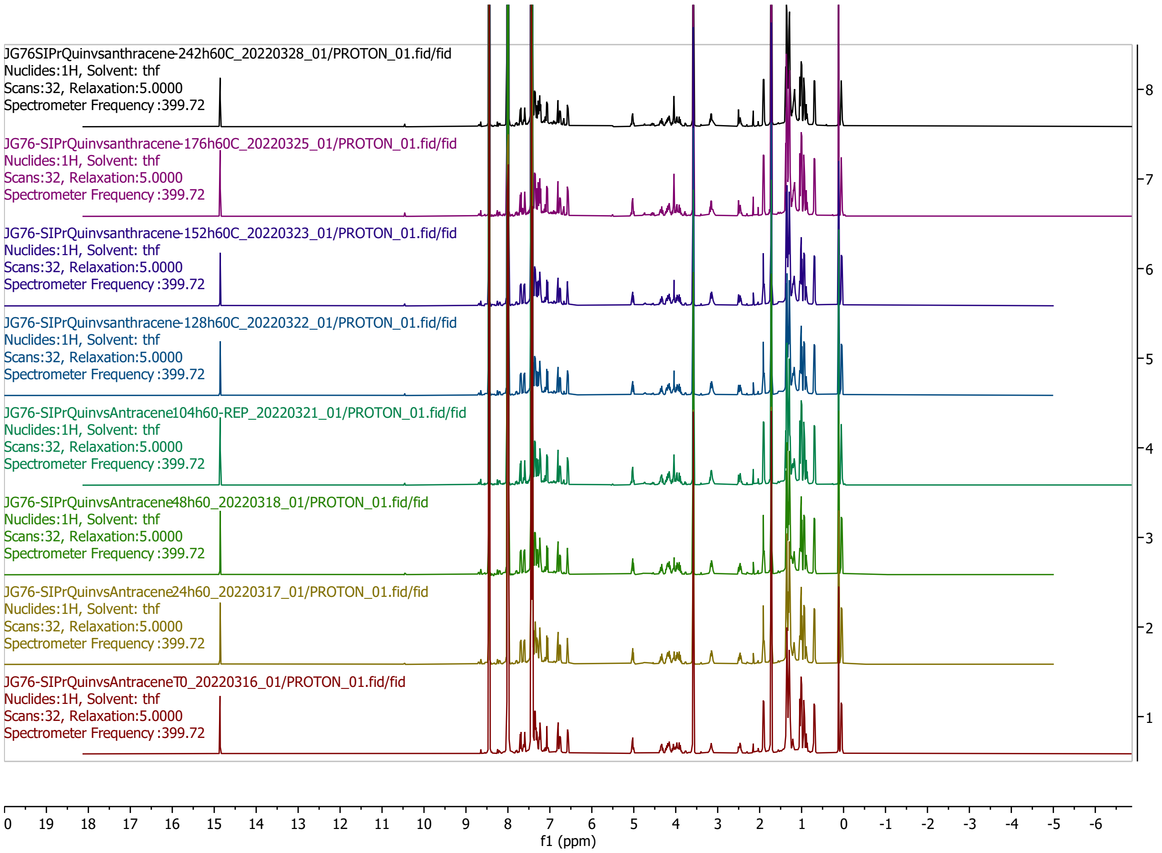

Figure S5. ^{1}**H NMR (400 MHz,** THF-d_8**)** Stacked spectra for stability test of **Ru3** at 60 °C.

2.2.3. Stability at 110 °C

A tightly closed screw-cap NMR tube with a solution of Ru-catalyst (0.005 mmol) and anthracene (internal standard, 0.05 mmol) in THF-d_8 (0.5 mL) was examined by ^{1}H NMR at time t = 0 h and then placed in an oil bath set at 110 °C. Samples were measured after a given period of time. The degradation of the catalyst was observed by the disappearance of the benzylidene signal relative to the anthracene in corresponding ^{1}H NMR spectra.

% of remaining **Ru2**	Time [h]	% of remaining **Ru3**	Time [h]
100	0	100	100
89	1	97	92
75	2	94	88
41	3	88	83
19	4	85	69
9	5	83	61

Table S2. Results for stability comparison of **Ru2** and **Ru3** in 110 °C

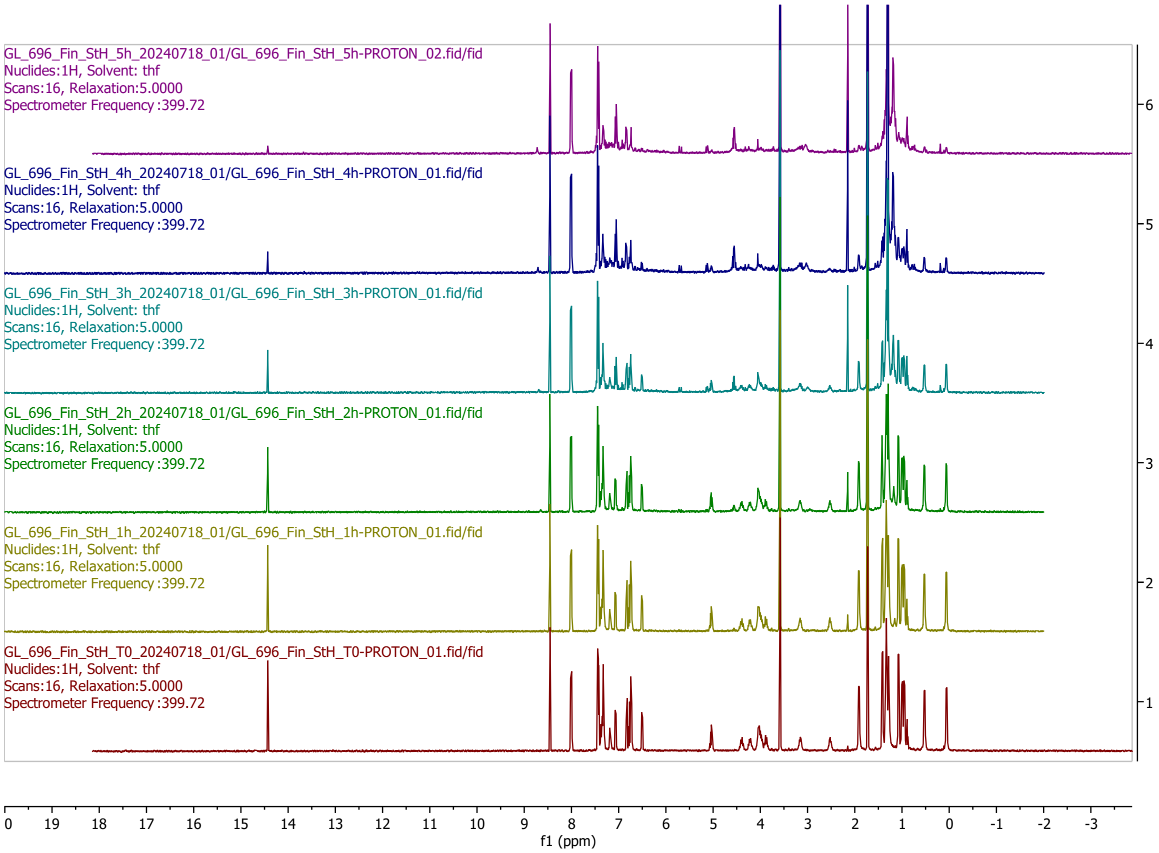

Figure S6. ^{1}**H NMR (400 MHz, THF-*d*$_8$)** Stacked spectra for stability test of **Ru2** at 110 °C.

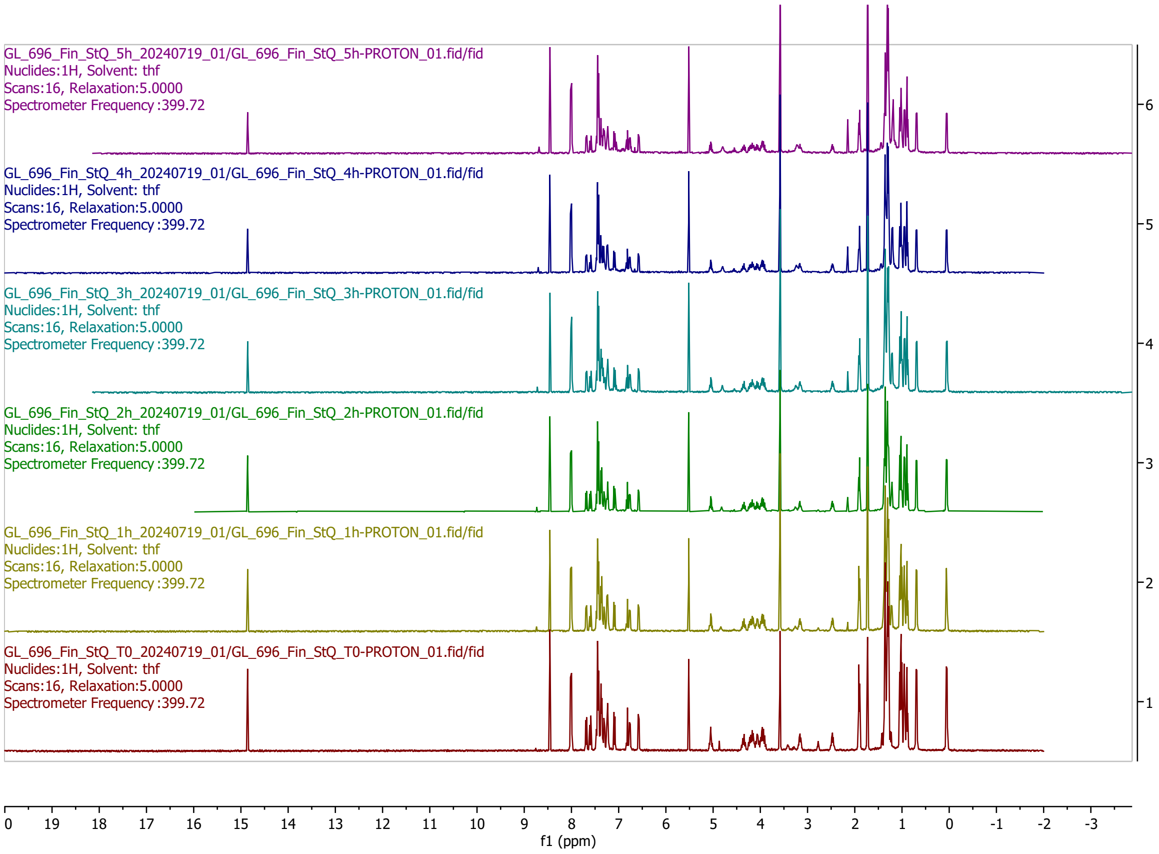

Figure S7. **^{1}H NMR (400 MHz, THF-*d*$_8$)** Stacked spectra for stability test of **Ru2** at 110 °C.

2.3. General procedure for mRCM in reactive distillation conditions

In a glovebox, in a 10 mL flask an appropriate amount of Ru complex was placed as a solid and covered with PAO6 (2.5 mL). Then, a corresponding substrate (0.5 mmol) was added to the obtained mixture, the flask was sealed, transferred out of the glovebox, and attached to the reactive distillation setup (Hickman adapter cooled with dry ice/liquid nitrogen mixture, connected to Oil Diffusion Pump).[11,12] The flask was immediately evacuated using an oil pump (pump nominal pressure 10^{-3} mbar) followed by a diffusion pump (pump nominal pressure 10^{-6} mbar) until the pressure equilibrates. After the setup was evacuated with a diffusion pump, the flask was placed in a preheated oil bath. Reaction was carried at given temperature (110-150 °C) for 8 h, and then distillate was collected and purified on silica gel column (*n*-hexane to 5% EtOAc in *n*-hexane)

2.3.1. Synthesis of (*Z*)-oxacyclohexadec-10-en-2-one (**2**)

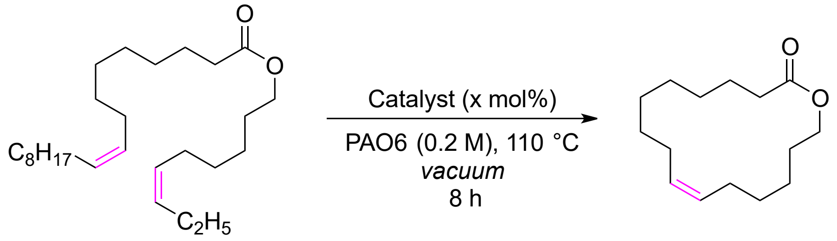

The reaction was carried out following the general procedure at 110 °C, with a loading of the catalyst described in the table below. *Z*/*E* ratio was determined by GC.

Entry	Loading (mol%)	Cat.	Isolated yield (%)	*Z* (%)
1	10	**Ru2**	52	58
2	10	**Ru3**	84	93
3	1.0	**Ru3**	76	9
4	0.5	**Ru3**	78	98
5	0.5	**Ru3**	93	97

Table S3. Results for synthesis of (Z)-oxacyclohexadec-10-en-2-one

^{1}H NMR (400 MHz, $CDCl_3$) δ 5.60 – 4.99 (m, 2H), 4.33 – 3.53 (m, 2H), 2.54 – 2.25 (m, 2H), 2.21 – 1.91 (m, 4H), 1.73 – 1.52 (m, 4H), 1.39 – 1.33 (m, 4H), 1.33 – 1.20 (m, 8H).

^{13}C NMR (101 MHz, $CDCl_3$) δ 173.9, 130.2, 129.9, 64.4, 35.3, 28.9, 28.5, 28.2, 28.2, 28.1, 27.7, 27.4, 26.3, 26.2, 24.9.

The spectra correspond to those described in the literature.[13]

2.3.2. Synthesis of (*Z*)-oxacyclotridec-10-en-2-one (**11**, Yuzu Lactone)

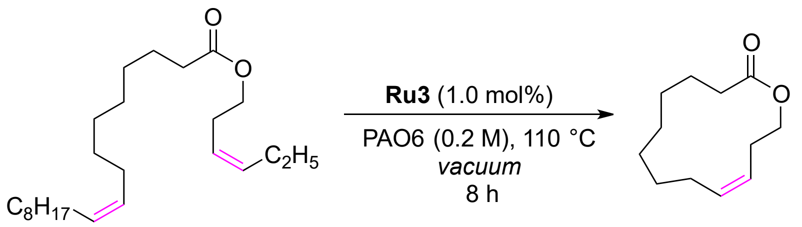

The reaction was carried out following the general procedure with 1.0 mol% of **Ru3** at 110 °C. The product was obtained in 66% yield and *Z*/*E* = 99:1 (determined by GC).

^{1}H NMR (400 MHz, $CDCl_3$) δ 5.60 – 5.11 (m, 2H), 4.68 – 4.01 (m, 2H), 2.45 – 2.33 (m, 2H), 2.30 – 2.20 (m, 2H), 2.15 – 1.99 (m, 2H), 1.76 – 1.54 (m, 2H), 1.54 – 1.41 (m, 2H), 1.41 – 1.31 (m, 2H), 1.31 – 1.22 (m, 2H), 1.22 – 1.13 (m, 2H).

^{13}C NMR (101 MHz, $CDCl_3$) δ 174.6, 132.2, 127.1, 64.1, 35.3, 29.7, 27.5, 27.2, 26.0, 25.8, 24.5, 23.5.

The spectra correspond to those described in the literature.[14]

2.3.3. Synthesis of (*Z*)-oxacyclononadec-10-ene (**12**)

Substrate synthesis ((Z)-1-(((Z)-octadec-9-en-1-yl)oxy)octadec-9-ene)

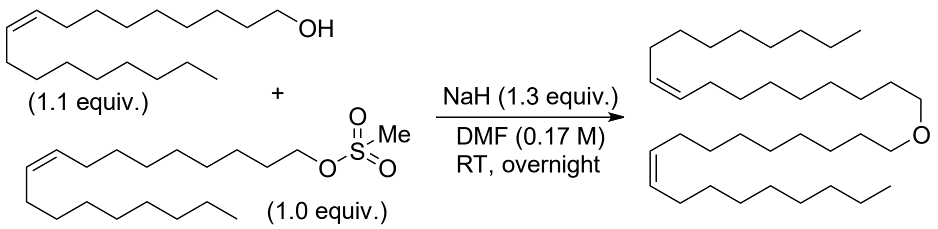

A 250 mL round-bottom flask was charged with sodium hydride (0.28 g, 11.3 mmol, 1.3 equiv.) and anhydrous DMF (50 mL). To the resulting suspension (*Z*)-octadec-9-en-1-ol (2.56 g, 3.0 mL 9.52 mmol, 1.1 equiv.) was added dropwise at room temperature. After completion of the addition, the mixture was stirred for 30 min at room temperature and (*Z*)-octadec-9-en-1-yl methanesulfonate (3 g, 8.66 mmol, 1.0 equiv.) was added dropwise. The final reaction mixture was stirred at room temperature overnight. Then, water (50 mL) was added to the flask, the solution was transferred to a separation funnel, and the product was extracted with hexane (3 × 50 mL). The combined organic layers were washed with water (50 mL), brine (50 mL), dried over sodium sulphate, and filtered through silica gel (SiO_2, *n*-hexane as eluent) to give a colourless oil (3.0 g, 5.78 mmol, 67% yield).

^{1}H NMR (400 MHz, $CDCl_3$) δ 5.77 – 4.89 (m, 4H), 3.38 (t, *J* = 6.7 Hz, 4H), 2.08 – 1.95 (m, 8H), 1.67 – 1.50 (m, 4H), 1.46 – 1.14 (m, 44H), 0.97 – 0.78 (m, 6H).

^{13}C NMR (101 MHz, $CDCl_3$) δ 129.9, 129.8, 71.0, 31.9, 29.8, 29.8, 29.8, 29.5, 29.5, 29.5, 29.3, 29.3, 27.2, 27.2, 26.2, 22.7, 14.1.

The spectra correspond to those described in the literature.[15]

mRCM of ((*Z*)-1-(((*Z*)-octadec-9-en-1-yl)oxy)octadec-9-ene)

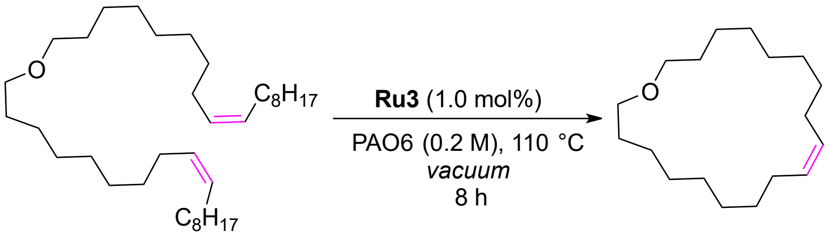

The reaction was carried out following the general procedure with 1.0 mol% of **Ru3** at 110 °C. The product was obtained in 68% yield and *Z*/*E* = 98:2 (determined by GC).

^{1}H NMR (400 MHz, $CDCl_3$) δ 5.29 (ddd, *J* = 5.6, 4.4, 1.1 Hz, 2H), 3.40 (dd, *J* = 5.9, 5.0 Hz, 4H), 2.80 – 1.73 (m, 4H), 1.64 – 1.47 (m, 4H), 1.47 – 1.13 (m, 20H).

^{13}C NMR (101 MHz, $CDCl_3$) δ 130.3, 70.1, 29.7, 29.5, 29.3, 29.1, 28.1, 26.8, 26.3.

HRMS (ESI TOF *m*/*z*) calculated for $C_{19}H_{34}O$ $[M+H]^+$: 267.2682 , Found: 267.2680.

EA: calculated for $C_{18}H_{34}O$: C, 81.13; H, 12.86; Found C, 81.13; H, 12.84.

IR (cm^{-1}): 2922, 2851, 1117.

2.3.4. Synthesis of (*Z*)-cycloheptadec-9-en-1-one (**13**, Civetone)

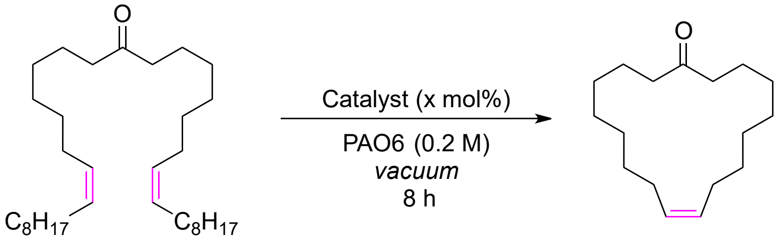

The reaction was carried out following the general procedure with the temperature and catalyst loading described in the table below. *Z*/*E* ratio was determined by GC.

Entry	Loading, mol%	Temp.	Cat.	Yield (%)	*Z*, %
1	1.0	110 °C	**Ru3**	54	96
2	0.5	110 °C	**Ru3**	54	98
3	0.5	110 °C	**Ru1**	13	83
3	0.5	130 °C	**Ru3**	45	96
4	0.5	150 °C	**Ru3**	27	96

Table S4. Results for Synthesis of (Z)-cycloheptadec-9-en-1-one

^{1}H NMR (400 MHz, CDCl$_3$)** δ 5.46 – 5.18 (m, 2H), 2.52 – 2.18 (m, 4H), 2.08 – 1.91 (m, 4H), 1.66 – 1.51 (m, 4H), 1.49 – 1.00 (m, 16H).
^{13}C NMR (101 MHz, CDCl$_3$)) δ 212.6, 130.1, 42.4, 29.0, 28.6, 28.2, 28.1, 26.7, 23.8.
The spectra correspond to those described in the literature.[14]

2.4. Self-metathesis "dimerisation" reactions

2.4.1. Synthesis of (*Z*)-hex-3-ene-1,6-diol (**16**)

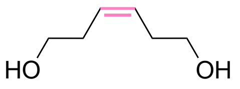

Ru2 (20 mg, 24 µmol, 0.07 equiv./0.7 mol%) or **Ru3** (20 mg, 23 µmol, 0.01 equiv./0.7 mol%) and (*Z*)-hex-3-ene-1-ol (345 mg, 3.44 mmol, 1.0 equiv.) were placed in a Schlenk flask and the mixture was stirred at 40 °C for 30 min. at 50 mbar (to remove the emerging hex-3-ene by-product) and another 30 min at 60 °C. The reaction mixture was diluted with EtOAc (5 mL) and SnatchCat® (23 mg, 106 µmol, 4.4 equiv.) was added. The product was purified by column chromatography (EtOAc) and was isolated as a colourless oil.
Yield from the reaction catalysed by **Ru3** is 82% (***Z/E*** = 96:4).
Yield from the reaction catalysed by **Ru2** is 92% (***Z/E*** = 97:4).
^{1}H NMR (400 MHz, CDCl$_3$) for *Z* isomer δ 5.52 (m, 2H), 3.63 (t, *J* = 6.0 Hz, 4H), 3.28 (bs, 2H), 2.33 (m, 4H).
^{13}C NMR (101 MHz, CDCl$_3$) for *Z* isomer δ 129.2, 61.9, 30.63.
The spectra correspond to those described in the literature.[16]

2.4.2. Synthesis of (*Z*)-dodec-6-ene-1,6-diol (**18**)

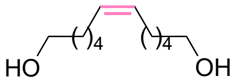

A Schlenk flask was charged with (*Z*)-6-nonenol (5.0 g, 35.2 mmol) and Ru catalyst (100 ppm, 3.5 µmol, 3 mg of **Ru2** or 2.9 mg of **Ru3**) in an argon flow. The resulting reaction mixture was stirred under vacuum (2 × 10^{-3} mbar) for 1 h. Then, dry DCM (20 mL) and activated charcoal (0.5 g) were added, and the reaction mixture was stirred at room temperature for 1 h. Next, the mixture was filtered through Celite®, concentrated *in vacuo*, and purified by column chromatography (MeOH in DCM from 0 to 10%) to obtain a colourless solid.
Yield from the reaction catalysed by **Ru3** is 95% (***Z/E*** = 96:4).
Yield from the reaction catalysed by **Ru2** is 92% (***Z/E*** = 97:3).

^{1}H **NMR (400 MHz, $CDCl_3$) for *Z* isomer** δ 5.26 (ddd, *J* = 5.6, 4.3, 1.1 Hz, 1H), 3.60 (d, *J* = 1.6 Hz, 1H), 3.48 (t, *J* = 6.8 Hz, 2H), 2.27 – 1.77 (m, 2H), 1.64 – 1.37 (m, 2H), 1.36 – 1.15 (m, 4H).

^{13}C **NMR (101 MHz, $CDCl_3$) for *Z* isomer** δ 129.7, 62.3, 32.4, 29.4, 27.0, 25.3.

The spectra correspond to those described in the literature.[17]

2.4.3. Synthesis of (*Z*)-5-hydroxypent-2-en-1-yl acetate (**20**) – self-CM reaction and subsequent CM reaction

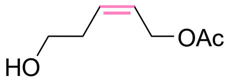

Ru2 (12 mg, 14 μmol, 0.01 equiv./1 mol%) or **Ru3** (12 mg, 14 μmol, 0.01 equiv./1 mol%) and (*Z*)-hex-3-ene-1-ol (134 mg, 1.39 mmol, 1.0 equiv.) were placed in a Schlenk flask and stirred at 40 °C for 30 min. at 50 mbar (to remove the emerging hex-3-ene by-product) and another 30 min at 60 °C (to remove the remaining substrate). Then, (*Z*)-1,4-acetoxybut-2-ene (119 mg, 0.69 mmol, 0.5 equiv.) was added and the mixture was stirred at 60 °C for 1 hour. The black residue was diluted in EtOAc (2 mL) and SnatchCat® (14 mg, 62 μmol, 0.04 equiv.) was added. The product was isolated using column chromatography (SiO_2, 50% EtOAc/*n*-hexane) as a colourless liquid.

Yield from the reaction catalysed by **Ru2** is 54% (***Z/E*** = 99:1).

Yield from the reaction catalysed by **Ru3** is 52% yield (***Z/E*** = 98:2).

^{1}H **NMR (400 MHz, $CDCl_3$) for *Z* isomer** δ 5.68 (m, 2H), 4.64 (d, *J* = 6.1 Hz, 2H), 3.67 (m, 2H,), 2.40 (m, 2H), 2.05 (s, 3H), 1.79 (bs, 1H).

^{13}C **NMR (101 MHz, $CDCl_3$) for *Z* isomer** δ 171.3, 131.4, 126.5, 61.9, 60.5, 31.1, 21.1.

The spectra correspond to those described in the literature.[18]

2.4.4. Synthesis of (*Z*)-dodec-6-enedial (**22**)

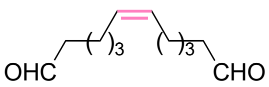

Schlenk flask was charged with (*Z*)-6-nonenal (3.59 g, 4.26 mL, 24 mmol) and Ru catalyst (0.1 mol%, 24μmol, 20 mg of **Ru3** in an argon flow. The resulting reaction mixture was stirred under vacuum (10 mbar) for 2 h. After this time reaction vessel was attached to high vacuum (2×10^{-3} mbar) and heated up to remove residual substrate. Crude product was filtered through silica gel column (SiO_2, 10% EtOAc/*n*-hexane) to obtain 1.60 g of a colourless viscous oil (8.15 mmol, 68 % yield, ***Z/E*** = 99:1) .

^{1}H **NMR (400 MHz, $CDCl_3$) for *Z* isomer** δ 9.72 (t, *J* = 1.9 Hz, 1H), 5.96 – 5.08 (m, 2H), 2.40 (td, *J* = 7.3, 1.8 Hz, 4H), 2.01 (td, *J* = 7.4, 5.4 Hz, 4H), 1.60 (p, *J* = 7.4 Hz, 4H), 1.34 (dq, *J* = 10.1, 7.5 Hz, 4H).

^{13}C NMR (101 MHz, $CDCl_3$) for *Z* isomer δ 202.7, 129.6, 43.7, 29.1, 26.9, 21.6.

HRMS (ESI TOF *m/z*) calculated for $C_{19}H_{34}O$ $[M+H]^+$: 197.1536, Found: 197.1536.

IR (cm^{-1}): 3005, 2930, 2858, 2720, 1721, 1459, 1409, 1390

2.5. Cross-Metathesis Reactions

2.5.1. Solvents scope

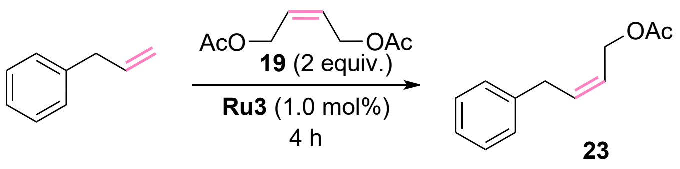

In a glovebox, a solution of allylbenzene (11.8 mg, 0.1 mmol, 1.00 equiv.), (*Z*)-1,4-diacetoxybut-2-ene (34.4 mg, 0.2 mmol, 2 equiv.), and tetradecane (1.9 mg, 0.01 mol, 0.10 equiv., a reference standard) in a dry solvent (0.3 mL) was placed in a vial which was subsequently charged with a solution of catalyst (1.0 mol% in 0.2 mL). The reaction mixture was stirred at a corresponding temperature for 4 h. Then, an aliquot (200 µL) of the reaction mixture was analysed by GC. When catalyst was insoluble in a given solvent, it was placed as a solid, and 0.2 mL of a corresponding solvent was added afterwards (toluene, DMC).

Table S5. Cross-metathesis of allylbenzene and (*Z*)-1,4-diacetoxybut-2-ene; optimisation of the solvent and temperature.

T, [°C]	Solvent	Conversion, [%]	Yield, [%]	*Z/E*
40	THF	51	51	>99:1
	DCE	43	36	90:10
	EtOAc	37	30	92:8
	Toluene	19	13	97:3
	DMC	49	40	>99:1
	4-MeTHP	44	36	99:1
	Perfluorotoluene	11	12	89:11
	2-MeTHF	11	12	89:11
90	THF	39	36	>99:1
	DCE	69	54	61:49
	EtOAc	45	36	87:13
	Toluene	33	21	96:4
	DMC	55	41	92:8
	4-MeTHP	56	42	89:11
	Perfluorotoluene	22	16	91:9
	2-MeTHF	22	16	91:9
120	THF	58	42	>99:1
	DCE	62	46	69:31
	EtOAc	44	35	88:12

Toluene	25	16	94:6
DMC	52	40	94:6
4-MeTHP	55	40	93:7
Perfluorotoluene	14	13	82:18
2-MeTHF	14	13	82:18

4-MeTHP – 4-methyltetrahydropyran; DMC – dimethyl carbonate; 2-MeTHF – 2-methyltetrahydrofuran.

2.5.2. General procedure for cross-metathesis reactions

The reactions were carried out in a glovebox. A 4 mL vial was charged with a mixture of a corresponding alkene cross-metathesis partner (0.2 mmol, 1.0 equiv.), and (*Z*)-2-butene-1,4-diol (0.4 mmol, 2.0 equiv.) or (*Z*)-1,4-diacetoxy-2-butene (0.4 mmol, 2.0 equiv.), and tetradecane (0.04 mmol, 0.1 equiv.; used as an internal standard) in dry THF. To the resulting solution **Ru2** or **Ru3** were added to get a final 0.2 M solution of corresponding cross-metathesis partner. After 4 hours of stirring at a given temperature the reaction was quenched with SnatchCat®[5] (4 equiv. *vs* Ru) and the products were isolated by means of column chromatography.

2.5.3. (*Z*)-4-(4-methoxyphenyl)but-2-en-1-yl acetate (**24**)

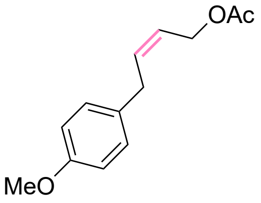

The reaction was carried out according to the general procedure with (*Z*)-diacetoxy-2-butene (313 mg, 1.82 mmol, 2.0 equiv.), 1-allyl-4-methoxybenzene as an alkene cross-metathesis partner (135 mg, 0.91 mmol, 1.0 equiv.), and 4 mol% of **Ru3** or **Ru2** at 60 °C. The product was isolated by column chromatography (SiO_2, 20% to 40% EtOAc/*n*-hexane) as a colourless oil.

Yield from the reaction catalysed by **Ru3** is 53% (***Z/E*** = 99:1).

Yield from the reaction catalysed by **Ru2** is 52% (***Z/E*** = 99:1).

^{1}H NMR (400 MHz, $CDCl_3$) for *Z* isomer δ 7.10 (d, *J* = 8.7 Hz, 2H.), 6.84 (d, *J* = 8.6 Hz, 2H), 5.86 – 5.75 (m, 1H), 5.71 – 5.59 (m, 1H), 4.74 (dd, *J* = 6.9, 1.3 Hz, 2H), 3.78 (s, 3H,), 3.41 (d, *J* = 7.6 Hz, 2H), 2.08 (s, 3H).

^{13}C NMR (101 MHz, $CDCl_3$) for *Z* isomer δ 171.0, 158.1, 133.9, 131.9, 129.3, 129.3, 123.9, 114.0, 114.0, 60.3, 55.3, 32.9, 21.0.

The spectra correspond to those described in the literature.[19]

2.5.4. Synthesis of (*Z*)-7-hydroxyhept-2-en-1-yl acetate (**25**)

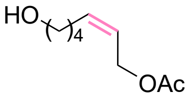

The reaction was carried out according to the general procedure with (*Z*)-diacetoxy-2-butene (400 mg, 2.32 mmol, 2.0 equiv.), (*Z*)-6-octen-1-ol as alkene cross-metathesis partner (149 mg, 1. mmol, 1.0 equiv.), and 0.2 mol% of **Ru3** or **Ru2** at 60 °C.

The product was isolated by column chromatography (SiO_2, 20% EtOAc/*n*-hexane to EtOAc) as a colourless oil.

Yield from the reaction catalysed by **Ru3** is 75% (***Z/E*** = 96:4).

Yield from the reaction catalysed by **Ru2** is 70% (***Z/E*** = 99:1).

^{1}H NMR (400 MHz, $CDCl_3$) for *Z*-isomer δ 5.59 (m, 2H), 4.61 (d, *J* = 6.5 Hz, 2H), 3.65 (t, *J* = 8.0 Hz, 2H), 2.15 (dt, *J* = 8.0 Hz, 8.0 Hz, 2H), 2.06 (s, 3H), 1.51 (m, 5H).

^{13}C NMR (101 MHz, $CDCl_3$) for *Z*-isomer δ 171.2, 135.1, 123.8, 62.8, 60.5, 32.3, 27.3, 25.7, 21.2.

The spectra correspond to those described in the literature.[20]

2.5.5. Synthesis of (*Z*)-4-phenylbut-2-en-1-yl benzoate (**26**)

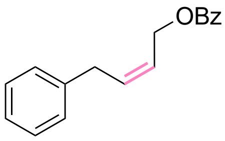

The reaction was carried out according to general procedure with 1 of (*Z*)-phenoxy-2-butene (19 mg, 0.4 mmol, 2.0 equiv.), allylbenzene as alkene cross-metathesis partner (24.1 mg, 0.2 mmol, 1.0 equiv.) and 5 mol% of **Ru3** or **Ru2** at RT. The product was isolated by column chromatography (SiO_2, 0% to 40% EtOAc/*n*-hexane) as a colourless oil.

Yield from the reaction catalysed by **Ru3** is 53% (***Z/E*** = 97:3).

Yield from the reaction catalysed by **Ru2** is 53% (***Z/E*** = 95:5).

^{1}H NMR (400 MHz, $CDCl_3$) δ 8.07 (dd, *J* = 8.5, 1.3 Hz, 2H), 7.61 – 7.54 (m, 1H), 7.45 (t, *J* = 7.5 Hz, 2H), 7.35 – 7.28 (m, 2H), 7.24 – 7.22 (m, 3H), 5.96 – 5.77 (m, 2H), 5.04 – 4.96 (m, 2H), 3.56 (d, *J* = 7.4 Hz, 2H).

^{13}C NMR (101 MHz, $CDCl_3$) δ 166.5, 139.8, 133.7, 133.0, 129.7, 128.6, 128.5, 128.4, 126.2, 124.3, 60.7, 33.9.

The spectra correspond to those described in the literature.[21]

2.5.6. Synthesis (*Z*)-9-hydroxynon-7-enal (**27**)

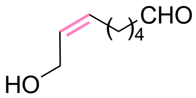

The reaction was carried out according to the general procedure with (*Z*)-2-butene-1,4-diol (248 mg, 2.81 mmol, 2.0 equiv.), (*Z*)-6-nonenal as an alkene cross-metathesis partner (197 mg, 1.41 mmol, 1.0 equiv.), and 2 mol% of **Ru3** or **Ru2** at 60 °C. The product was isolated by column chromatography (SiO_2, 20% EtOAc/*n*-hexane to EtOAc) as a colourless oil.

Yield from the reaction catalysed by **Ru3** is 52% (***Z/E*** = 97:3).

Yield from the reaction catalysed by **Ru2** is 57% (***Z/E*** = 96:4).

^{1}H NMR (400 MHz, $CDCl_3$) for *Z* isomer δ 9.76 (s, 1H), 5.70 – 5.53 (m, 2H), 4.19 (dd, *J* = 6.8, 1.2 Hz, 2H), 2.44 (td, *J* = 7.3, 1.7 Hz, 2H), 2.10 (qd, *J* = 7.4, 1.4 Hz, 2H), 1.75 – 1.56 (m, 2H), 1.50 – 1.32 (m, 3H).
^{13}C NMR (101 MHz, $CDCl_3$) δ 202.8, 132.2, 129.1, 58.6, 43.8, 29.1, 27.2, 21.6.
HRMS (ESI TOF *m/z*) calculated for $C_8H_{14}O_2$ $[M+H]^+$: 143.1067 , Found: 143,1065.
IR (cm^{-1}): 3378, 2924, 2857, 1720, 1460, 1435, 1352, 1105, 996, 967.

2.6. Synthesis of (*Z*)-tridec-2-en-1-ol

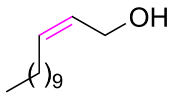

The reaction was carried out according to the general procedure with (Z)-2-butene-1,4-diol (35 mg, 0.4 mmol, 2.0 equiv.), dodecene as an alkene cross-metathesis partner (33 mg, 0.2 mmol, 1.0 equiv.), and 5 mol% of **Ru3** or **Ru2** at 40 °C. The product was isolated by column chromatography (SiO_2, 20% EtOAc/*n*-hexane) as a colourless oil.
Yield from the reaction catalysed by **Ru3** is 45% (***Z/E*** = 98:2).
Yield from the reaction catalysed by **Ru2** is 49% (***Z/E*** = 99:1).
^{1}H NMR (400 MHz, $CDCl_3$) of *Z*-isomer (major) δ 5.72 – 5.44 (m, 2H), 4.19 (d, *J* = 6.1 Hz, 2H), 2.13 – 2.00 (td, *J* = 8.0 Hz, 8.0 Hz, 2H), 1.33 – 1.20 (m, 18H), 0.88 (t, *J* = 6.0 Hz 3H).
^{13}C NMR (101 MHz, $CDCl_3$) of *Z*-isomer (major) δ 133.5, 128.4, 58.8, 32.1, 29.8 (3C), 29.6, 29.5, 29.4, 27.6, 22.8, 14.3.
The spectra correspond to those described in the literature.[13]

2.7. Cross-Metathesis Reactions of API Derivatives

General procedure for cross-metathesis reactions of API derivatives: The reactions were carried out in a glovebox. In a 4 mL vial, to the mixture of an API derivative (1.0 equiv.) and a corresponding alkene cross-metathesis partner (3 equiv.) dissolved in dry THF (0.8 mL) an appropriate amount of Ru complex **Ru3** or **Ru2** in dry THF (0.2 mL) was added. The resulting reaction mixture was stirred at room temperature for 4 hours.

2.7.1. Synthesis of estrone derivative (**29**)

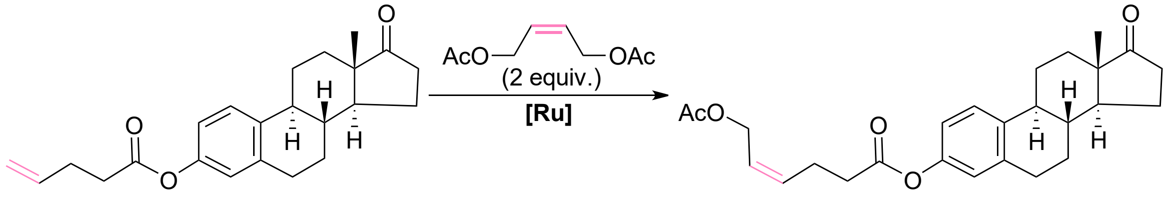

The reaction was carried out according to the general procedure with (*Z*)-1,4-diacetoxy-2-butene (109 mg, 0.6 mmol, 2.0 equiv.), *O*-(4-pentenoyl)estrone (71 mg, 0.2 mmol, 1.0 equiv.) and 10 mol% of **Ru3** or **Ru2**. The product was isolated by column chromatography (SiO_2, DCM to 5% MeOH/DCM).

Yield from the reaction catalysed with **Ru2** is 61% (***Z/E*** = 99:1).

Yield from the reaction catalysed with **Ru3** is 56% yield (***Z/E*** = 99:1).

^{1}H NMR (400 MHz, $CDCl_3$) δ 7.30 – 7.29 (m, 1H), 6.85 – 6.79 (m, 2H), 5.69 – 5.60 (m, 2H), 4.69 – 4.67 (m, 2H), 2.91 – 2.89 (m, 2H), 2.66 – 2.62 (m, 2H), 2.58 – 2.47 (m, 3H), 2.40 (dd, *J* = 11.3, 4.6 Hz, 1H), 2.32 – 2.25 (m, 1H), 2.19 – 1.94 (m, 6H), 1.68 – 1.39 (m, 6H), 0.91 (s, 3H).

^{13}C NMR (101 MHz, $CDCl_3$) δ 220.8, 171.5, 148.5, 138.0, 137.4, 132.4, 126.4, 125.3, 121.5, 118.7, 60.2, 50.4, 47.9, 44.1, 38.0, 35.9, 33.9, 31.5, 29.4, 26.3, 25.7, 23.0, 21.6, 21.0, 13.8.

The spectra correspond to those described in the literature.[20]

2.7.2. (*Z*)-4-((2R,3S)-3-((S)-1-((tert-butyldimethylsilyl)oxy)ethyl)-4-oxoazetidine-2-yl)but-2-en-1-yl acetate (**30**)

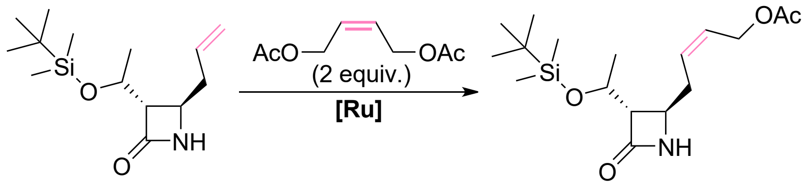

The reaction was carried out according to the general procedure with (*Z*)-1,4-diacetoxy-2-butene (109 mg, 0.6 mmol, 2.0 equiv.), (3*S*,4*R*)-3-[(1*R*)-1-(*tert*-butyldimethylsilyloxy)ethyl]-4-(prop-2-enyl)-2-azetidino-ne (54 mg, 0.2 mmol, 1.0 equiv.) and 10 mol% of **Ru3** or **Ru2**. The product was isolated by column chromatography (SiO_2, DCM to 3% MeOH/DCM) as a white solid.

Yield from the reaction catalysed with **Ru2** is 59% (***Z/E*** = 99:1).

Yield from the reaction catalysed with **Ru3** is 62% yield (***Z/E*** = 99:1).

^{1}H NMR (400 MHz, $CDCl_3$) δ 6.14 (s, 1H), 5.72 – 5.59 (m, 2H), 4.60 (d, *J* = 5.8 Hz, 2H), 4.21 – 4.09 (m, 1H), 3.68 (ddd, *J* = 7.2, 5.8, 2.2 Hz, 1H), 2.80 – 2.78 (m, 1H), 2.45 (qt, *J* = 13.8, 6.4 Hz, 2H), 2.04 (s, 3H), 1.20 (d, *J* = 6.2 Hz, 3H), 0.86 (s, 9H), 0.05 (d, *J* = 3.0 Hz, 6H).

^{13}C NMR (101 MHz, $CDCl_3$) δ 170.8, 168.4, 129.6, 126.7, 65.5, 63.8, 60.0, 50.2, 32.9, 25.7, 22.7, 20.9, 17.91, −4.3, −5.0.

HRMS (ESI) *m/z* Calcd. for $C_{17}H_{31}NO_4NaSi$ [M+Na]$^+$: 364.1920; Found: 364.1934.

EA: Calculated for $C_{17}H_{31}NO_4Si$: C, 59.79; H, 9.15; N, 4.10. Found: C, 59.75; H, 9.20; N, 4.11.

IR (cm^{-1}): 3272, 2954, 2929, 2856, 1739, 1379, 1372, 1227, 1142, 1097, 1027, 831, 775.

2.7.3. Synthesis of (*Z*)-(1-(14-hydroxytetradec-8-en-1-yl)-1*H*-indol-3-yl) (2,2,3,3-tetramethylcyclopropyl)methanone (**31**)

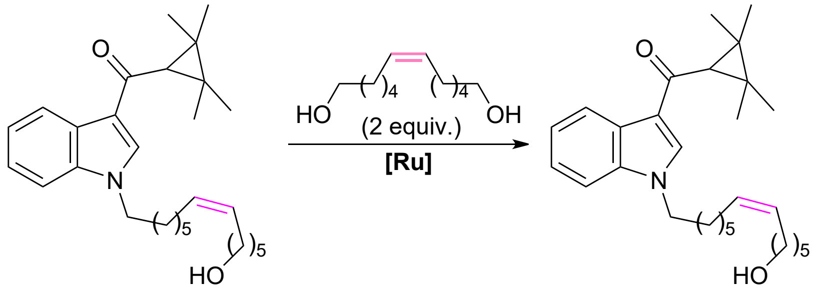

The reaction was carried out according to the general procedure with (*Z*)-dodec-6-ene-1,12-diol (120 mg, 0.6 mmol, 2.0 equiv.), (1-(oct-7-en-1-yl)-1*H*-indol-3-yl)(2,2,3,3-tetramethyl cyclopropyl)methanone (70.3 mg, 0.2 mmol, 1.0 equiv.) and 10 mol% of **Ru3** or **Ru2**. The product was isolated by column chromatography (SiO_2, hexane to 50% EtOAc/hexane) as a white solid.

Yield from the reaction catalysed with **Ru2** is 62% (***Z/E*** = 99:1).

Yield from the reaction catalysed with **Ru3** is 68% yield (***Z/E*** = 99:1).

^{1}H NMR (400 MHz, $CDCl_3$) δ 8.45 – 8.30 (m, 1H), 7.66 (s, 1H), 7.36 – 7.32 (m, 1H), 7.30 – 7.23 (m, 1H), 5.47 – 5.20 (m, 2H), 4.15 (t, *J* = 7.2 Hz, 2H), 3.63 (t, *J* = 6.6 Hz, 2H), 2.06 – 1.83 (m, 7H), 1.56 (s, 3H), 1.44 – 1.25 (m, 23H).

^{13}C NMR (101 MHz, $CDCl_3$) δ 194.7, 136.6, 133.5, 129.9, 129.7, 126.4, 122.9, 122.7, 122.1, 119.6, 109.6, 77.2, 63.0, 47.0, 41.7, 32.7, 31.6, 29.9, 29.5 (d, *J* = 1.7 Hz), 28.8, 27.1, 27.0, 26.8, 25.4, 24.1, 17.0.

HRMS (ESI) *m/z* Calcd. for. $H_{29}H_{44}NO_2$ [M+H$^+$]: 438.3372; Found: 438.3376

IR (cm^{-1}): 3121, 3037, 2928, 2854, 1729, 1580, 1137, 728.

2.7.4. Synthesis of Sildenafil derivative (**32**)

Synthesis of (*Z*)-*N*-benzylnon-6-en-1-amine

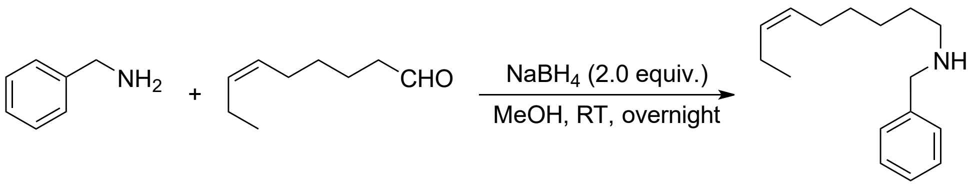

A flask was charged with benzylamine (2.0 g, 2.0 mL, 1.0 equiv.), dry MeOH (20 mL), and (*Z*)-6-nonenal (3.0 g, 3.6 mL, 20.1 mmol, 1.1 equiv.). The reaction mixture was stirred for 3 h at RT (until the full consumption of benzylamine determined by GC) and sodium borohydride (1.4 g, 36.6 mmol, 2.0 equiv.) was added portion wise to the reaction mixture cooled with a water/ice cooling bath. After addition of sodium borohydride, the reaction was stirred at RT overnight. Next, the reaction mixture was quenched with a NaOH solution (10% w/v, 20 mL) and transferred to a separatory funnel. The mixture was extracted with DCM (5 × 25 mL), combined organic layers were dried over sodium sulphate, evaporated to dryness, and purified using column chromatography (alumina basic, Brockmann III, 1% Et_3N in *n*-hexane to 5% EtOAc + 1% Et_3N in *n*-hexane) to give the product as a colourless liquid (3.8 g, 16.4 mmol, 90% yield).

^{1}H NMR (400 MHz, $CDCl_3$) δ 7.4 – 7.3 (m, 4H), 7.3 – 7.2 (m, 1H), 5.6 – 5.1 (m, 2H), 3.8 (s, 2H), 2.6 – 2.6 (m, 2H), 2.3 – 1.8 (m, 4H), 1.8 – 1.5 (m, 2H), 1.5 – 1.2 (m, 4H), 1.0 (t, *J* = 7.5 Hz, 3H).

^{13}C NMR (101 MHz, $CDCl_3$) δ 140.6, 131.7, 129.1, 128.4, 128.1, 126.8, 54.1, 49.5, 30.0, 29.7, 27.0, 27.0, 20.5, 14.4.

HRMS (APCl TOF *m*/*z*) calculated for $C_{16}H_{26}NO$ $[M+H]^+$: 232.2065, Found: 232.2068.

EA: calculated for $C_{16}H_{25}N$: C, 83.06; H, 10.89; Found C, 83.07; H, 11.02.

IR (cm^{-1}): 3085, 3063, 3026, 3004, 2961, 2927, 2854, 2811, 1495, 1453, 1362, 1119, 1070, 1028, 730, 696, 593.

Synthesis of (*Z*)-*N*-benzyl-4-ethoxy-3-(1-methyl-7-oxo-3-propyl-6,7-dihydro-1*H*-pyrazolo[4,3-d]pyrimidin-5-yl)-*N*-(non-6-en-1-yl)benzene-sulfonamide

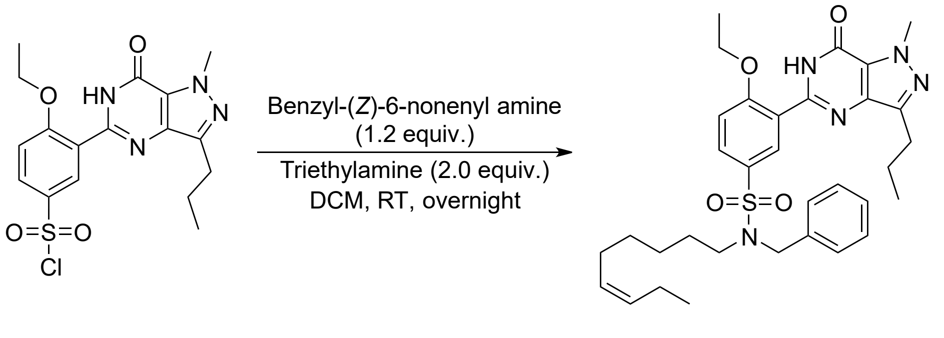

The flask was charged with 5-(5-chlorosulphonyl-2-ethoxyphenyl)-1-methyl-3-*N*-propyl-1,6-dihydro-7*H*-pyrazolo[4,3-d]pyrimidin-7-one (1.53 g, 3.6 mmol, 1.0 equiv.) and DCM (10 mL). Then to this mixture, a solution of (*Z*)-*N*-benzyldodec-9-en-1-amine (1.0 g, 4.3 mmol, 1.2 equiv.) and triethylamine (5.23 g, 1 mL, 7.2 mmol, 2.0 equiv.) in DCM (10 mL) was added dropwise. The reaction mixture was stirred overnight, evaporated to dryness, and purified using column chromatography (SiO_2, MeOH/DCM from 0 to 10%) to give product as a colourless powder (1.7 g, 2.8 mmol, 78% yield).

^{1}H NMR (400 MHz, $CDCl_3$) δ 10.87 (s, 1H), 8.91 (dd, *J* = 2.5, 1.1 Hz, 1H), 7.90 (ddd, *J* = 8.7, 2.5, 0.9 Hz, 1H), 7.35 – 7.21 (m, 5H), 7.12 (d, *J* = 8.8 Hz, 1H), 5.42 – 5.10 (m, 2H), 4.45 – 4.31 (m, 4H), 4.27 (d, *J* = 0.9 Hz, 3H), 3.25 – 3.07 (m, 2H), 3.02 – 2.83 (m, 2H), 1.99 – 1.76 (m, 6H), 1.64 (td, *J* = 7.0, 0.9 Hz, 3H), 1.38 (dq, *J* = 14.8, 7.1 Hz, 2H), 1.22 – 1.07 (m, 4H), 1.00 (td, *J* = 7.4, 0.7 Hz, 3H), 0.95 – 0.85 (m, 3H).

^{13}C NMR (101 MHz, $CDCl_3$) δ 158.9, 153.6, 146.9, 146.5, 138.4, 136.3, 133.6, 131.8, 131.1, 130.4, 128.7, 128.6, 128.2, 127.8, 124.5, 121.0, 113.0, 66.0, 51.9, 48.2, 38.2, 29.1, 27.9, 27.7, 26.8, 26.2, 22.3, 20.4, 14.6, 14.3, 14.0.

HRMS (APCl TOF *m/z*) calculated for $C_{33}H_{44}N_5O_4S$ $[M+H]^+$: 606.3114, Found: 606.3116.

EA: calculated for $C_{33}H_{43}N_5O_4S$: C, 65.21; H, 7.46; N,11.56; Found C, 65.46; H, 7.13; N, 11.42.

IR (cm^{-1}): 3284, 2960, 2931, 2871, 1698, 1601, 1580, 1561, 1536, 1486, 1466, 1455, 1393, 1345, 1271, 1245, 1159, 1125, 1102, 1077, 1026, 930, 886, 810, 778, 767, 747, 729, 695, 653, 606, 587, 577, 562, 517.

Cross metathesis between (*Z*)-*N*-benzyl-4-ethoxy-3-(1-methyl-7-oxo-3-propyl-6,7-dihydro-1*H*-pyrazolo[4,3-d]pyrimidin-5-yl)-*N*-(non-6-en-1-yl)benzene-sulfonamide and (*Z*)-1,4-diacetoxy-2-butene

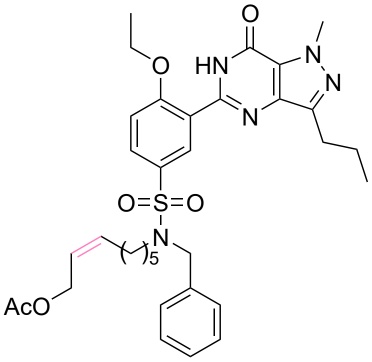

The reaction was carried out according to the general procedure with (*Z*)-1,4-diacetoxy-2-butene (109 mg, 0.6 mmol, 2.0 equiv.), Sildenafil derivative (121 mg, 0.2 mmol, 1.0 equiv.) and 0.5 mol% of **Ru3** or **Ru2**. The product was isolated by column chromatography (SiO_2, hexane to 40% EtOAc/hexane) as a white solid. Yield from the reaction catalysed with **Ru2** is 76% (***Z/E*** = 99:1). Yield from the reaction catalysed with **Ru3** is 74% yield (***Z/E*** = 99:1).

^{1}H NMR (400 MHz, $CDCl_3$) δ 10.87 (s, 1H), 8.90 (d, *J* = 2.5 Hz, 1H), 7.90 (dd, *J* = 8.8, 2.5 Hz, 1H), 7.33 – 7.28 (m, 4H), 7.13 (d, *J* = 8.8 Hz, 1H), 5.59 – 5.37 (m, 2H), 4.55 – 4.51 (m, 2H), 4.40 – 4.34 (m, 4H), 4.27 (s, 3H), 3.16 – 3.12 (m, 2H), 2.97 – 2.87 (m, 2H), 2.03 (s, 3H), 1.94 (q, *J* = 6.9 Hz, 2H), 1.89 – 1.81 (m, 2H), 1.64 (t, *J* = 6.9 Hz, 3H), 1.43 – 1.31 (m, 2H), 1.23 – 1.08 (m, 4H), 0.99 (t, *J* = 6.9 Hz, 3H).

^{13}C NMR (101 MHz, $CDCl_3$) δ 171.0, 158.9, 153.6, 146.9, 146.5, 138.4, 136.3, 134.8, 133.5, 131.1, 130.4, 128.6, 128.3, 127.8, 123.5, 121.0, 113.0, 66.0, 60.3, 52.1, 48.2, 38.2, 28.8, 27.9, 27.7, 27.2, 26.2, 22.3, 21.0, 14.6, 14.0.

HRMS (ESI) *m/z* Calcd. for $C_{34}H_{44}N_5O_6S$ [M+H$^+$]: 650.3012; Found: 650.3022.

EA Calcd. for $C_{34}H_{43}N_5O_6S$: C, 62.85; H, 6.67; N, 10.78; Found: C, 62.74; H, 6.65; N, 10.77.

IR (cm^{-1}): 3304, 2931, 2870, 1738, 1695, 1600, 1582, 1488, 1454, 1339, 1229, 1152, 1023, 929, 816, 651, 587.

2.7.5. Synthesis of (*Z*)-9-hydroxynon-3-en-1-yl 3-(4-(7*H*-pyrrolo[2,3-d]pyrimidin-4-yl)-1*H*-pyrazol-1-yl)-1-(ethylsulfonyl)azetidine-3-carboxylate (**33**)

Synthesis of (*Z*)-hex-3-en-1-yl-2-(3-(4-(7*H*-pyrrolo[2,3-d]pyrimidin-4-yl)-1*H*-pyrazol-1-yl)-1-(ethylsulfonyl)azetidine-3-yl)acetate

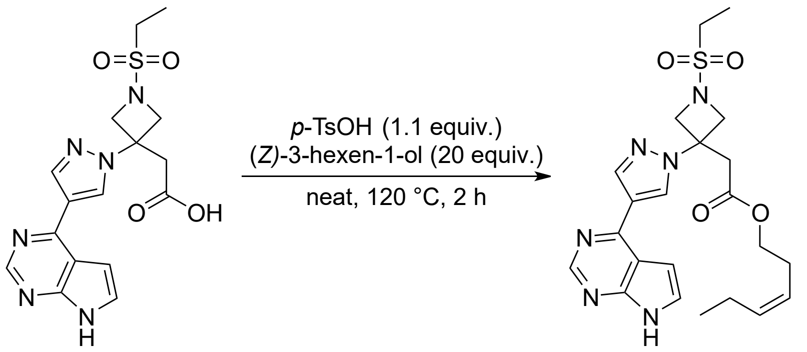

A flask was charged with 2-(3-(4-(7*H*-pyrrolo[2,3-d]pyrimidin-4-yl)-1*H*-pyrazol-1-yl)-1-(ethylsulfonyl)azetidin-3-yl)acetic acid (750 mg, 1.9 mmol, 1.0 equiv.), *p*-TsOH (404 mg, 2.1 mmol, 1.1 equiv.) and (*Z*)-3-hexenol (3.87 g, 4.6 mL, 38.6 mmol, 20 equiv.). A vial was heated to 120 °C for 2 h. Then, from reaction mixture an excess of alcohol was evaporated (0.1 mbar, 45 °C). The residue was dissolved in DCM (20 mL) and extracted with a saturated solution of potassium carbonate (20 mL). Water layer was extracted with DCM (2 × 20 mL). Combined organic layers were dried over sodium sulphate, the solvent was evaporated, and the residue was crystallised from EtOH/Et_2O to give product as colourless solid (460 mg, 0.97 mmol, 49% yield).

^{1}H NMR (400 MHz, $CDCl_3$) δ 11.49 (s, 1H), 8.87 (s, 1H), 8.51 (s, 1H), 8.31 (s, 1H), 7.45 (dd, *J* = 3.7, 2.1 Hz, 1H), 6.78 (dd, *J* = 3.7, 1.7 Hz, 1H), 5.42 (dtt, *J* = 10.7, 7.3, 1.6 Hz, 1H), 5.18 (dtt, *J* = 10.7, 7.3, 1.7 Hz, 1H), 4.57 (d, *J* = 9.2 Hz, 2H), 4.39 (d, *J* = 9.2 Hz, 2H), 4.03 (t, *J* = 6.9 Hz, 2H), 3.36 (s, 2H), 3.05 (q, *J* = 7.4 Hz, 2H), 2.28 (qd, *J* = 7.1, 1.6 Hz, 2H), 1.95 (pd, *J* = 7.5, 1.6 Hz, 2H), 1.39 (t, *J* = 7.4 Hz, 3H), 0.89 (t, *J* = 7.5 Hz, 3H).

^{13}C NMR (101 MHz, $CDCl_3$) δ 169.1, 152.4, 151.1, 150.8, 140.0, 134.8, 128.9, 125.8, 123.1, 122.3, 114.2, 100.4, 64.7, 60.0, 56.9, 46.3, 41.8, 26.4, 20.5, 14.1, 7.9.

HRMS (ESI TOF *m/z*) calculated for $C_{22}H_{29}N_6O_4S$ $[M+H]^+$: 473.1966, Found: 473.1964.

EA: calculated for $C_{22}H_{28}N_6O_4S$: C, 55.92; H, 5.97; N, 17.78, Found C, 55.85; H, 5.72; N, 17.67.

IR (cm^{-1}): 3199, 3135, 2998, 2962, 2933, 2860, 1724, 1579, 1506, 1459, 1449, 1387, 1358, 1346, 1320, 1189, 1141, 1077, 1022, 1006, 976, 930, 900, 877, 829, 775, 746, 737, 606, 597.

Cross metathesis between (*Z*)-hex-3-en-1-yl-2-(3-(4-(7*H*-pyrrolo[2,3-d]pyrimidin-4-yl)-1*H*-pyrazol-1-yl)-1-(ethylsulfonyl)azetidine-3-yl)acetate and (*Z*)-dodec-6-ene-1,12-diol

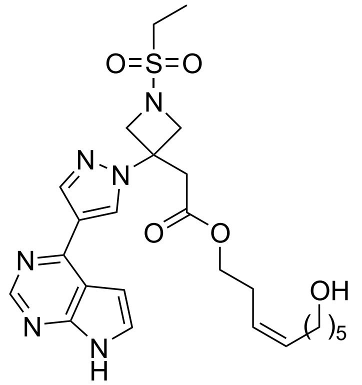

The reaction was carried out according to the general procedure with (*Z*)-dodec-6-ene-1,12-diol (52.4 mg, 0.26 mmol, 2.0 equiv.), (*Z*)-hex-3-en-1-yl-2-(3-(4-(7H-pyrrolo[2,3-d]pyrimidin-4-yl)-1H-pyrazol-1-yl)-1-(ethylsulfonyl)azetidine-3-yl)acetate (Baricitinib analogue) (40 mg, 0.0872 mmol, 1.0 equiv.), and 0.5 mol% of **Ru3** or **Ru2**. The product was isolated by column (SiO_2, DCM to 7% MeOH/DCM) as a white solid.

Yield from the reaction catalysed with **Ru2** is 82% (***Z/E*** = 88:12).
Yield from the reaction catalysed with **Ru3** is 85% yield (***Z/E*** = 99:1).

^{1}H NMR (400 MHz, $CDCl_3$) δ 10.46 (s, 1H), 8.85 (s, 1H), 8.51 (s, 1H), 8.30 (d, *J* = 8.2 Hz, 1H), 7.46 – 7.38 (m, 1H), 6.79 (dd, *J* = 3.7, 1.6 Hz, 1H), 5.46 – 5.33 (m, 1H), 5.26 – 5.15 (m, 1H), 4.57 (d, *J* = 9.2 Hz, 2H), 4.40 (d, *J* = 9.1 Hz, 2H), 4.03 (t, *J* = 6.9 Hz, 2H), 3.62 (t, *J* = 6.5 Hz, 2H), 3.35 (s, 2H), 3.06 (q, *J* = 7.4 Hz, 2H), 2.32 – 2.21 (m, 2H), 1.93 (d, *J* = 1.6 Hz, 2H), 1.57 – 1.47 (m, 2H), 1.39 (t, *J* = 7.4 Hz, 4H), 1.33 – 1.23 (m, 4H).

^{13}C NMR (101 MHz, $CDCl_3$) δ 169.1, 152.3, 151.3, 150.8, 140.0, 133.0, 128.9, 125.7, 124.0, 122.2, 114.1, 100.5, 64.7, 62.7, 60.0, 57.0, 46.3, 41.9, 32.6, 29.7, 29.2, 27.1, 26.6, 25.3, 7.9.

LRMS (APCl TOF *m/z*) calculated for $C_{25}H_{35}N_6O_5S$ $[M+H]^+$: 531.24, Found: 531.24

EA Calcd. For. $C_{24}H_{32}N_6O_5S$: C, 55.80; H, 6.24. Found: C, 55.26; H, 6.09.

IR (cm^{-1}): 3106, 2925, 2855, 1623, 1523, 1464, 1390, 1206, 741.

2.8. Pheromones or pheromone precursors obtained in the CM reaction

2.8.1. Synthesis of methyl (*Z*)-tetradec-9-enoate (**40**)

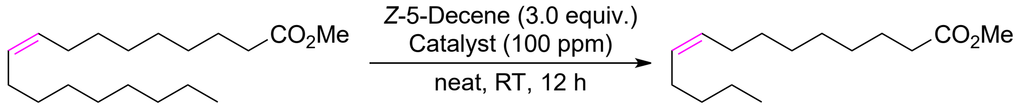

A Schlenk flask was charged with tetradecane (2.36 g, 11.8 mmol, 0.5 equiv.) methyl oleate (6.98 g, 23.6 mmol, 1.0 equiv.) and (*Z*)-5-decene (9.91 g, 70.7 mmol, 3.0 equiv.). Then, a corresponding catalyst was added (100 ppm, 2.0 mg of **Ru2**, 1.96 mg of **Ru3**) as a solid and the reaction mixture was stirred at room temperature for 12 h. An aliquot of the crude mixture was analysed by GC to determine the conversion, and the ester was isolated by flash column chromatography (SiO_2, *n*-hexane up to 1.5% EtOAc in *n*-hexane). The combined ester fractions were collected and distilled using the Hickmann adaptor (10^{-3} mbar, 90 °C) to obtain a pure product as a colourless liquid.

Yield from the reaction catalysed with **Ru2** is 66% (***Z/E*** = 99:1).

Yield from the reaction catalysed with **Ru3** is 62% (***Z/E*** = 99:1).

^{1}H NMR (400 MHz, $CDCl_3$) δ 5.89 – 5.12 (m, 2H), 3.63 (s, 3H), 2.27 (t, J = 7.6 Hz, 2H), 2.14 – 1.80 (m, 4H), 1.76 – 1.48 (m, 2H), 1.44 – 1.14 (m, 12H), 1.01 – 0.70 (m, 3H).

^{13}C NMR (101 MHz, $CDCl_3$) δ 174.2, 129.9, 129.7, 51.3, 34.0, 31.9, 29.6, 29.1, 29.1, 29.0, 27.1, 26.9, 24.9, 22.3, 13.9.

The spectra correspond to those described in the literature.[22]

2.8.2. Synthesis of (Z)-octadec-9-en-1-yl acetate (**41**)

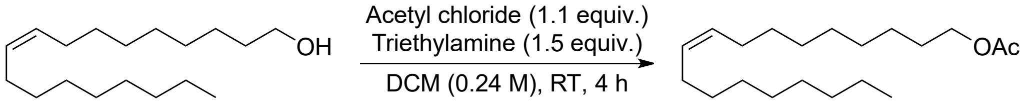

A 250 mL round-bottom flask was charged with oleyl alcohol (9.8 g, 36.5 mmol, 1.0 equiv.), triethylamine (5.52 g, 7.6 mL, 54.8 mmol. 1.5 equiv.) and DCM (150 mL). Then, to the mixture a solution of acetyl chloride (3.19 g, 2.9 mL, 40.2 mmol, 1.1 equiv.) in DCM (15 mL) was added dropwise. The reaction mixture was stirred until full consumption of the substrate (*ca.* 4 h). The reaction mixture was transferred to a separation funnel, the organic layer was washed with water (50 mL) and a solution of potassium carbonate (20% w/v, 50 mL). Organic layer was dried over sodium sulphate, evaporated to dryness, and distilled with Kugelrohr (10^{-3} mbar, 180 °C).

Distillate was passed through a bed of activated alumina (neutral, Brockman I) to obtain a colourless liquid (10.2 g, 32.8 mmol, 90% yield).

^{1}H NMR (400 MHz, CD_2Cl_2) δ 5.53 – 4.89 (m, 2H), 4.04 (t, *J* = 6.8 Hz, 2H), 2.04 (s, 3H), 2.03 – 1.97 (m, 4H), 1.74 – 1.49 (m, 2H), 1.38 – 1.11 (m, 22H), 0.89 – 0.85 (m, 3H).

^{13}C NMR (101 MHz, CD_2Cl_2) δ 169.3, 128.0, 127.8, 62.7, 30.0, 27.8, 27.8, 27.6, 27.5, 27.4, 27.3, 27.3, 26.7, 25.3, 25.2, 24.0, 20.8, 19.1, 12.2.

The spectra correspond to those described in the literature.[23]

2.8.3. Synthesis of (*Z*)-dodec-9-en-1-yl acetate (**42**)

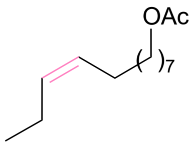

A 4 mL vial was charged with oleyl acetate (500 mg, 1.45 mmol, 1.0 equiv.), (*Z*)-3-hexene (393 mg, 0.578 mL, 4.35 mmol, 3.0 equiv.) and dry THF (7.2 mL). Then, an appropriate amount (1 mol%) of a corresponding catalyst (**Ru2** or **Ru3**) was added to the vial. The reaction mixture was stirred at room temperature overnight, concentrated *in vacuo*, and purified by column chromatography (SiO_2, *n*-hexane to 5% EtOAc/*n*-hexane). The product fractions were collected, concentrated under reduced pressure and distilled using the Hickmann adapter (10^{-3} mbar, 85 °C) to provide the desired product as a colourless oil.

Yield from the reaction catalysed with **Ru2** is 49% (***Z/E*** = 92:8).

Yield from the reaction catalysed with **Ru3** is 52% yield (***Z/E*** = 96:4).

^{1}H NMR (400 MHz, $CDCl_3$) δ 5.43 – 5.25 (m, 2H), 4.04 (t, *J* = 6.8 Hz, 2H), 2.09 – 1.91 (m, 7H), 1.61 (dt, *J* = 13.9, 6.8 Hz, 2H), 1.39 – 1.26 (m, 10H), 0.95 (t, *J* = 7.5 Hz, 3H).

^{13}C NMR (101 MHz, $CDCl_3$) δ 171.3, 131.6, 129.2, 64.6, 32.5, 29.7, 29.6, 29.4, 29.2, 29.2, 29.0, 28.6, 27.0, 25.9, 21.0, 20.5, 14.4.

The spectra correspond to those described in the literature.[24]

2.8.4. Synthesis of oct-7-en-1-yl acetate

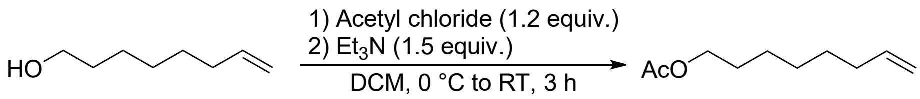

A 250 mL flask was charged with dry DCM (150 mL), solvent was cooled down to 0 °C in an ice/water bath. 7-octen-1-ol (10 g, 11.8 mL, 75.7 mmol, 1 equiv.) and triethylamine (11.5 g, 15.8 mL, 113 mmol, 1.5 equiv.) were added followed by dropwise addition of acetyl chloride (7.2 g, 6.52 mL, 90.8 mmol, 1.2 equiv.). The reaction mixture was warmed up to RT, stirred for 3 h, and transferred to a separatory funnel. The mixture was washed with water (50 mL), 5%

solution of NaOH (50 mL), and dried over Na_2SO_4. After removing of drying agent, solvent was evaporated and the residue was distilled *via* Kugelrohr (80 °C, 0.01 mbar) to give product as a colourless oil (11.62 g, 68.2 mmol, 90%).

^{1}H NMR (400 MHz, $CDCl_3$) δ 5.79 (ddt, *J* = 16.9, 10.2, 6.6 Hz, 1H), 5.22 – 4.78 (m, 2H), 4.04 (t, *J* = 6.7 Hz, 2H), 2.29 – 1.83 (m, 5H), 1.49 – 1.21 (m, 6H).

^{13}C NMR (101 MHz, $CDCl_3$) δ 171.1, 138.9, 114.3, 64.5, 33.6, 28.7, 28.7, 28.5, 25.7, 21.0.

The spectra correspond to those described in the literature.[25]

2.8.5. Synthesis of (7*E*,9*Z*)-dodeca-7,9-dien-1-yl acetate (**44**)

2.8.6. Synthesis of (7*E*,9*Z*)-dodeca-7,9-dien-1-yl acetate (**44**) using the Wittig reaction (as a product standard for preparation of the calibration curve)

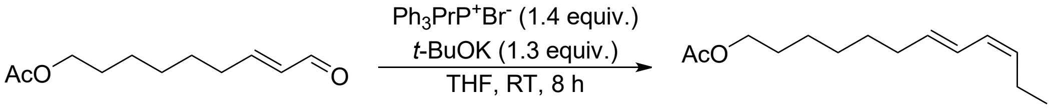

The 250 mL flask was charged with phosphonium salt (8.43 g, 21.9 mmol, 1.4 equiv.), *t*-BuOK (2.33 g, 20.3 mmol, 1.3 equiv.) and dry THF (150 mL). The suspension was stirred for 30 min. and the solution of (*E*)-9-oxonon-7-en-1-yl acetate (3.1 g, 15.6 mmol, 1.0 equiv.) in THF (20 mL) was added dropwise. The reaction mixture was stirred at room temperature for 8 h, the solids were filtered off, the solution was transferred to a separation funnel followed by addition of water (50 mL). The organic layers were collected, and water phase was extracted with DCM (2 × 50 mL). The combined organic layers were dried over sodium sulphate and evaporated to dryness. The residue was suspended in *n*-hexane (200 mL) and sonicated for 30 min. The solid precipitate was filtered off, the solution was concentrated and purified by flash column chromatography (SiO_2, *n*-hexane to 10% of EtOAc in *n*-hexane) to obtain the desired product as a colourless liquid (2.8 g, 12.5 mmol, 80% of yield). The product was obtained as 7*E*,9*Z*/7*E*,9*E* mixture with a ratio *Z*/*E* = 85/15 for newly formed double bond (by ^{1}H NMR).

2.8.7. Synthesis of (7*E*,9*Z*)-dodeca-7,9-dien-1-yl acetate (**44**) in CM reaction

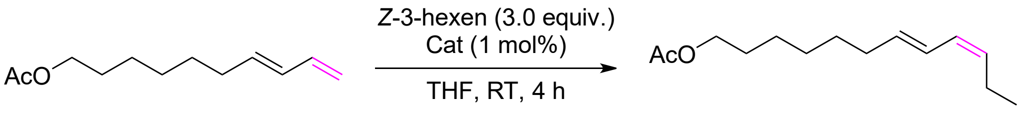

A 4 mL vials was charged with (*E*)-deca-7,9-dien-1-yl acetate (39.3 mg, 0.2 mmol, 1.0 equiv.), (*Z*)-3-hexene (52.1 mg, 0.578 mL, 0.6 mmol, 3.0 equiv.), tetradecane (20 mg, 0.1 mmol, 0.5 equiv.), and dry THF (0.5 mL). Then the catalyst (1 mol%, 1.70 mg of **Ru2** and 1.66 mg of **Ru3** as solution in 0.5 mL of THF) was added. Reactions were stirred for 4 h at RT and the samples were analysed by GC

Yield from the reaction catalysed with **Ru2** is 52% (***Z/E*** = 99:1) by **GC**.

Yield from the reaction catalysed with **Ru3** is 68% yield (***Z/E*** = 99:1) by **GC**.

^{1}H NMR (400 MHz, $CDCl_3$) δ 6.29 (ddq, *J* = 15.2, 11.0, 1.4 Hz, 1H), 6.10 – 5.94 (m, 0.15H), 5.95 – 5.86 (m, 0.85H), 5.64 (dt, *J* = 14.6, 7.0 Hz, 0.85H), 5.59 – 5.45 (m, 0.15H), 5.30 (dt, *J* = 10.8, 7.5 Hz, 1H), 4.04 (t, *J* = 6.7 Hz, 2H), 2.17 (pd, *J* = 7.6, 1.6 Hz, 2H), 2.09 (q, *J* = 6.8 Hz, 2H), 2.04 (s, 3H), 1.61 (p, *J* = 6.8 Hz, 2H), 1.49 – 1.28 (m, 6H), 0.99 (t, *J* = 7.5 Hz, 3H).

^{13}C NMR (101 MHz, $CDCl_3$) δ 171.2, 134.4 (**major**), 134.0 (**minor**), 132.1 (**minor**), 131.8 (**major**), 130.5 (**minor**), 129.3(**minor**), 127.9(**major**), 125.6 (**major**), 64.6, 32.7 (**major**), 32.5(**minor**), 29.3 (**minor**), 29.2 (**major**), 28.8 (**major**), 28.8 (**minor**), 28.5, 25.8 (**major**), 25.6 (**minor**), 21.0, 21.0, 14.3 (**major**), 13.6 (**minor**).

The spectra correspond to those described in the literature.[26]

2.9. Reproduction of NMR spectra

2.9.1. Compound for catalysts synthesis

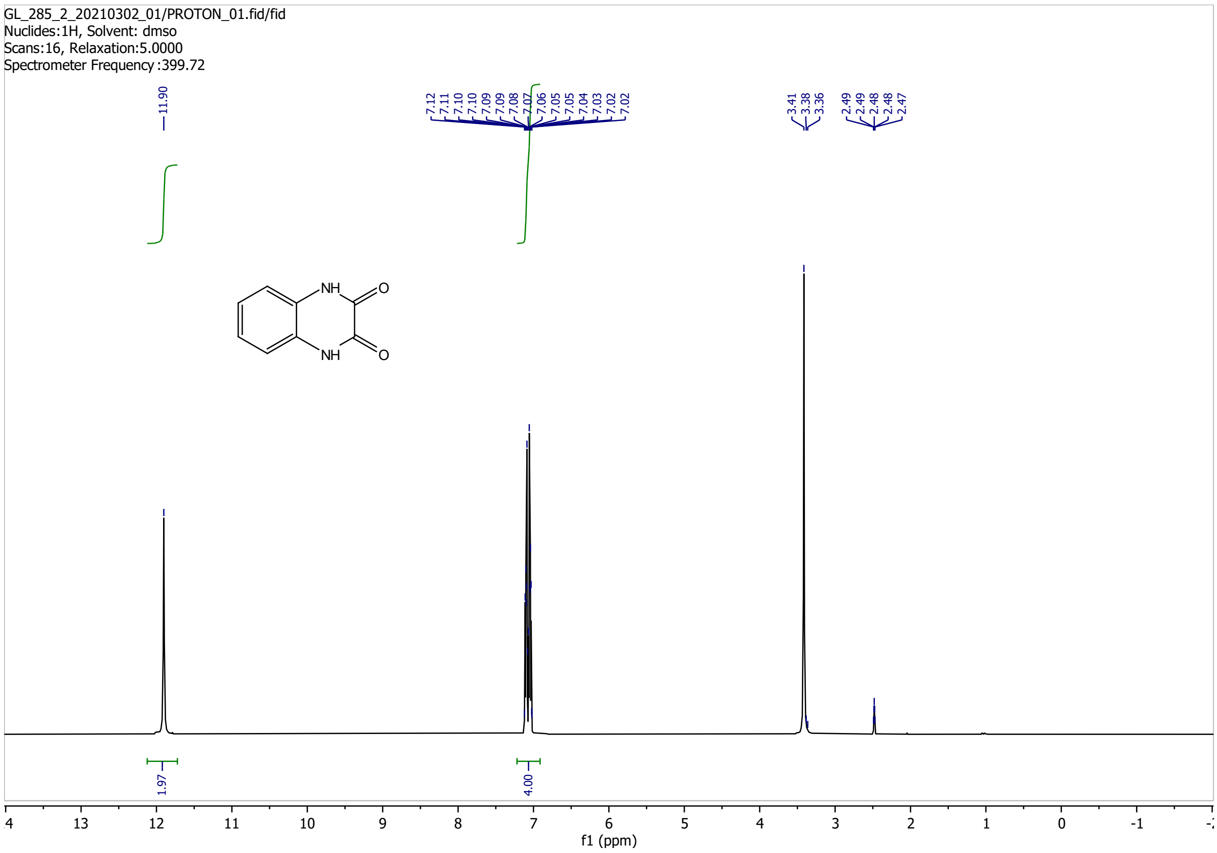

Figure S8. 1H NMR (400 MHz, DMSO-*d6*) of 1,4-dihydroquinoxaline-2,3-dione (4)

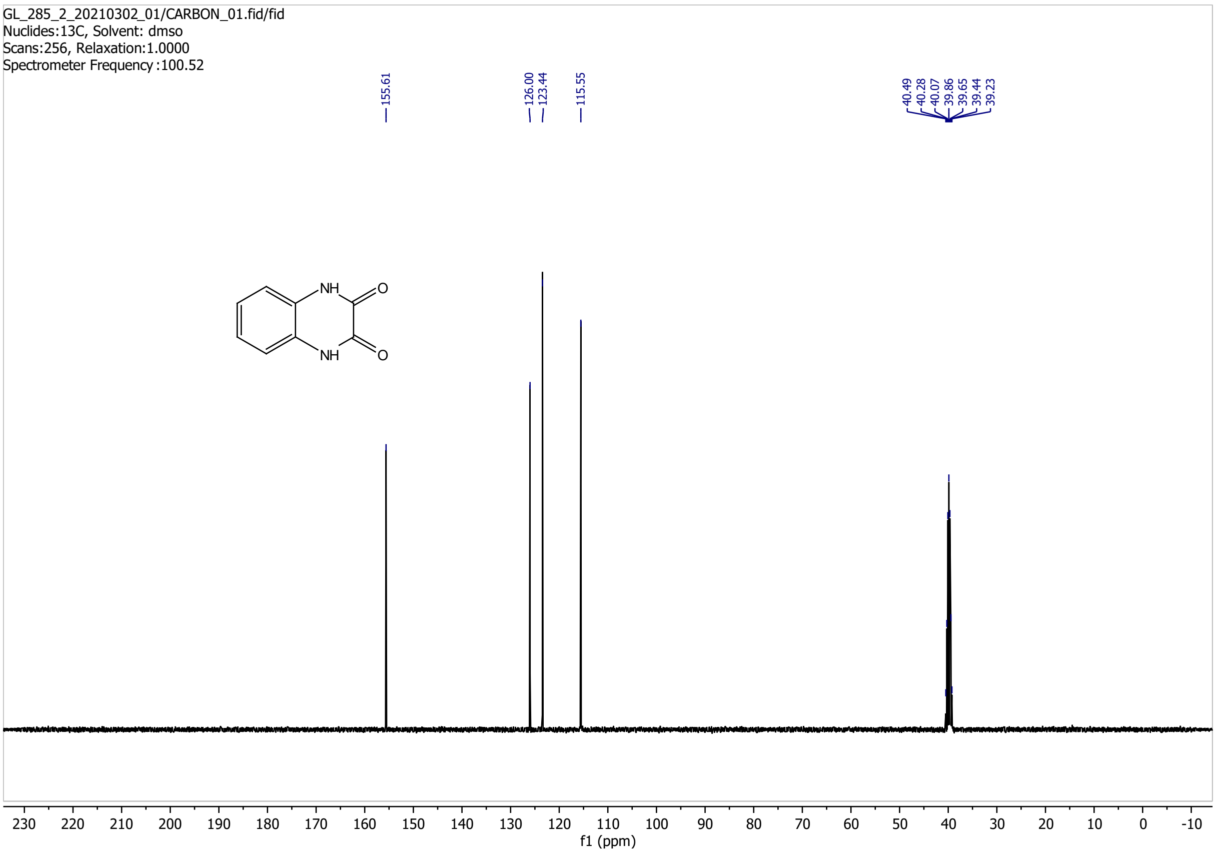

Figure S9. ^{13}C NMR (101 MHz, DMSO-d_6) of 1,4-dihydroquinoxaline-2,3-dione (4)

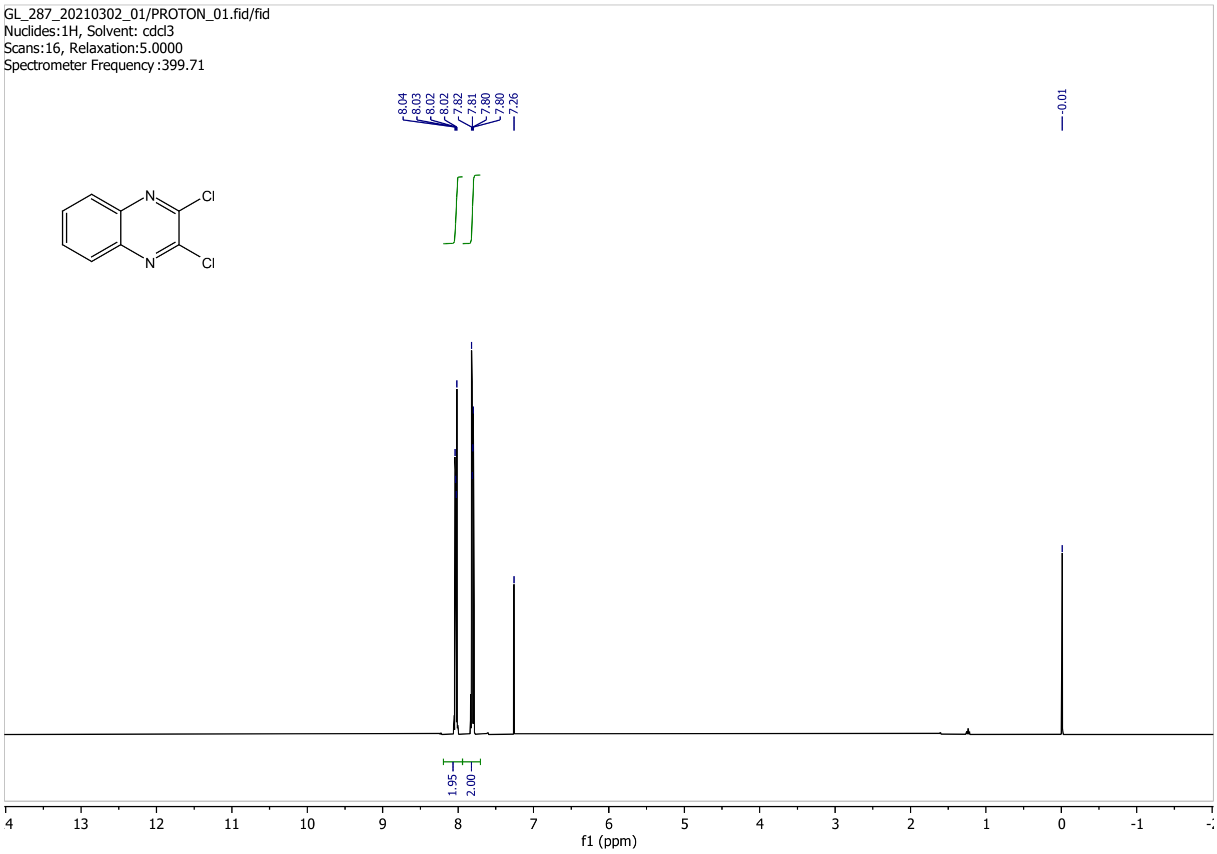

Figure S10. ^{1}H NMR (400 MHz, CDCl3) of 2,3-dichloroquinoxaline (4)

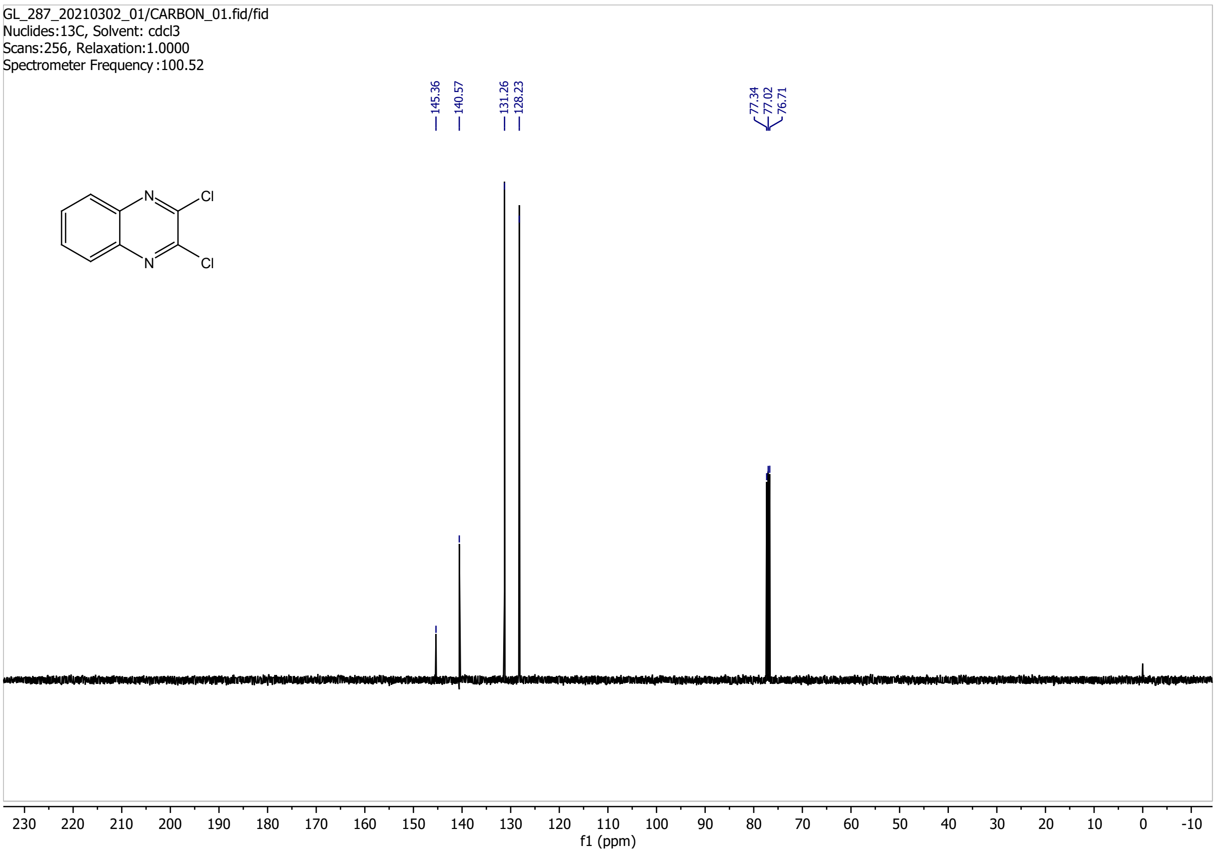

Figure S11. ^{13}C NMR (101 MHz, CDCl3) of 2,3-dichloroquinoxaline (6)

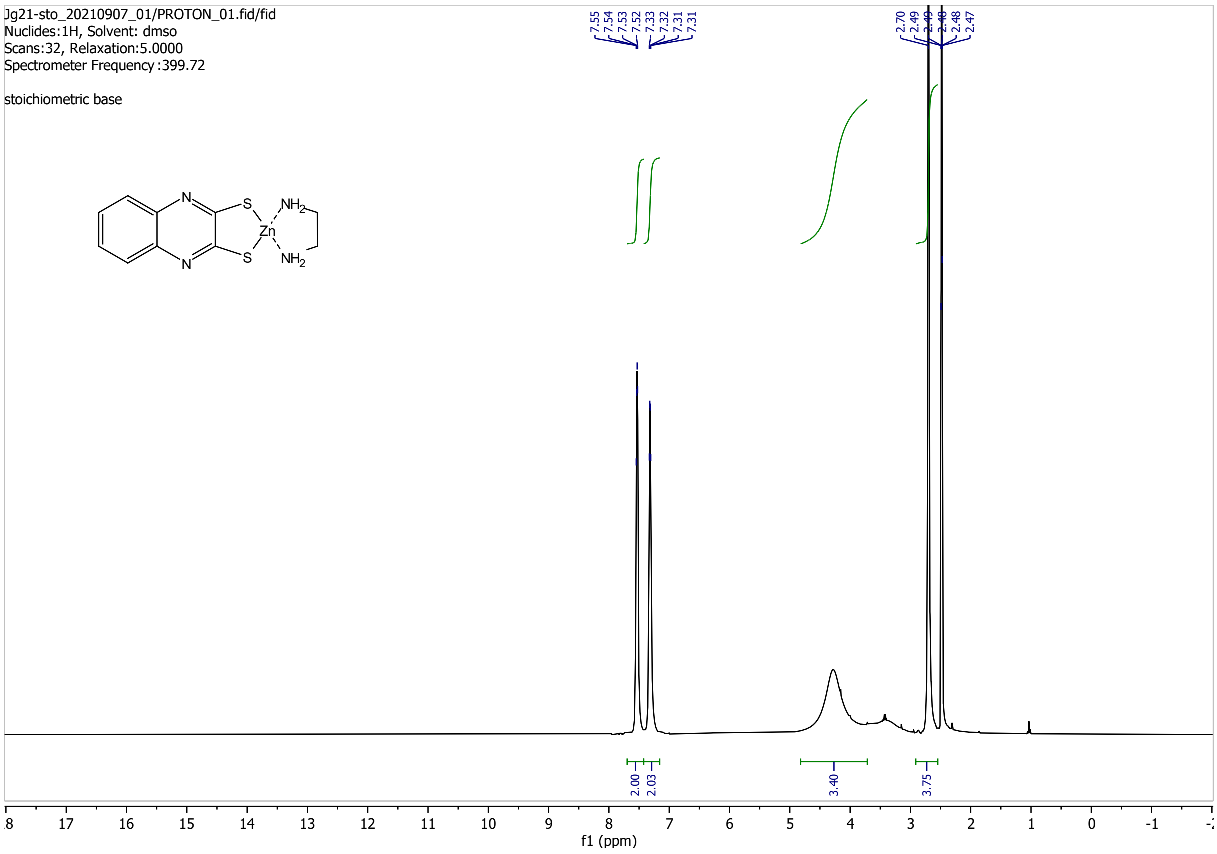

Figure S12. ^{1}H NMR (400 MHz, DMSO-d_6) of dithioquinoxaline-diamine zinc complex (8)

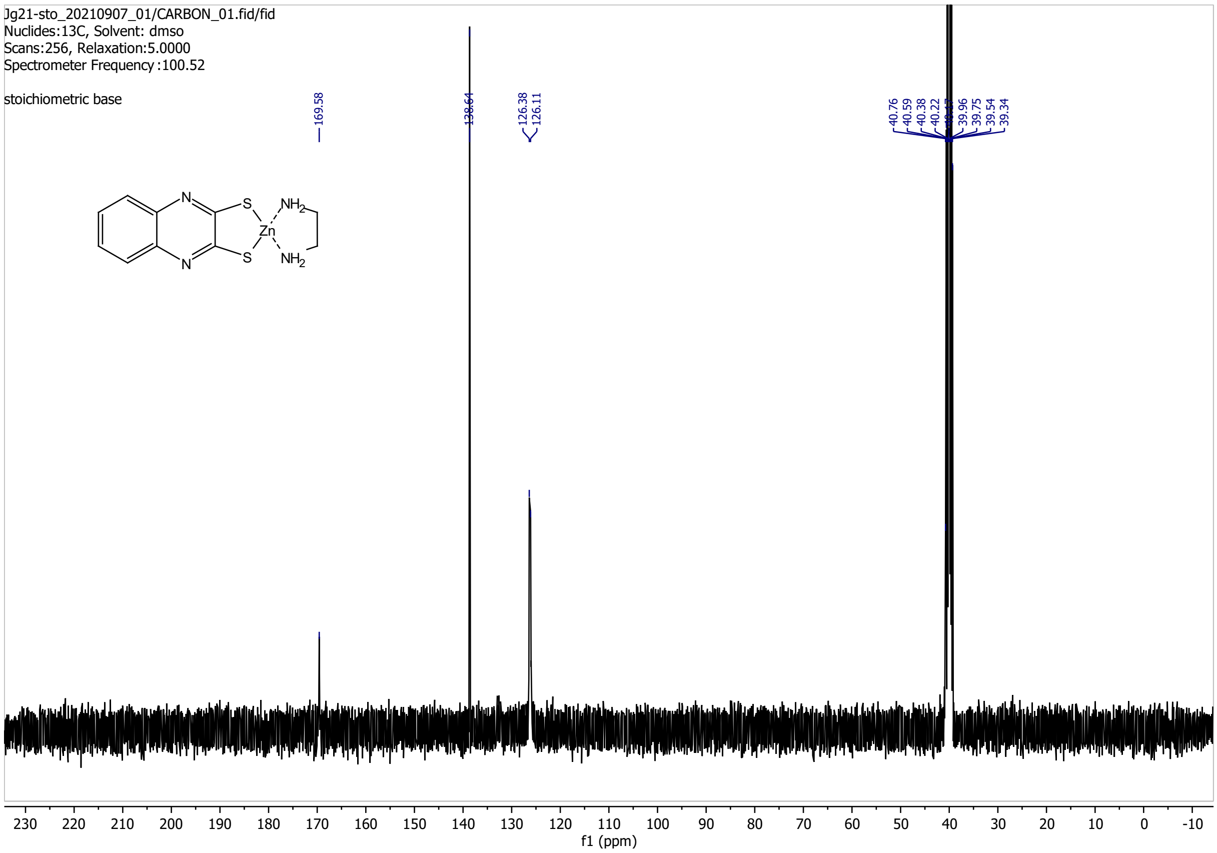

Figure S13. ^{13}C NMR (101 MHz, DMSO-*d6*) of dithioquinoxaline-diamine zinc complex (8)

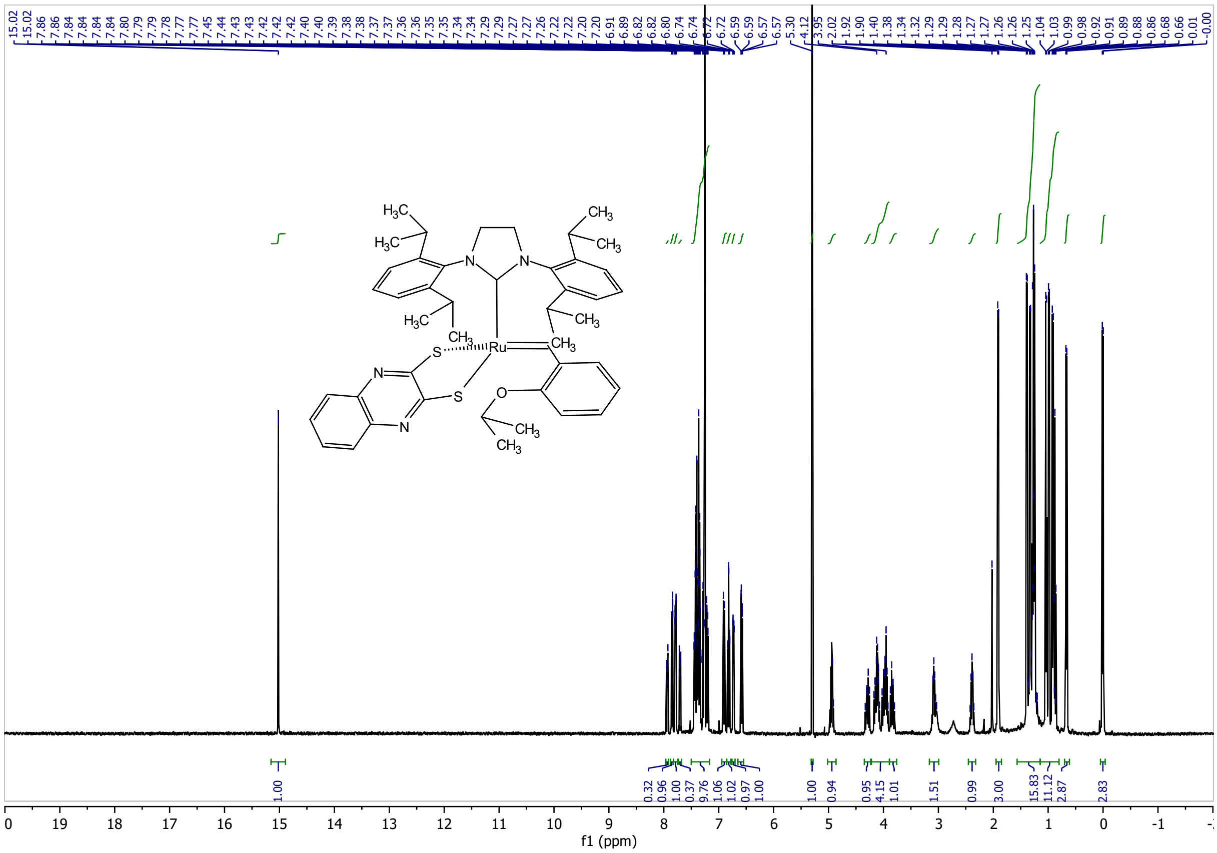

Figure S14. ^{1}H NMR (400 MHz, CDCl3) of Ru3

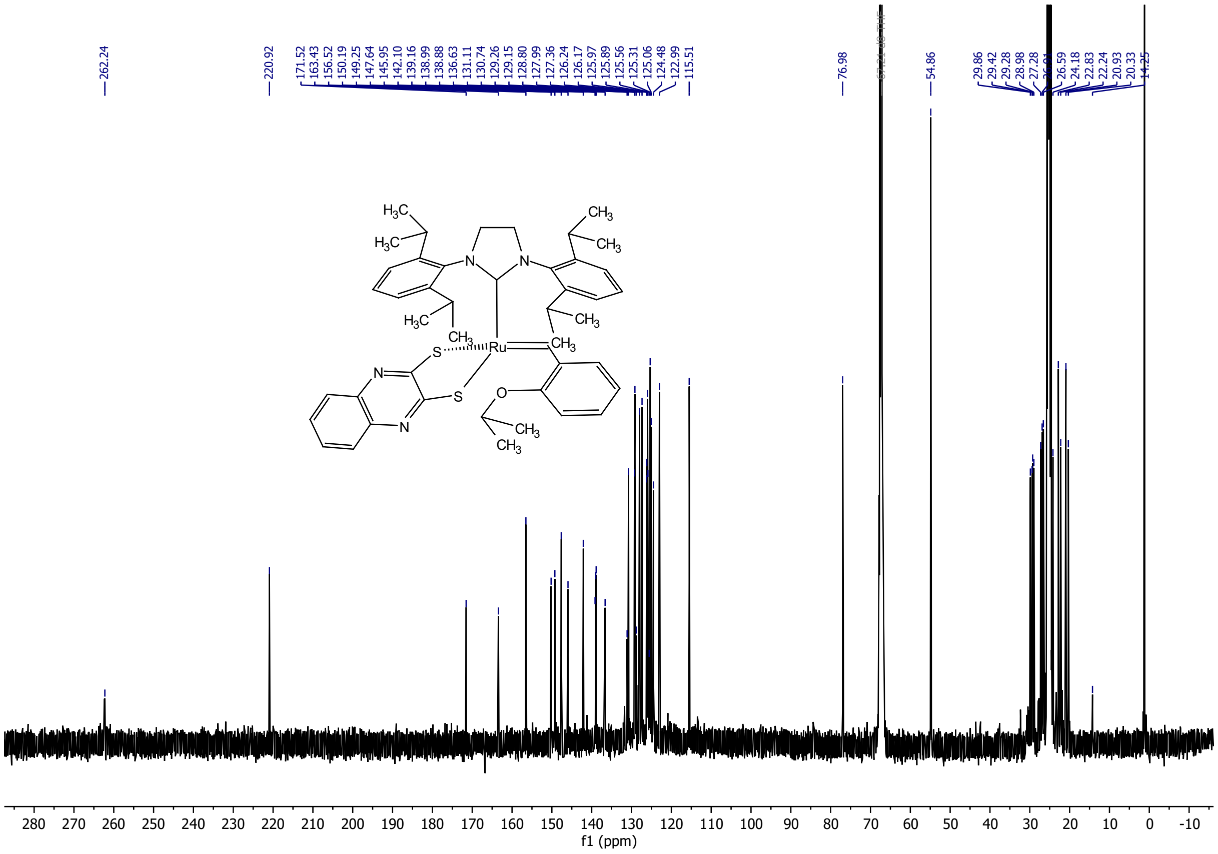

Figure S15. ^{13}C NMR (101 MHz, THF-d_8) of Ru3

2.9.2. Products of metathesis reactions

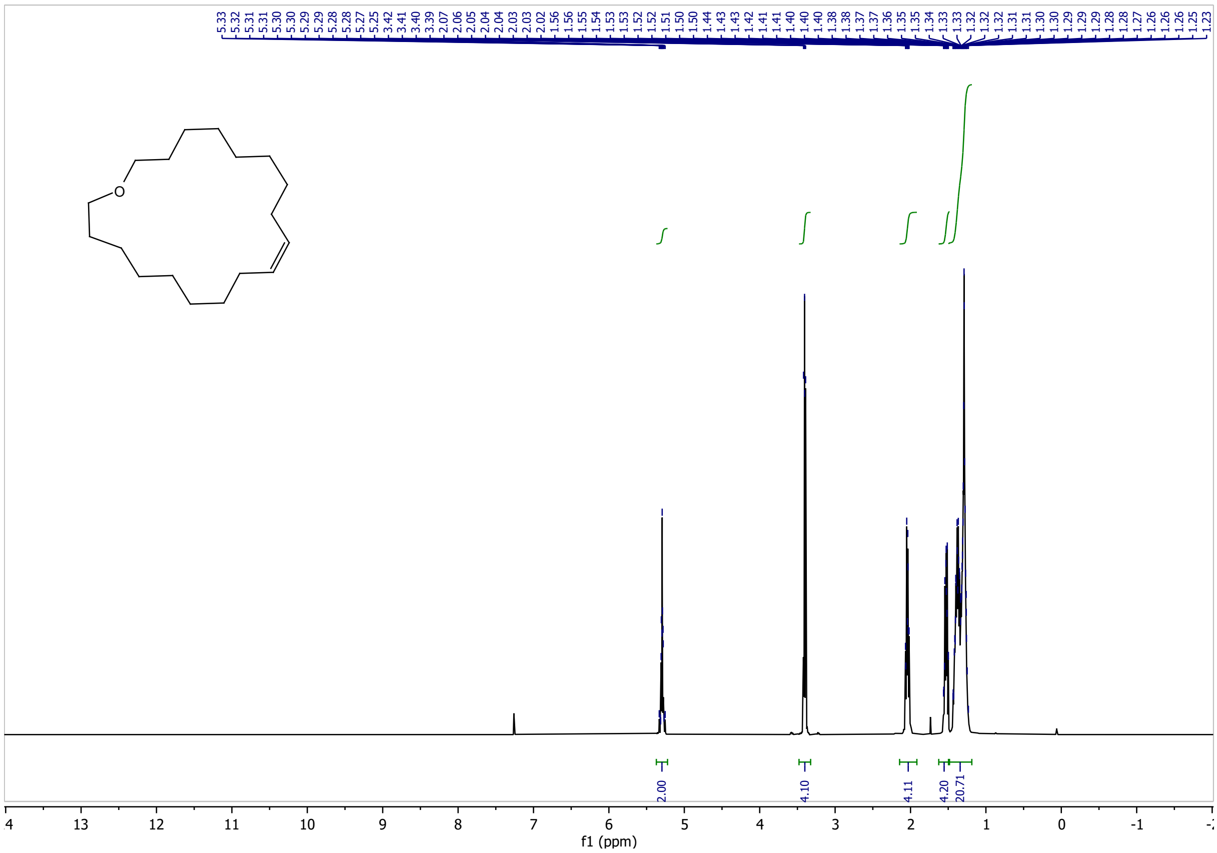

Figure S16. ^{1}H NMR (400 MHz, $CDCl_3$) of (Z)-cycloheptadec-9-en-1-one (13, Civetone)

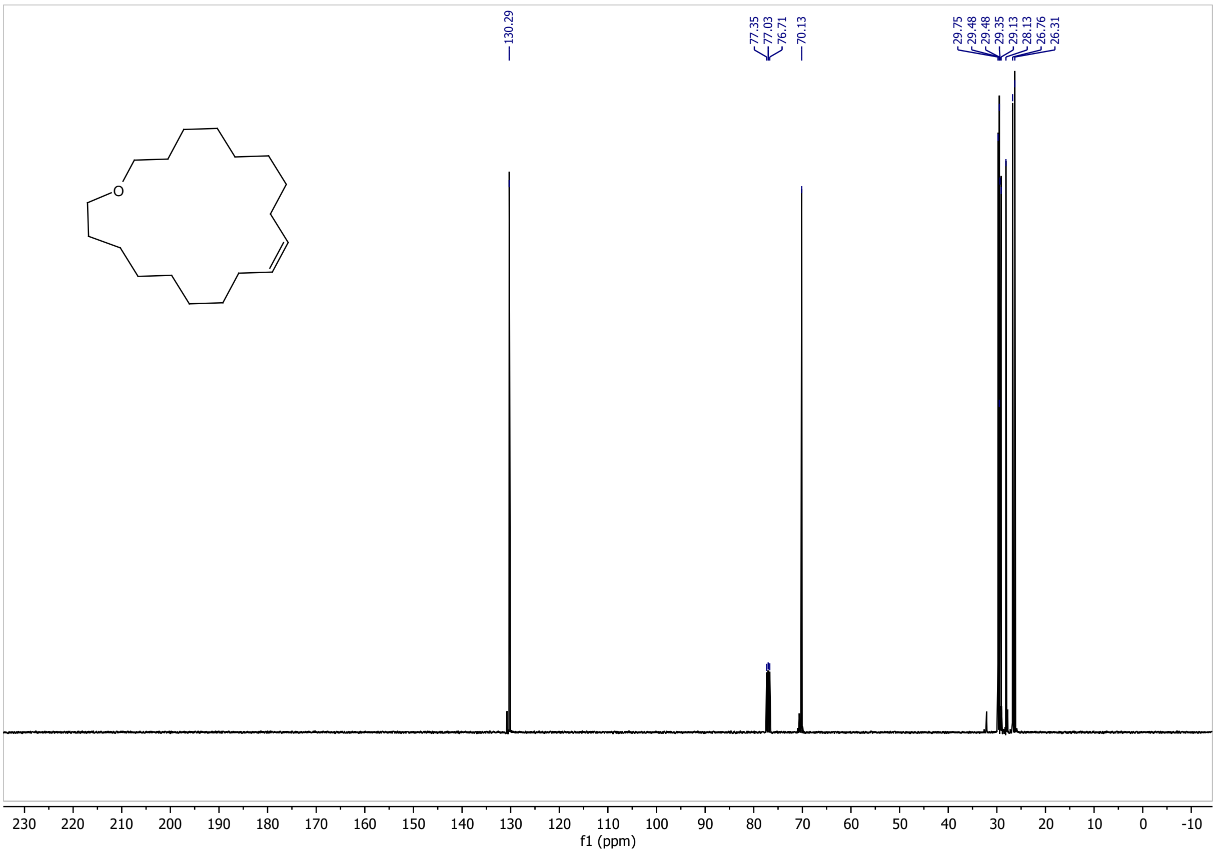

Figure S17. ^{13}C NMR (101 MHz, $CDCl_3$) of (Z)-cycloheptadec-9-en-1-one (13, Civetone)

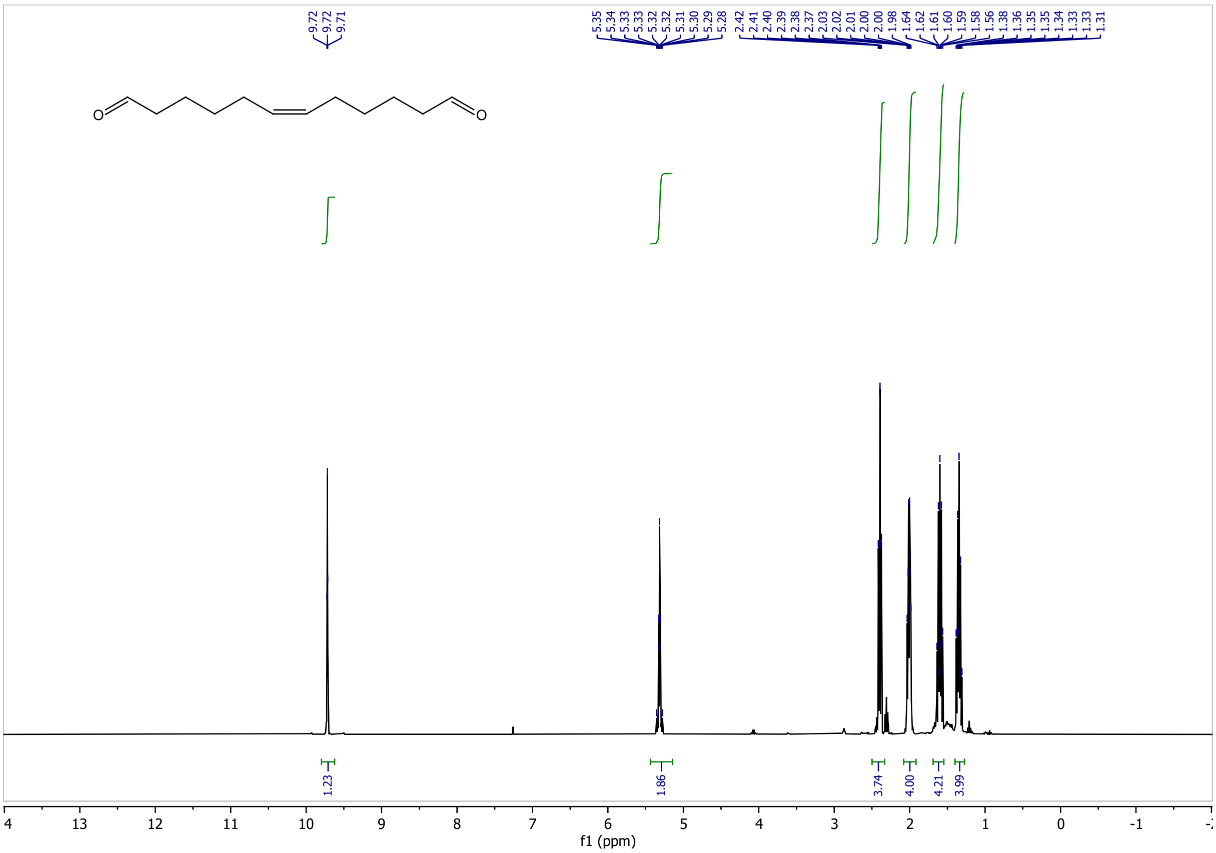

Figure S18. ^{1}H NMR (400 MHz, $CDCl_3$) of (Z)-dodec-6-enedial

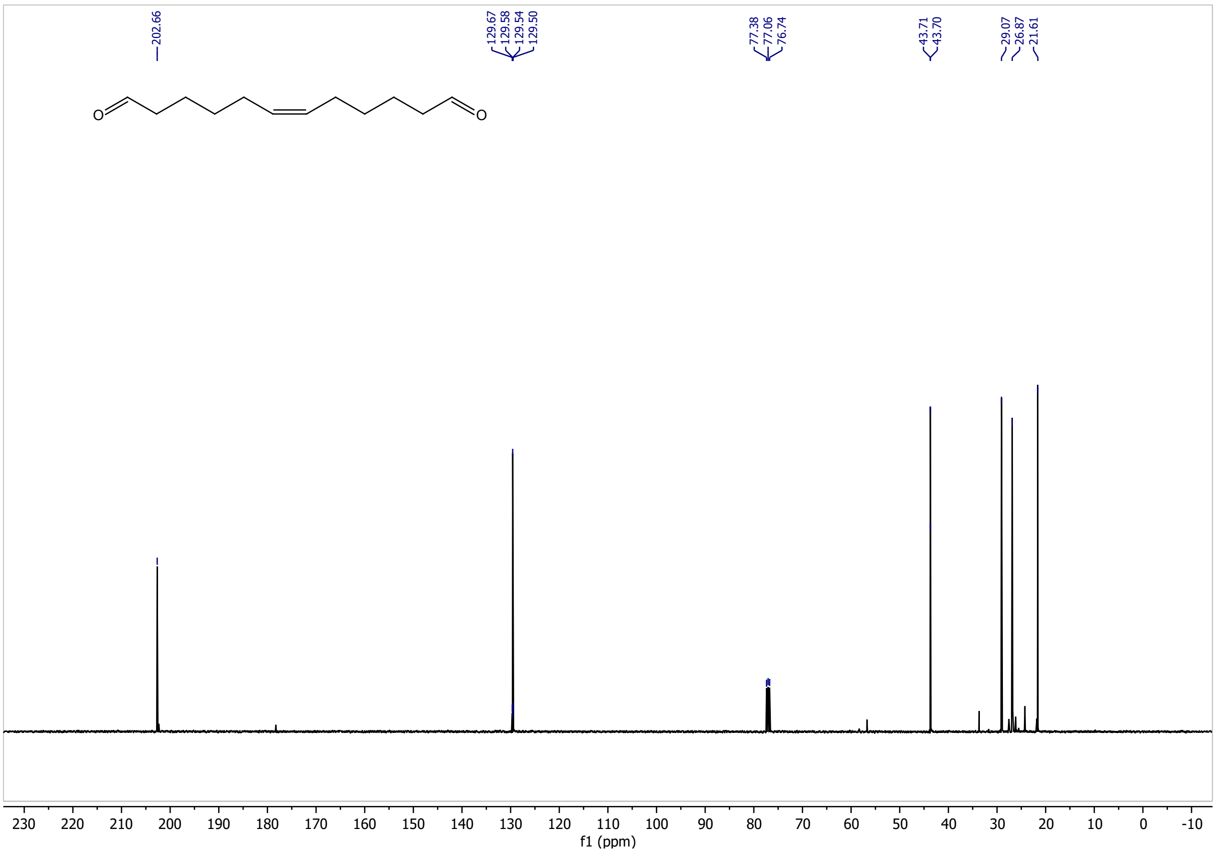

Figure S19. ^{13}C NMR (101 MHz, $CDCl_3$) of (Z)-dodec-6-enedial

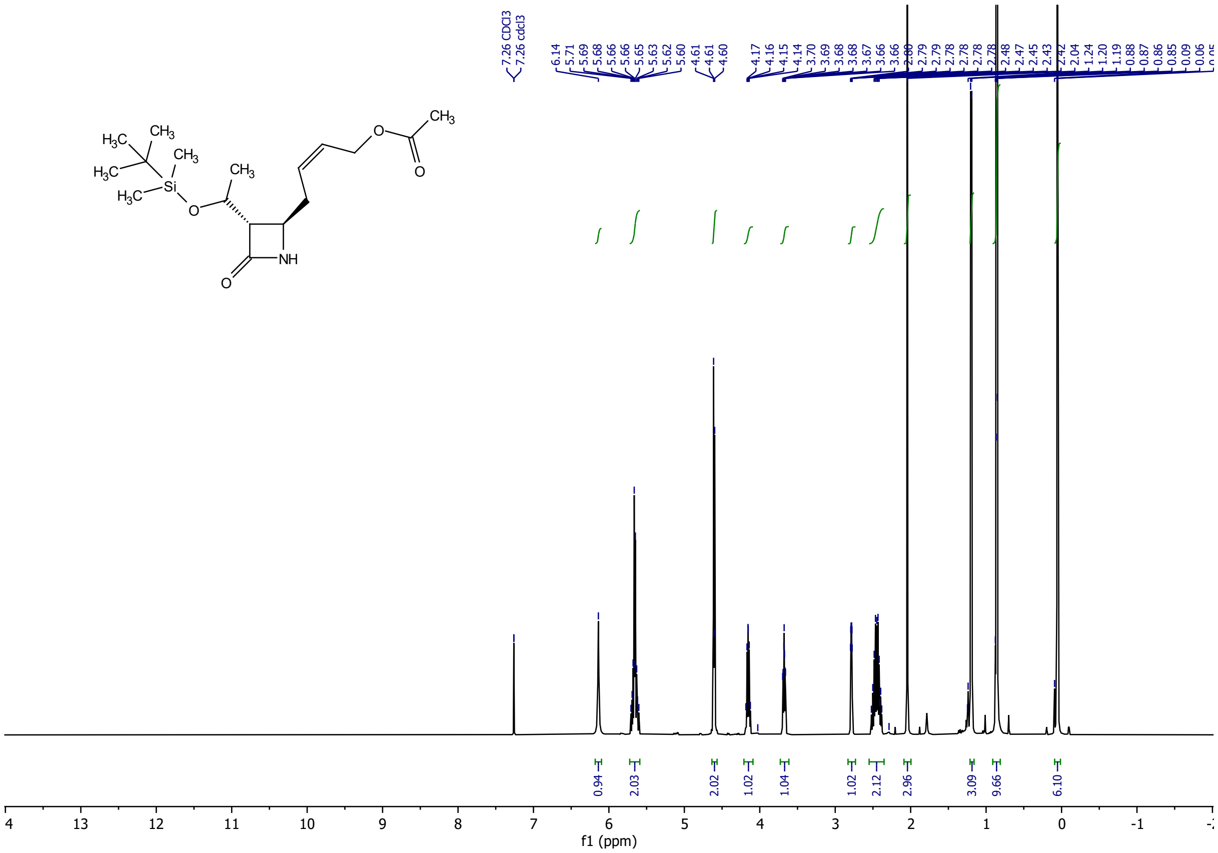

Figure S20. ^{1}H NMR (400 MHz, $CDCl_3$) of (Z)-4-((2R,3S)-3-((S)-1-((tert-butyldimethylsilyl)oxy)ethyl)-4-oxoazetidine-2-yl)but-2-en-1-yl acetate (30)

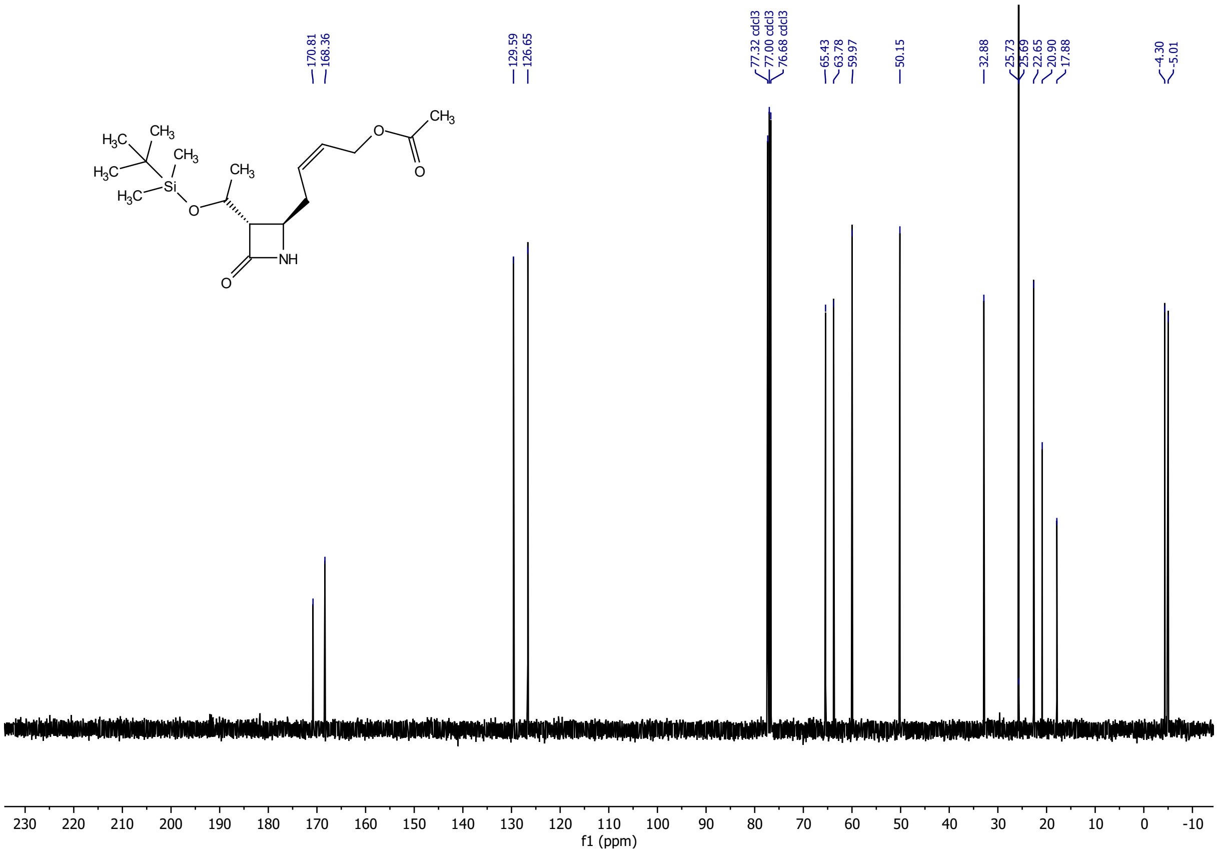

Figure S21. ^{13}C NMR (101 MHz, $CDCl_3$) of (Z)-4-((2R,3S)-3-((S)-1-((tert-butyldimethylsilyl)oxy)ethyl)-4-oxo¬azetidine-2-yl)but-2-en-1-yl acetate (30)

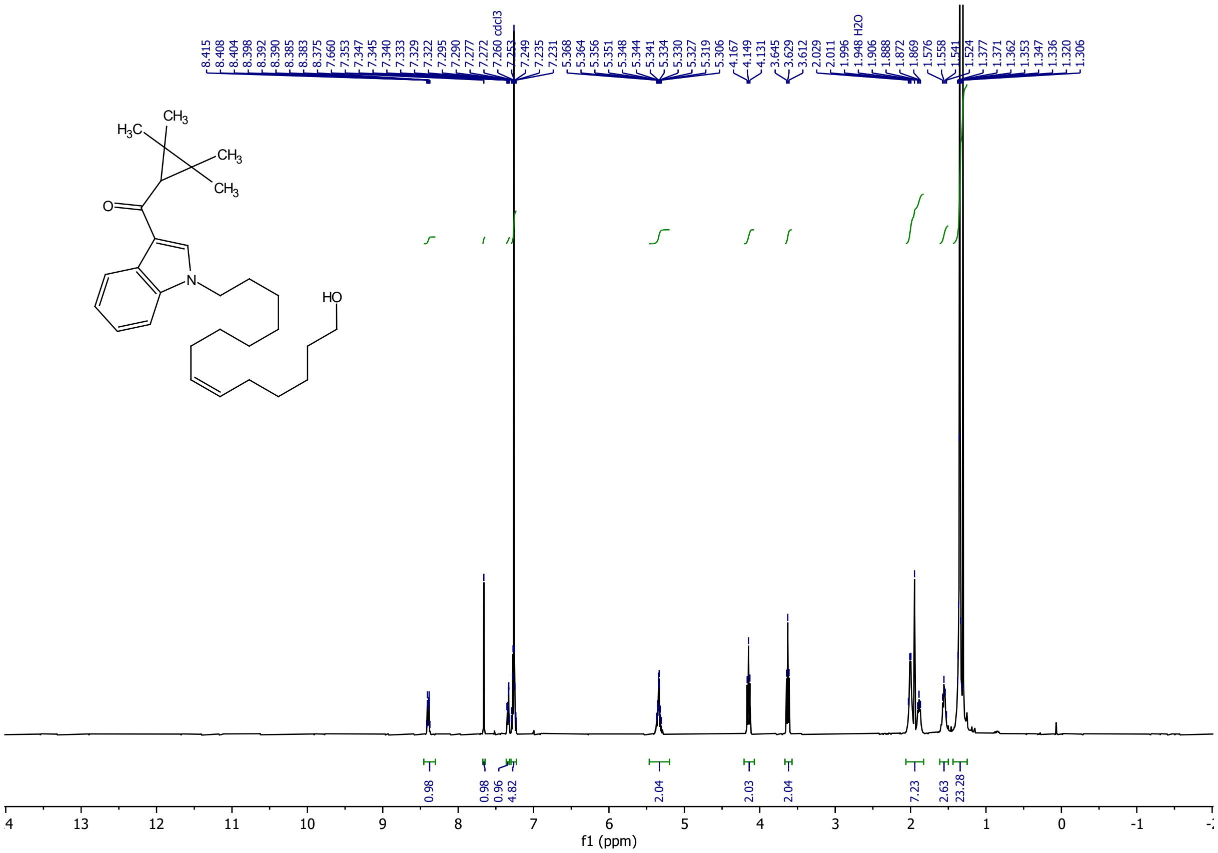

Figure S22. ^{1}H NMR (400 MHz, $CDCl_3$) of (Z)-(1-(14-hydroxytetradec-8-en-1-yl)-1H-indol-3-yl)(2,2,3,3 -tetramethylcyclopropyl)methanone (31)

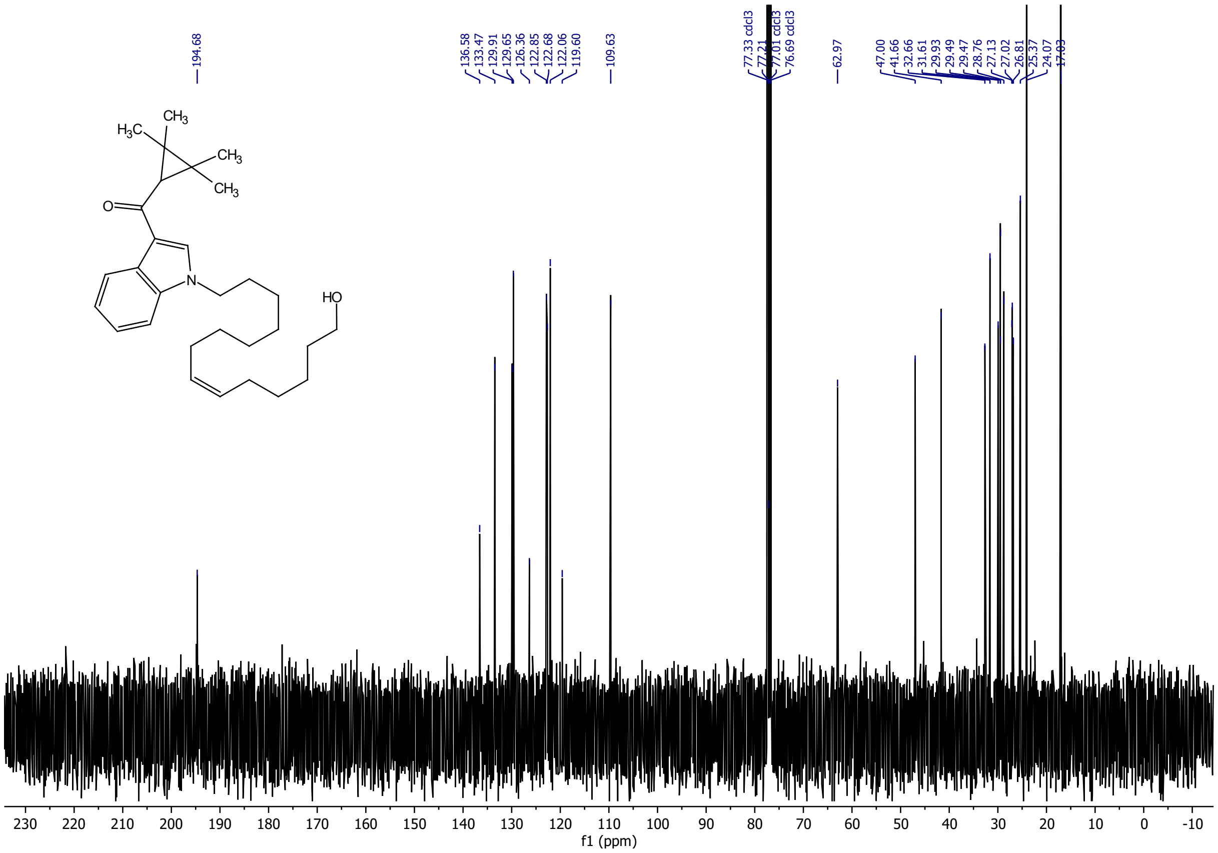

Figure S23. ^{13}C NMR (101 MHz, $CDCl_3$) of (Z)-(1-(14-hydroxytetradec-8-en-1-yl)-1H-indol-3-yl)(2,2,3,3 -tetramethylcyclopropyl)methanone (31)

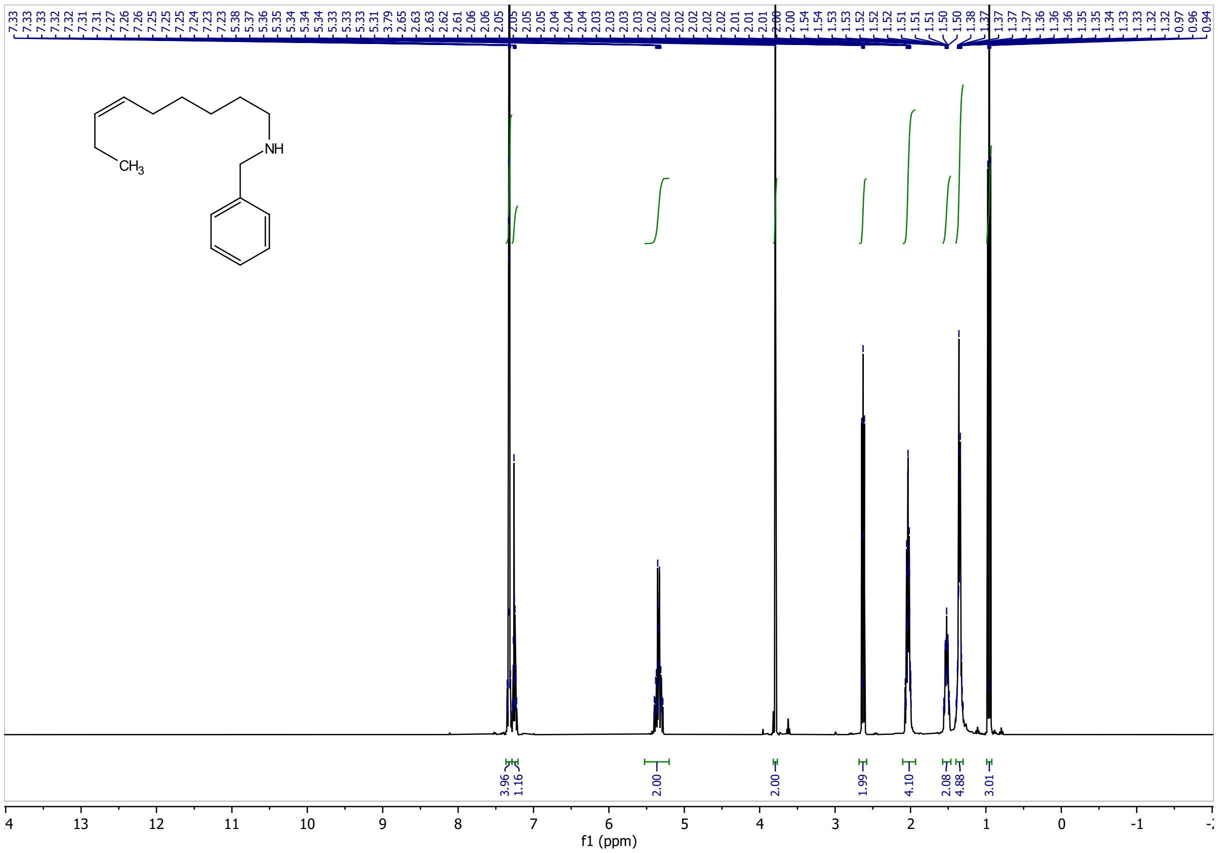

Figure S24. ^{1}H NMR (400 MHz, $CDCl_3$) of of (Z)-N-benzylnon-6-en-1-amine

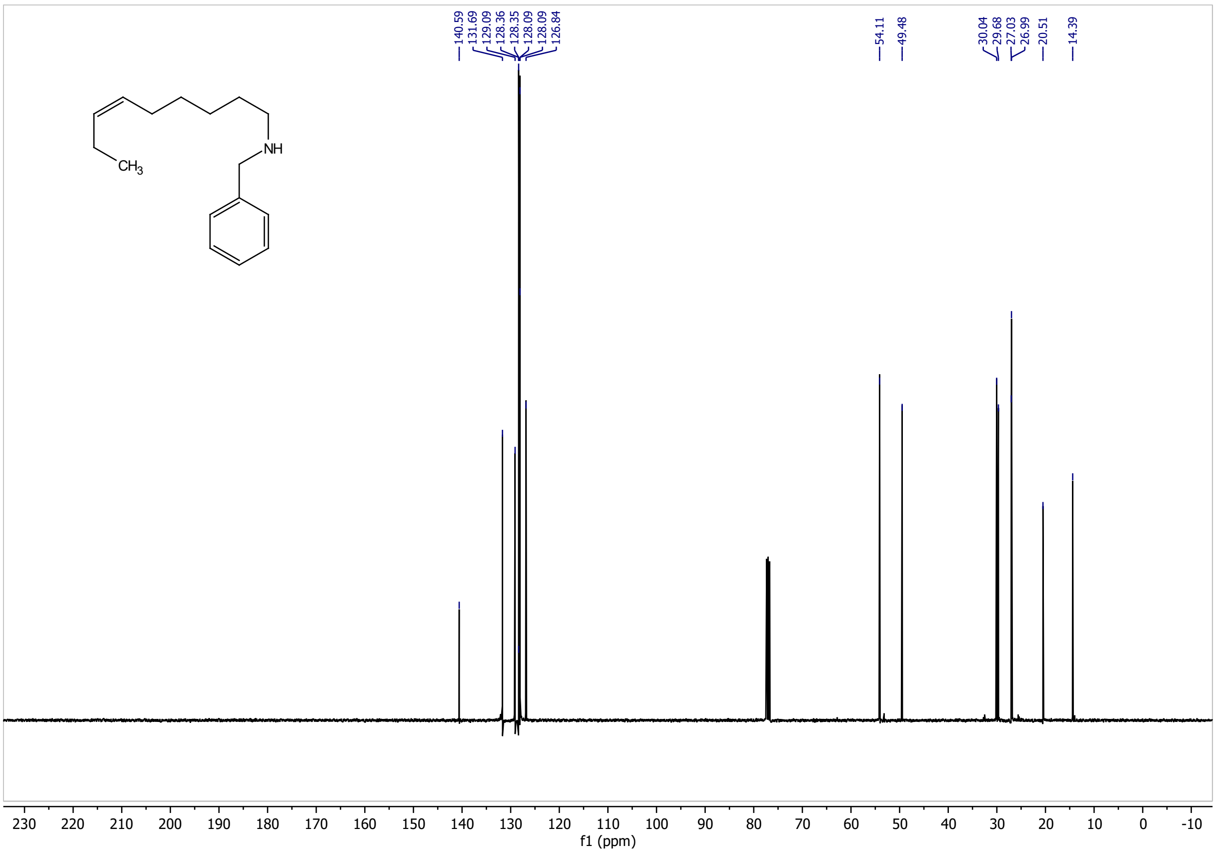

Figure S25. ^{13}C NMR (101 MHz, $CDCl_3$) of (Z)-N-benzylnon-6-en-1-amine

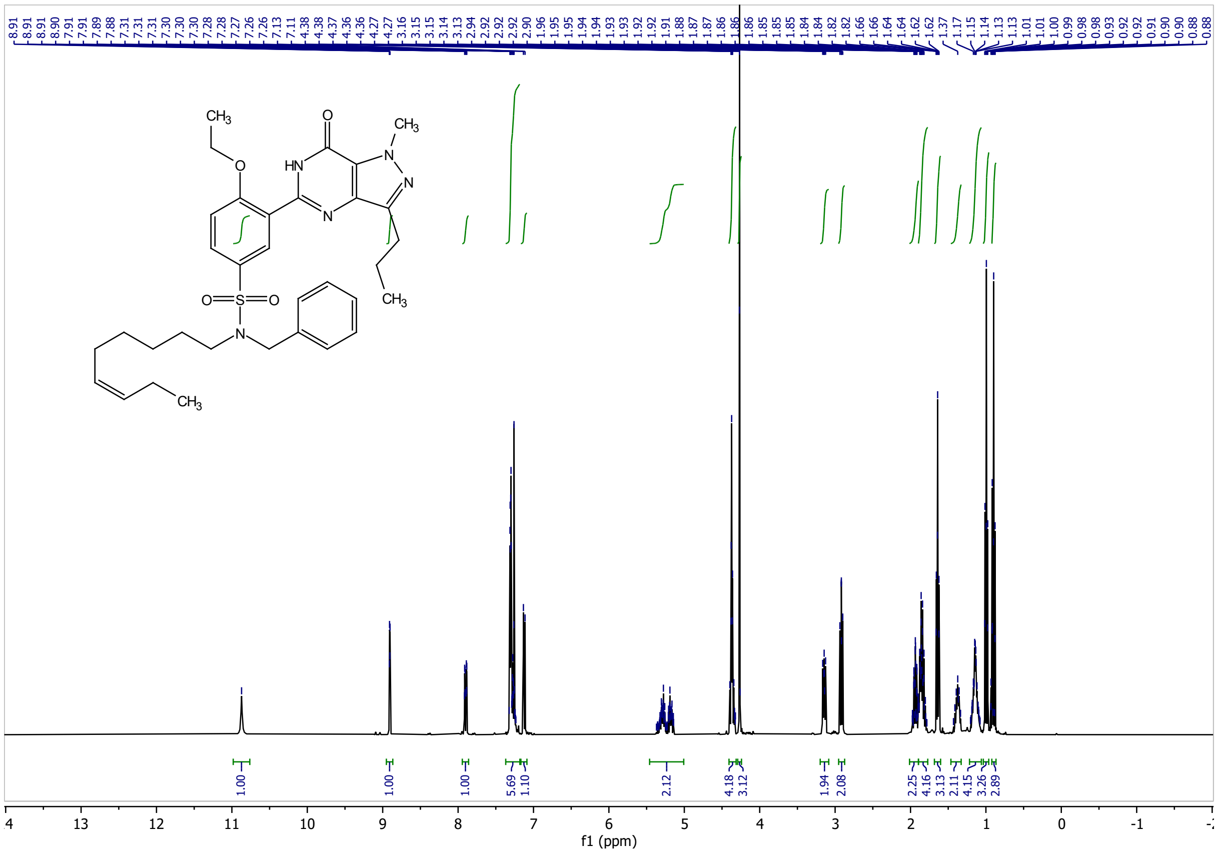

Figure S26. ^{13}C NMR (101 MHz, $CDCl_3$) of (Z)-N-benzyl-4-ethoxy-3-(1-methyl-7-oxo-3-propyl-6,7-dihydro-1H-pyrazolo[4,3-d]pyrimidin-5-yl)-N-(non-6-en-1-yl)benzene-sulfonamide

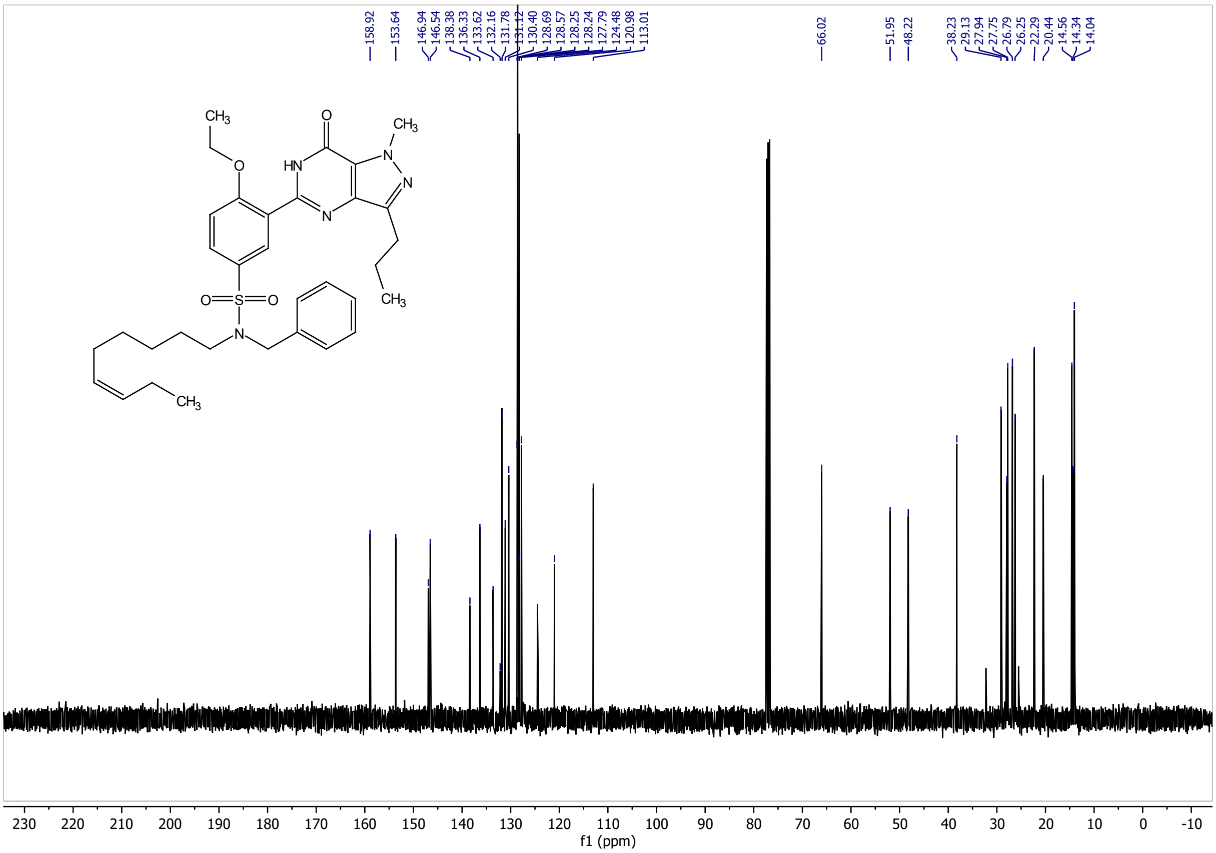

Figure S27. ^{13}C NMR (101 MHz, $CDCl_3$) of (Z)-N-benzyl-4-ethoxy-3-(1-methyl-7-oxo-3-propyl-6,7-dihydro-1H-pyrazolo[4,3-d]pyrimidin-5-yl)-N-(non-6-en-1-yl)benzene-sulfonamide

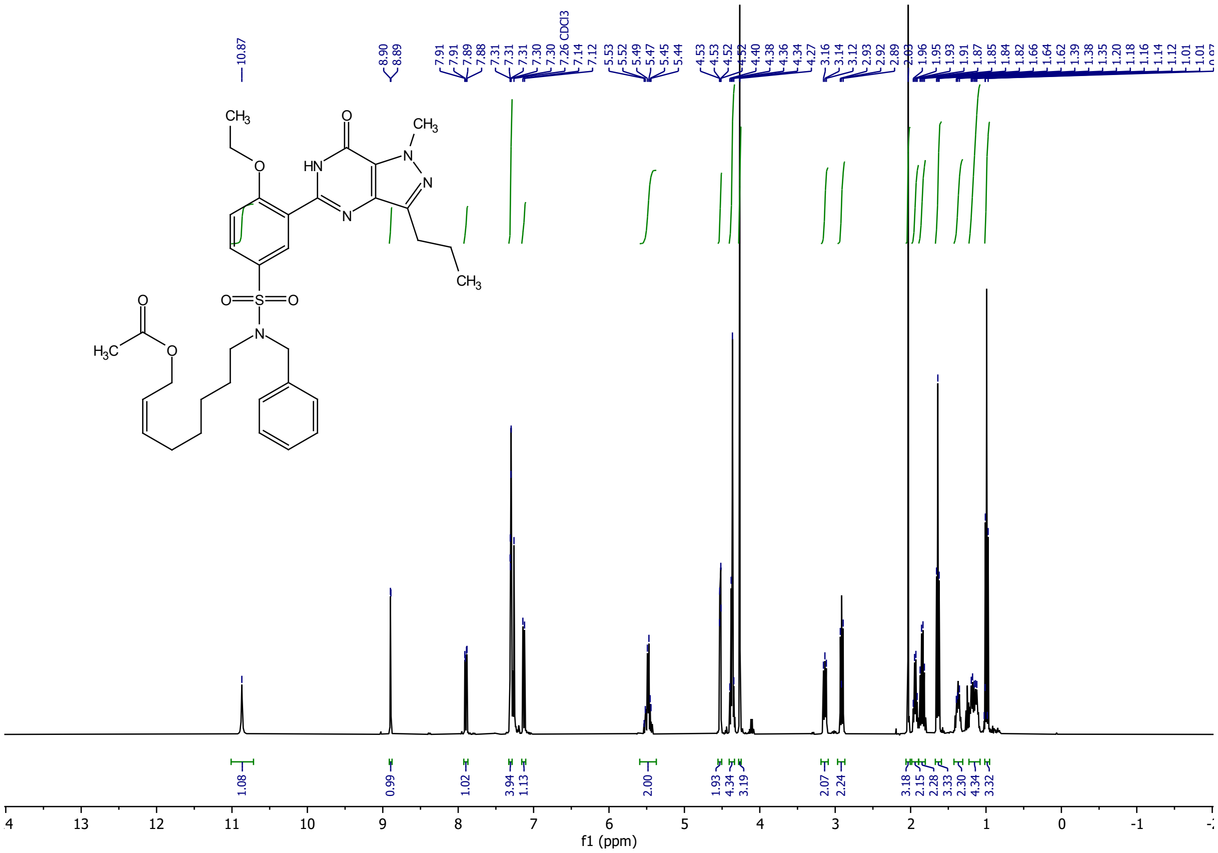

Figure S28. ^{1}H NMR (400 MHz, $CDCl_3$) of (Z)-9-((4-ethoxy-3-(1-methyl-7-oxo-3-propyl-6,7-dihydro-1H-pyrazolo[4,3-d]pyrimidin-5-yl)phenyl)sulfonyl)-10-phenyldec-2-en-1-yl acetate

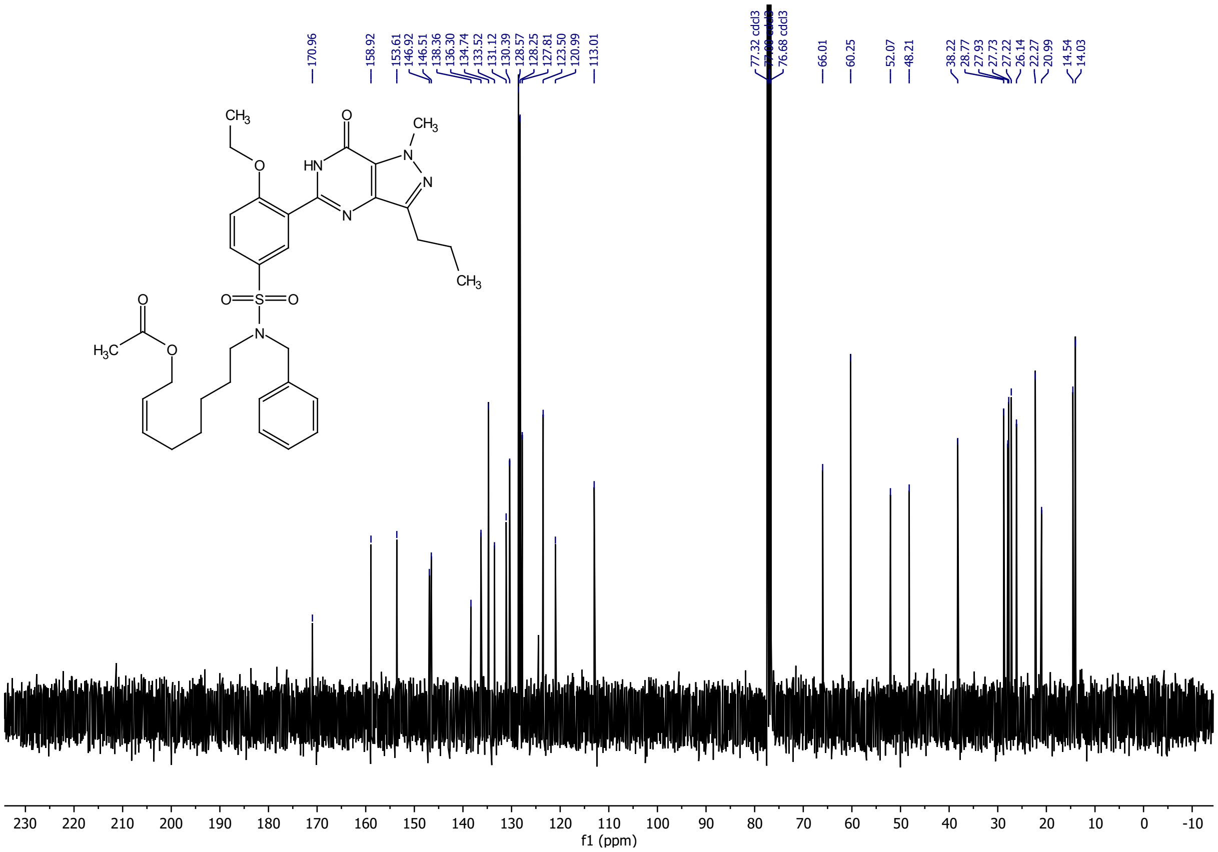

Figure S29. ^{13}C NMR (101 MHz, $CDCl_3$) of (Z)-9-((4-ethoxy-3-(1-methyl-7-oxo-3-propyl-6,7-dihydro-1H-pyrazolo[4,3-d]pyrimidin-5-yl)phenyl)sulfonyl)-10-phenyldec-2-en-1-yl acetate

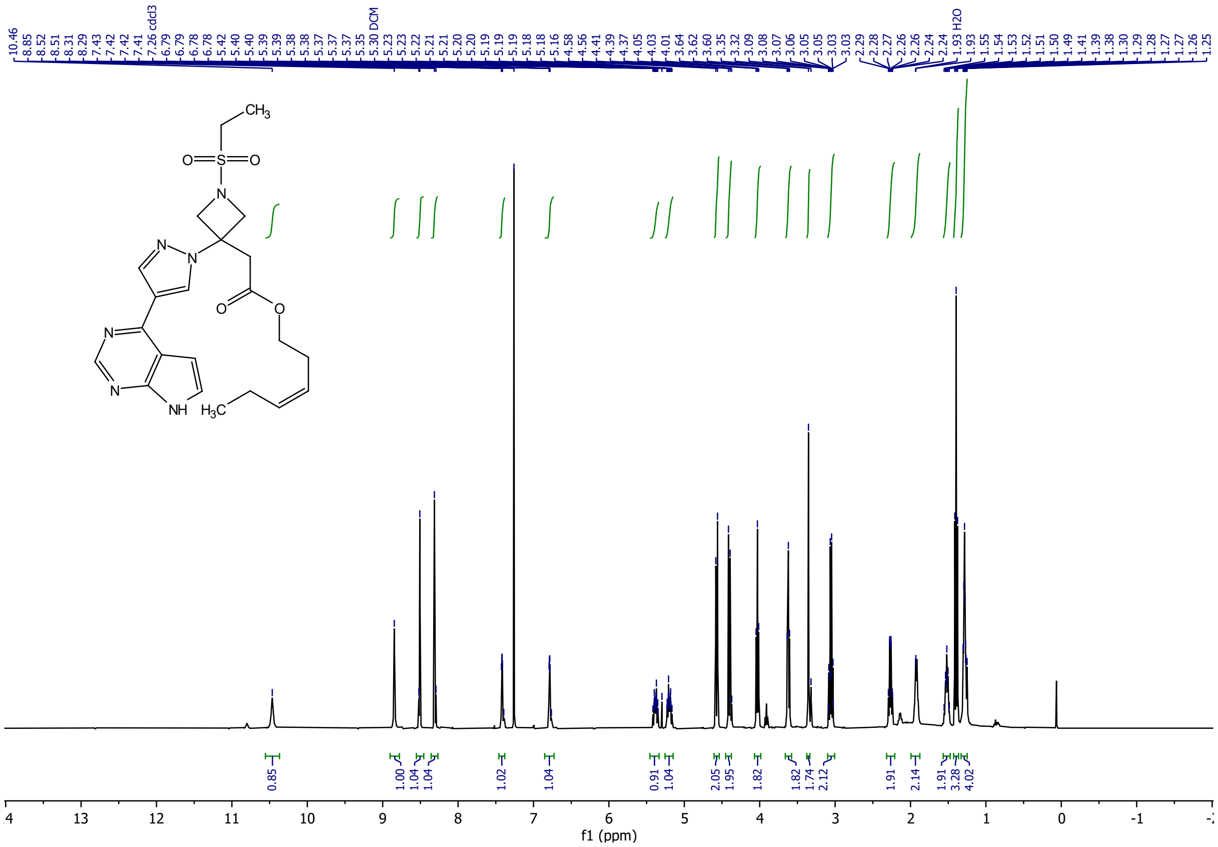

Figure S30. ^{1}H NMR (400 MHz, $CDCl_3$) of (*Z*)-hex-3-en-1-yl-2-(3-(4-(7H-pyrrolo[2,3-d]pyrim¬idin-4-yl)-1H-pyrazol-1-yl)-1-(ethylsulfonyl)azetidine-3-yl)acetate

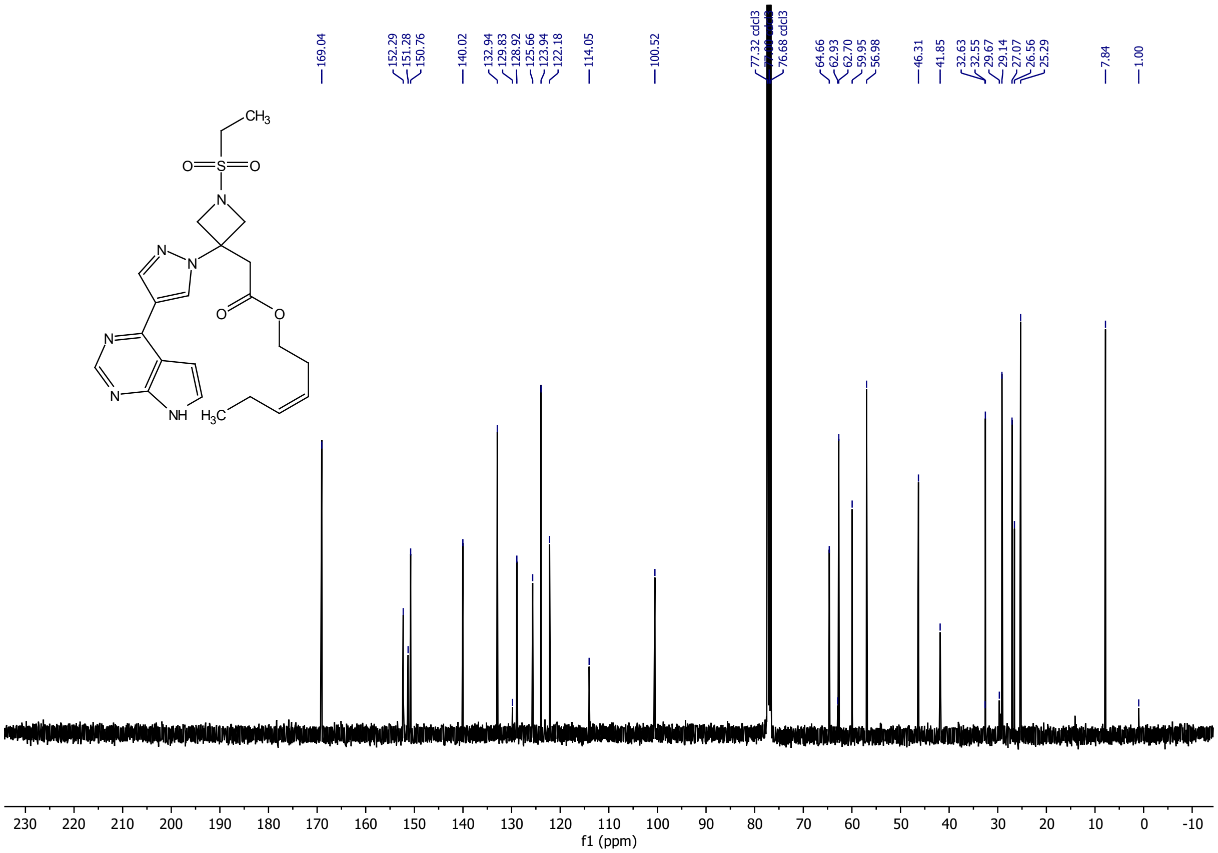

Figure S31. ^{13}C NMR (101 MHz, $CDCl_3$) of (*Z*)-hex-3-en-1-yl-2-(3-(4-(7H-pyrrolo[2,3-d]pyrimidin-4-yl)-1H-pyrazol-1-yl)-1-(ethylsulfonyl)azetidine-3-yl)acetate

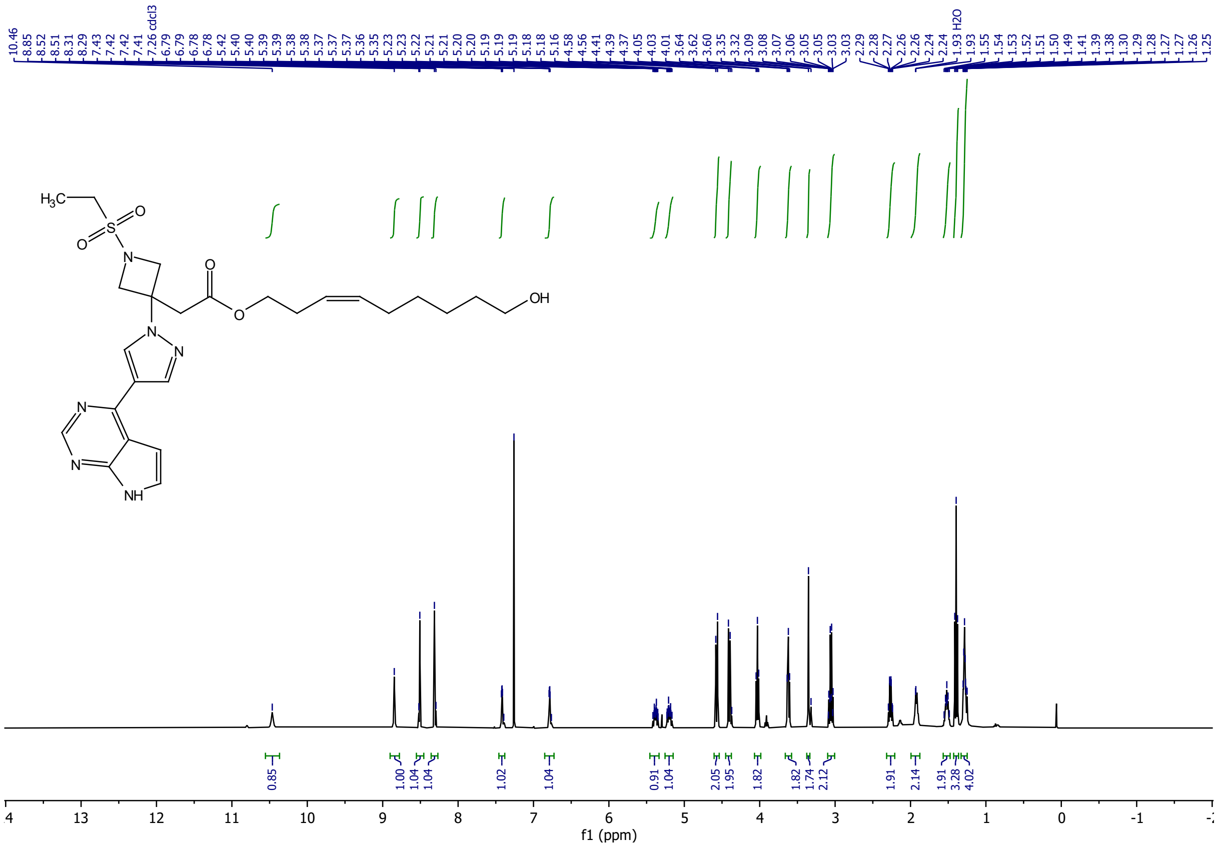

Figure S32. ^{1}H NMR (400 MHz, $CDCl_3$) of (Z)-9-hydroxynon-3-en-1-yl 2-(3-(4-(7H-pyrrolo[2,3-d]pyrimidin-4-yl)-1H-pyrazol-1-yl)-1-(ethylsulfonyl)azetidin-3-yl)acetate

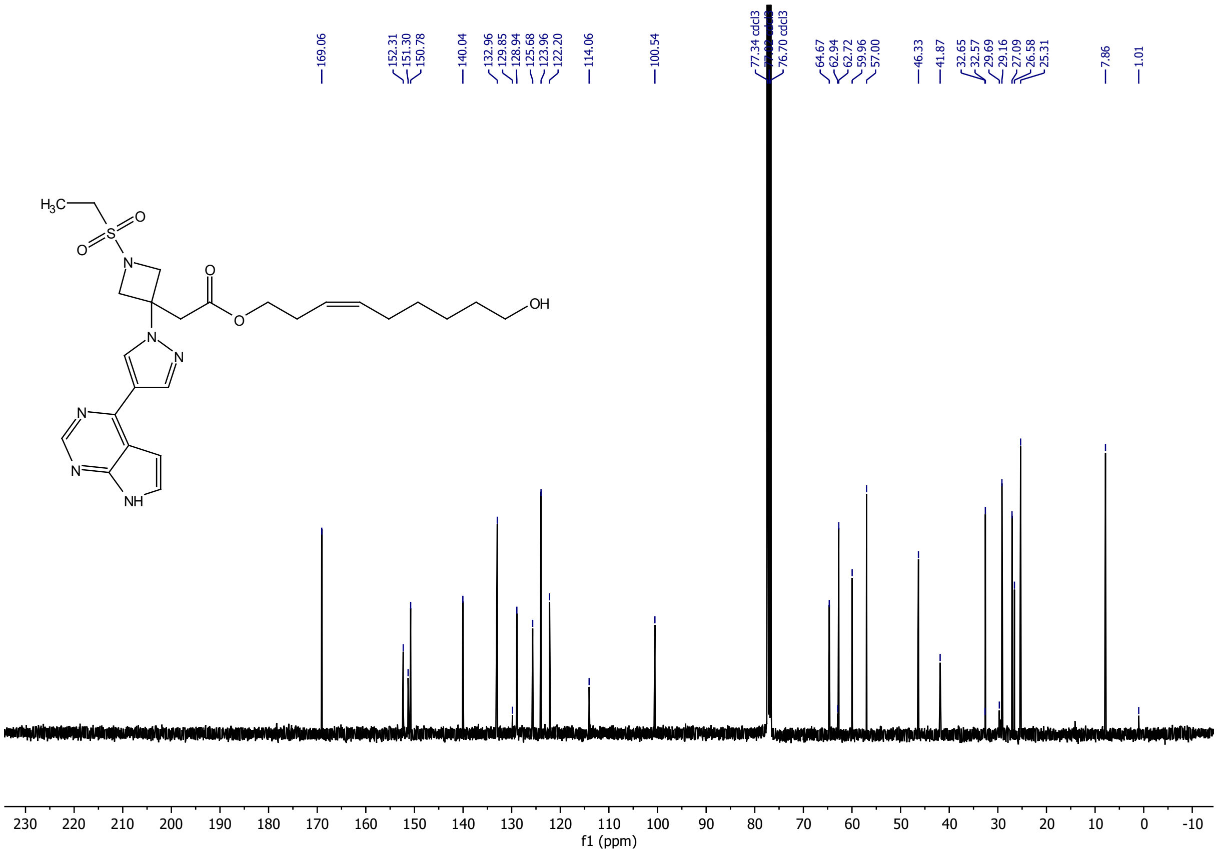

Figure S33. ^{13}C NMR (101 MHz, $CDCl_3$) of (Z)-9-hydroxynon-3-en-1-yl 2-(3-(4-(7H-pyrrolo[2,3-d]pyrimidin-4-yl)-1H-pyrazol-1-yl)-1-(ethylsulfonyl)azetidin-3-yl)acetate

2.10. X-Ray

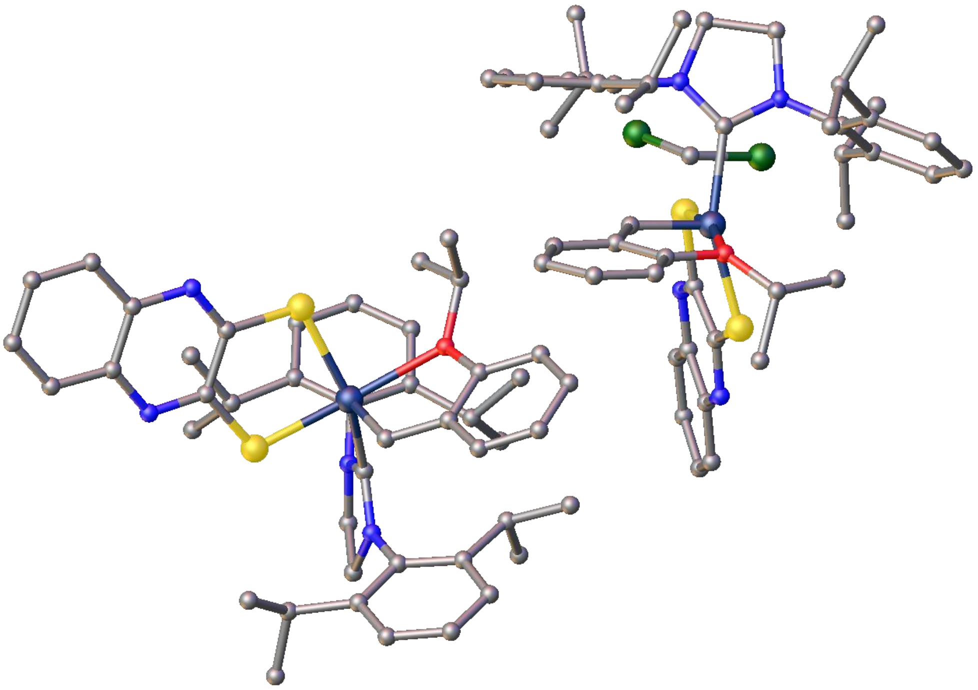

Table S6. Crystal data and structure refinement for 2021_05_27_DT_Ru3_C.

CCDC Deposition Number	2350437
Identification code	2021_05_27_DT_Ru3_C
Empirical formula	$C_{181}H_{212}Cl_2N_{16}O_4Ru_4S_8$
Formula weight	3407.31
Temperature/K	100(2)
Crystal system	orthorhombic
Space group	Pccn
a/Å	32.3333(7)
b/Å	32.1832(7)
c/Å	18.2677(5)
α/°	90
β/°	90
γ/°	90
Volume/Å^3	19009.2(8)
Z	4

ρ_{calc}g/cm^3	1.191
μ/mm^{-1}	4.011
F(000)	7120.0
Crystal size/mm^3	0.341 × 0.302 × 0.25
Radiation	CuKα (λ = 1.54184)
2Θ range for data collection/°	5.466 to 134.16
Index ranges	−37 ≤ h ≤ 38, −26 ≤ k ≤ 38, −21 ≤ l ≤ 19
Reflections collected	48212
Independent reflections	16953 [R_{int} = 0.0461, R_{sigma} = 0.0457]
Data/restraints/parameters	16953/0/998
Goodness-of-fit on F^2	1.028
Final R indexes [I>=2σ (I)]	R_1 = 0.0682, wR_2 = 0.1729
Final R indexes [all data]	R_1 = 0.0855, wR_2 = 0.1902
Largest diff. peak/hole / e Å^{-3}	2.08/ −0.93

2.11. Computational Study

2.11.1. Computational Details

DFT computations were conducted using the Gaussian16 suite of programs.[27] Geometry optimisations and frequency calculations utilised the BP86 functional,[28,29] augmented with the Grimme dispersion correction GD3.[30,31] Molecular electronic configurations were determined using a split-valence basis set incorporating polarisation functions developed by Ahlrichs and colleagues for H, C, N, and Cl (specified by the Def2SVP keyword in Gaussian16).[32] Specifically, for Ru, a small-core, quasi-relativistic Stuttgart/Dresden effective core potential was applied, accompanied by a contracted valence basis set (indicated by standard SDD keywords in Gaussian16).[33–35] Geometry optimisations were performed without symmetry constraints, and the identified stationary points were characterised using analytical frequency calculations. Gibbs energies (ΔG) were computed incorporating zero-point energy, thermal, and entropic corrections obtained from gas-phase frequency calculations at the BP86/Def2SVP level of theory. Single-point energy calculations were carried out on BP86/Def2SVP geometries using the M06L functional,[36] and a triple-ζ valence plus polarisation basis set for main group atoms (def2TZVP keyword in Gaussian),[37] with solvent effects accounted for using the PCM model and THF as the solvent.[38,39]

2.11.2. Computational Results

		Lewis Structure			
Lewis	non-Lewis	CR	BD	nC	LP
104.01326	1.98674	26	15	0	12
(98.126% of 106)	(1.874% of 106)				

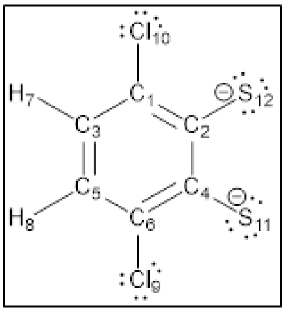

Preliminary tautomerisation study of the protonated forms of the ligands *via* DFT (energies in kcal/mol corresponding to the gas Gibbs energies).

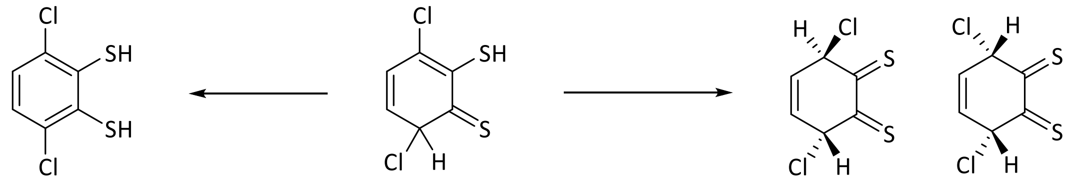

isomer1 0.0 | isomer2 25.7 | isomer3_trans 40.8 | isomer3_cis 38.0

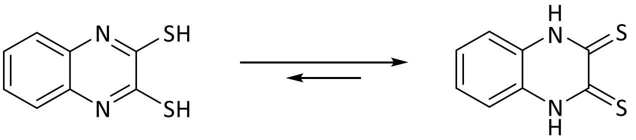

stquin1 6.6 | stquin2 0.0

Particularly, the NBO analysis of the optimised thiocatechol in Table S7 gives 12 lone-pairs, distributed as the expected Lewis structure one might expect: 3 lone-pairs to each chlorine atom and 3 more to each sulphide atom. Nevertheless, the analysis also shows a considerable high non-Lewis index (1.874%) meaning some discordance with the pure Lewis scheme.

MATRIX

LONE 9 3 10 3 11 3 12 3 END

BOND D 1 2 S 1 3 S 1 10 S 2 4 S 2 12 D 3 5 S 3 7 D 4 6 S 4 11 S 5 6 S 5 8 S 6 9 END

This notation is from the Natural Bond Orbitals program to make reference to an specific hybrid of resonance within a molecule. Particularly, it can be obtained from the program or introduced in the input to force an specific rearrangement. Often, like in our case, it can be used to ease the analysis and comparison when dealing with aromatic or multiple lone-pairs. Using this strategy it is possible to avoid the algorithm to check a huge amount of combinations that would not have chemical meaning and thus, they should also be eliminated anyway. In particular, the sequence starting with LONE has the following meaning: LONE = lone-pairs location; 1 (atom number, in

our case a sulphide) 3 (number of lone-pairs) 4 (next atom number, also sulphide) 3 (again three lone-pairs) 5 (next atom, a nitrogen) 1 (only one lone pair) ...; BOND= type of bonding; 1 2 S (atoms 1 and 2 with a single bond) 2 3 D (atoms 2 and 3 with a double bond) ....

Table S7. Orbital analysis by NBO on the occupancy.

**Lewis**		**(Occupancy)**
Cl9	LP	1.99210
	LP	1.97511
	LP	1.95865
Cl10	LP	1.99210
	LP	1.97511
	LP	1.95865
S11	LP	1.95865
	LP	1.95865
	LP	1.70344
S12	LP	1.95865
	LP	1.95865
	LP	1.70344
double C-C bonds		
(1) C1-C2	BD	1.98335
(2) C1-C2	BD	1.71411
(1) C3-C5	BD	1.96741
(2) C3-C5	BD	1.78750
(1) C4-C6	BD	1.98335
(2) C4-C6	BD	1.71410
**Non-Lewis**		
(2) C1-C2	BD*	−0.47358
(2) C3-C5	BD*	−0.45837
(2) C4-C6	BD*	−0.47358

Further orbital analysis in Table S7 reveals a decrease in occupancy of the third lone-pairs (1.95865 vs 1.70344). This indicates a partial donation of density from the sulphide lone-pair, offering a potential explanation for the previously mentioned non-Lewis index. No other significant changes in occupancies are observed in lone-pairs or bonding orbitals.

On the antibonding side, higher occupancies are detected in molecular orbitals arising from the C1-C2 bond, C4-C6 bond (as expected based on symmetry), and C3-C5 bond. Visualisation of these orbitals illustrates the overlap between the BD* C1-C2 and the S12 LP (below).

Second Order Perturbation Analysis in Table S8 confirms the significance of the donation from the third LP of the S11 to the antibonding C4-C6, with an energy of 44.9 kcal/mol.

Table S8. Report of the NBO's analysis (2nd order perturbation) and visualisation of discussed orbitals.

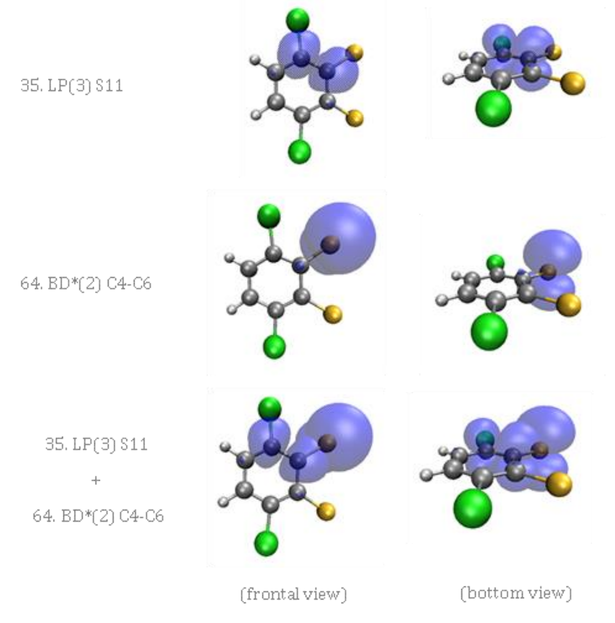

Donor (L)	Acceptor (NL)	E(2)
29. LP(3) Cl9	64. BD*(2) C4-C6	10.28
32. LP(3) Cl10	55. BD*(2) C1-C2	10.28
35. LP(3) S11	64. BD*(2) C4-C6	44.93
38. LP(3) S12	55. BD*(2) C1-C2	44.92

In Figure S34, on the left (depicted in blue) there are the Wiberg bond indexes (WBI), while on the right (shown in purple) there are the bond distances of the optimised structure. The WBI serves as an indicator of electron sharing; the closer it is to 1, the higher the probability of finding two electrons between the respective nuclei. In a benzene ring, WBI values are expected to be greater than 1 for all carbon atoms but lower than 2 due to delocalisation.

In the case of thiocatechol, the C-Cl bonds exhibit a WBI of 0.969, indicative of a single bond. Conversely, the C-S bonds have a WBI of 1.307, closer to the C-C bonds of the aromatic ring. Notably, one C-C bond in the ring, specifically the C2-C4 bond, demonstrates a lower-than-expected WBI, accompanied by a distance of 1.513 Å, resembling a single bond. This suggests that the lone-pairs of the sulphide promote a charge delocalisation pattern that disrupts the typical ring-shaped delocalisation of pi orbitals in the benzene ring. The more accurate representation is depicted in the lower right-margin, below:

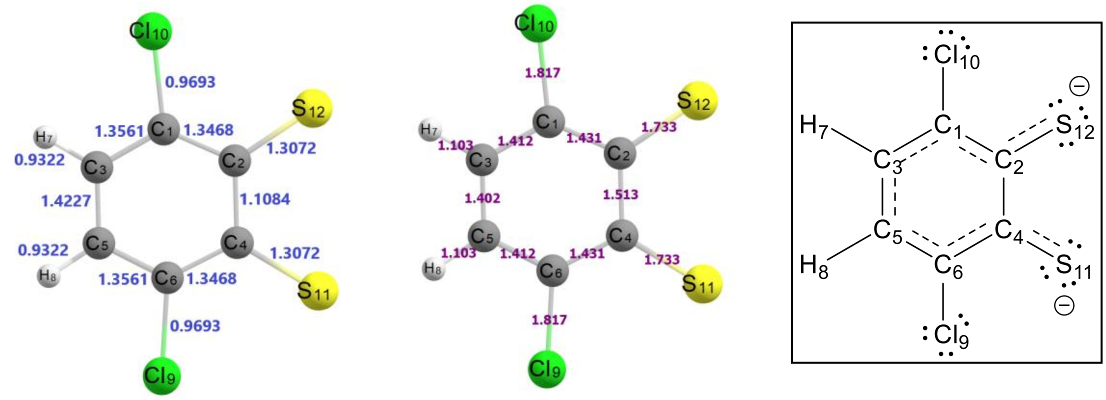

Figure S34. Wiberg bond indexes (left) and distances in Å (right) for thiocatechol.

Other interesting points on the results above are:

-The slightly greater distance observed in the C1-C2 (and C4-C6) bonds is indeed associated with the donation from the S lone-pair to the antibonding orbital illustrated above.

-The higher Wiberg bond index (WBI) observed in the C3-C5 bond, coupled with its shorter distance, designates it as the strongest C-C bond within the ring.

-One would anticipate a lower WBI index in the C-Cl bonds, attributed to the electronegativity of the halide.

Ultimately, the other suggested resonance structures (**B** and **C**) are also scrutinised (see Table S9. The findings reveal that despite a discernible delocalisation in the lone pairs of sulphur to the neighbouring C-C bond, structure **A** emerges as the superior representation of the ligand, boasting the lowest non-Lewis index among the hybrids.

Table S9. Summary of the NBO Analysis for the three proposed resonance structures (and their schematic representation) for thiocatechol A, B, and C (supra) and for the only thioquinoxaline structure evaluated (infra).

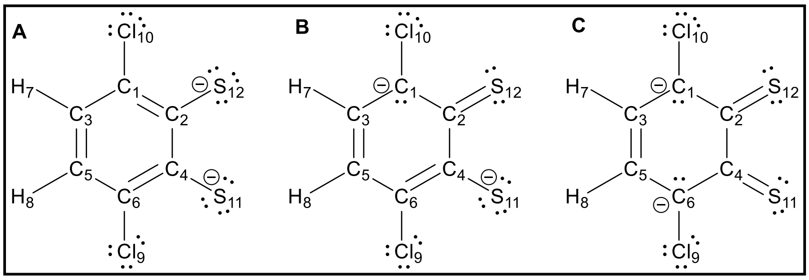

			Lewis Structure			
	Lewis	non-Lewis	CR	BD	nC	LP
A	104.01326 (98.126%)	1.98674 (1.874%)	26	15	0	12
B	103.74767 (97.875%)	2.25233 (2.125%)	26	15	0	12
C	103.48209 (97.625%)	2.51791 (2.375%)	26	15	0	12

		Lewis Structure			
Lewis	non-Lewis	CR	BD	nC	LP
97.06663	2.93337	20	22	0	8
(97.067% of 100)	(2.933% of 100)				

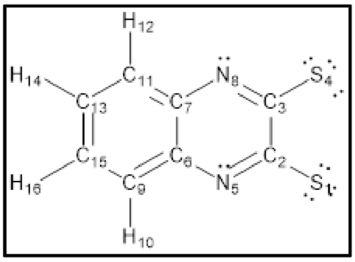

The NBO analysis of the optimised thioquinoxaline reveals 8 lone-pairs, distributed as anticipated in the Lewis structure: 3 lone-pairs to each S atom and 1 LP to each N atom. Interestingly, the NBO analysis indicates a non-Lewis index of 2.933%, surpassing that of thiocatechol.

LONE 1 3 4 3 5 1 8 1 END

BOND S 1 2 S 2 3 D 2 5 S 3 4 D 3 8 S 5 6 S 6 7 D 6 9 S 7 8 D 7 11 S 9 10 S 9 15 S 11 12 S 11 13 S 13 14 D 13 15 S 15 16 END

In Table S10 the orbitals once again show lower occupancies on the third lone-pairs (1.98656 vs 1.62025). No other relevant changes in occupancies are found in either lone-pairs or bonding orbitals.

Visualisation of the orbitals in Figure S35 also shows a proper overlapping between the S1 LP (and S4 LP) with the antibonding of the adjacent C3-N8 bond (and C2-N5).

Table S10. Occupancy on the third lone-pairs and their visualisation.

Lewis		Occupancy
S1	LP	1.98656
	LP	1.92849
	LP	1.62025
S4	LP	1.98656
	LP	1.92849
	LP	1.62025
N5	LP	1.90536
N8	LP	1.90536
double bonds		
(1) C2-N5	BD	1.98715
(2) C2-N5	BD	1.77637
(1) C3-N8	BD	1.98715
(2) C3-N8	BD	1.77637
(1) C6-C9	BD	1.97880
(2) C6-C9	BD	1.60576
(1) C7-C11	BD	1.97880
(2) C7-C11	BD	1.60576
(1) C13-C15	BD	1.98232
(2) C13-C15	BD	1.72566
Non-Lewis		
(1) C2-C3	BD*	0.12549
(2) C2-N5	BD*	0.42470
(2) C3-N8	BD*	0.42470

23. LP (3) S1

54. BD*(2) C2-N5

23. LP (3) S1
+
54. BD*(2) C2-N5

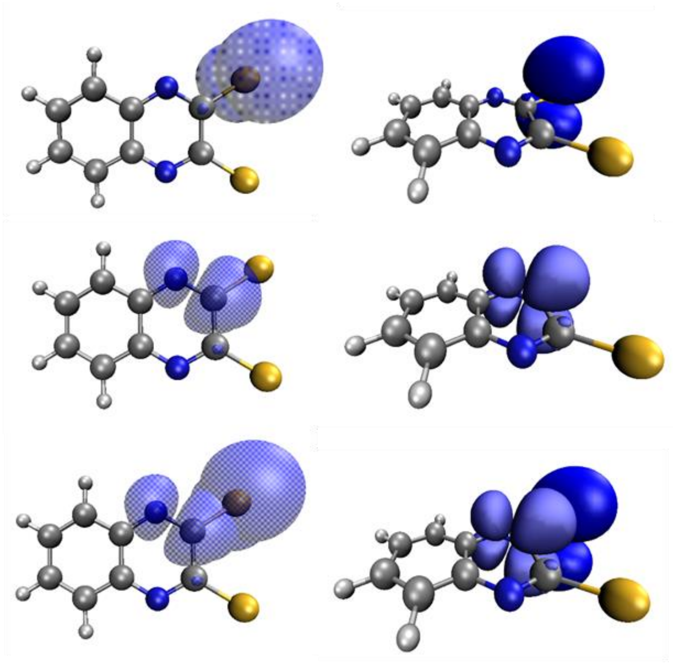

(frontal view) (bottom view)

Figure S35. Visualisation of the main orbitals involved in Table S10.

The 2nd Order Perturbation Analysis shows much higher donation-acceptor transference with higher energies (Table S11). The most substantial transfer once again originates from the lone pair of sulphur to the antibonding of the adjacent double bond (now C-N bond). Moreover, the addition of the second aromatic ring leads to new overlaps between molecular orbitals, basically as a consequence of the expansion of the conjugated system.

Table S11. Report of the NBO's 2nd order perturbation analysis most significative density migrations (higher E(2) value) observed for the thioquinoxaline free ligand. Below, an schematic representation of the conjugation from the sulphide lone pairs described in the table..

Donor (L)	Acceptor (NL)	E(2)	Donor (L)	Acceptor (NL)	E(2)
23. LP (3) S1	54. BD*(2) C2-N5	51.87	32. BD (2) C2-N5	61. BD*(2) C6-C9	32.19
26. LP (3) S4	57. BD*(2) C3-N8	51.87	35. BD (2) C3-N8	64. BD*(2) C7-C 11	32.19
27. LP (1) N5	52. BD*(1) C2-C3	12.96	39. BD (2) C6-C9	71. BD*(2) C 13-C 15	27.50
28. LP (1) N8	52. BD*(1) C2-C3	12.96	39. BD (2) C6-C9	64. BD*(2) C7-C 11	18.11
27. LP (1) N5	59. BD*(1) C6-C7	12.56	42. BD (2) C7-C 11	71. BD*(2) C 13-C 15	27.50
28. LP (1) N8	59. BD*(1) C6-C7	12.56	49. BD (2) C 13-C 15	61. BD*(2) C6-C9	17.62

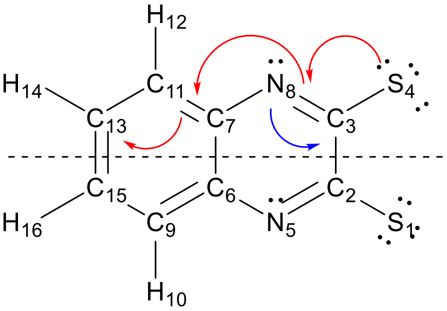

In the case of thioquinoxaline, the Wiberg bond index (WBI) for the C-S bonds is 1.395, which is higher than that of C-S bonds in thiocatechol (see Figure S36), with the bond length also being slightly shorter. Additionally, the WBI for the C-C bond connecting both sulphur atoms is even lower, corresponding to a single bond order (1.537 Å). This observation can be explained by the donation from both nitrogen lone pairs to the C2-C3 bond, a feature not present in the thiocatechol ligand.

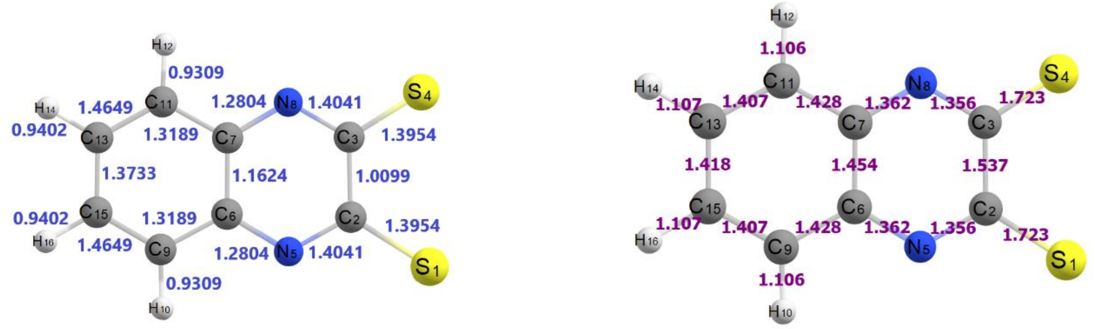

Figure S36. Wiberg bond indexes (left) and distances in Å (right) for thioquinoxaline.

Table S12. Summary of the NBO Analysis for the two proposed resonance structures for thioquinoxaline A and B and their schematic representation. The Lewis character indicate the degree of fit in the imposed constraints of each isomer.

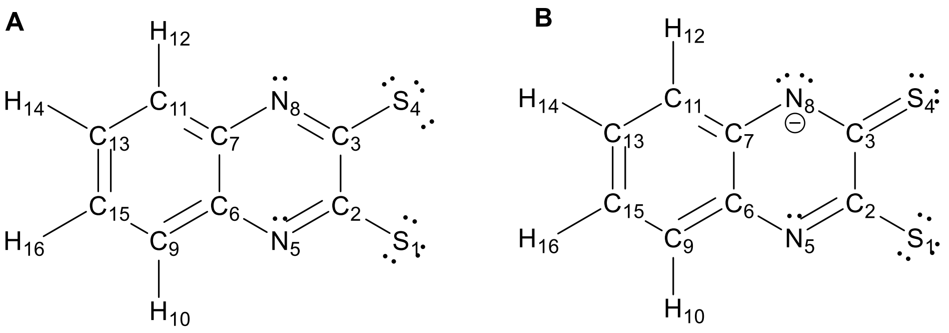

			Lewis Structure			
	Lewis	non-Lewis	CR	BD	nC	LP
A	**97.06663**	**2.93337**	20	22	0	8
B	96.92031	3.07969	20	22	0	8

Mulliken charges on sulphur (anionic species):

dithiocatecholate ⟶ −0.633258 & −0.633258

dichlorodithiocatecholate ⟶ −0.549249 & −0.549249

thioquinoxaline ⟶ −0.504840 & −0.504840

A decrease on the sulphur charges is observed when going from dithiocatecholate, dichlorodithiocatecholate and thioquinoxaline ligands. This can be attributed to the delocalisation of the charge onto the additional aromatic ring of thioquinoxaline which has been discussed in the previous section with the tautomerisation studies.

FRONTIER MOLECULAR ORBITALS OF THE LIGANDS

Transitioning from **Ru2** to **Ru3**, there is an increase in the HOMO-LUMO gap (see Table S13), resulting in a notable enhancement in chemical hardness (η). This is mainly attributed to the over-stabilization of the HOMO, which is associated with resonance.

Table S13. Frontier molecular orbitals of the ligands.

NBOs E(eV)	**Ru2**	**Ru3**
**HOMO**	2,997	0,248
**LUMO**	7,863	6,865
η	**2,433**	**3,308**

Chemical hardness (η) --> $\eta = \frac{1}{2}(E_{LUMO} - E_{HOMO})$

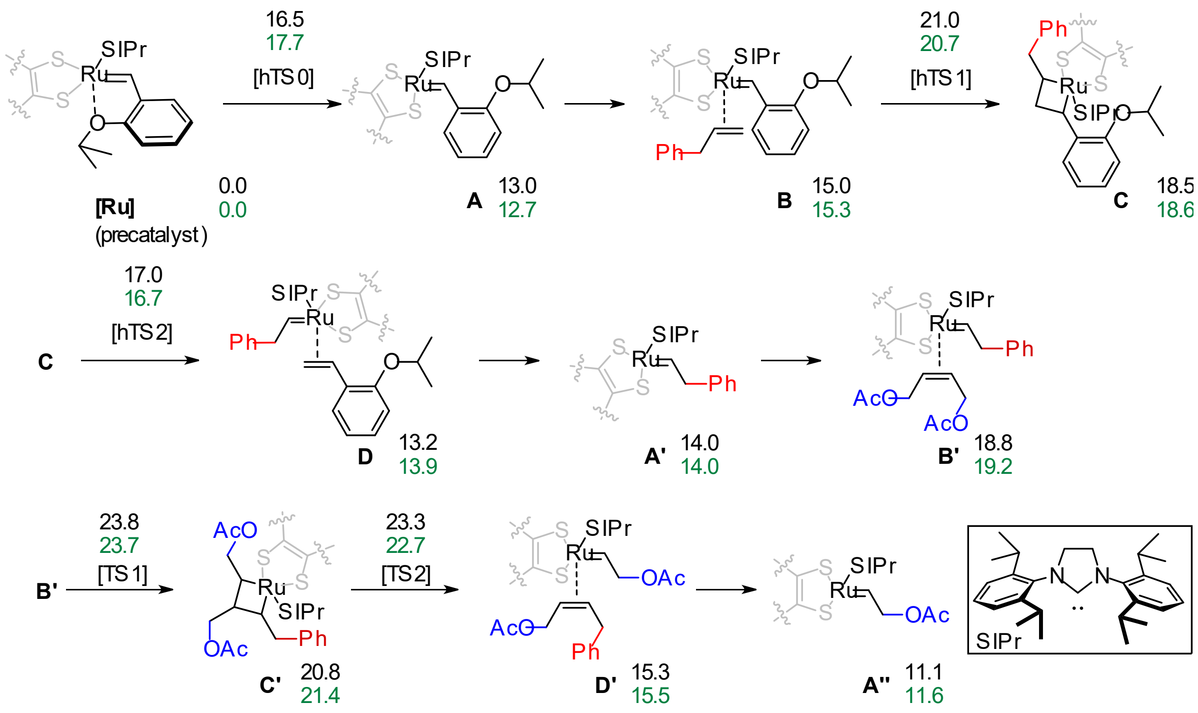

Figure S37. Relative Gibbs energies for the cross metathesis (in kcal/mol).

Table S14. Absolute energies (in a.u.) and relative energies (in kcal/mol) for Ru2 and Ru3.

	**BP86**	**BP86**	**relative**		**M06L**	**M06L**	**relative**	
**Ru2**	Egas	Ggas	Egas	Ggas	Esol	Gsol	Esol	Gsol
**Precat**	−3665,007523	−3664,260776	0,00	0,00	−3666,898158	−3666,151411	0,00	0,00
**hTS0**	−3664,978095	−3664,237097	18,47	14,86	−3666,866167	−3666,125169	20,07	16,47
**A**	−3664,980069	−3664,241374	17,23	12,17	−3666,869441	−3666,130746	18,02	12,97
**B**	−4013,733478	−4012,840169	−9,90	5,09	−4015,920103	−4015,026794	0,06	15,0

hTS1	−4013,72206	−4012,828637	−2,73	12,32	−4015,91072	−4015,017297	5,95	21,01
C	−4013,724587	−4012,831486	−4,32	10,54	−4015,914344	−4015,021243	3,67	18,53
hTS2	−4013,726608	−4012,833586	−5,59	9,22	−4015,916733	−4015,023711	2,17	17
D	−4013,731661	−4012,838633	−8,76	6,05	−4015,922728	−4015,0297	−1,59	13,22
A'	−3511,231479	−3510,54637	21,92	17,39	−3512,98677	−3512,30166	18,51	13,98
B'	−4123,834095	−4122,971435	−10,14	5,73	−4126,146581	−4125,283921	2,90	18,77
TS1	−4123,823696	−4122,961896	−3,62	11,72	−4126,137727	−4125,275927	8,45	23,79
C'	−4123,828418	−4122,965003	−6,58	9,77	−4126,144045	−4125,280631	4,49	20,84
TS2	−4123,829194	−4122,964889	−7,07	9,84	−4126,141065	−4125,27676	6,36	23,27
D'	−4123,841848	−4122,978697	−15,01	1,17	−4126,15266	−4125,289509	−0,92	15,27
A''	−3508,06146	−3507,415314	22,35	15,99	−3509,836036	−3509,18989	17,42	11,05

	BP86	BP86	relative		M06L	M06L	relative	
Ru3	Egas	Ggas	Egas	Ggas	Esol	Gsol	Esol	Gsol
Precat	−2931,601428	−2930,812558	**0,00**	**0,00**	−2933,451544	−2932,662674	**0,00**	**0,00**
hTS0	−2931,570715	−2930,788601	**19,27**	**15,03**	−2933,418228	−2932,636113	**20,91**	**16,67**
A	−2931,573342	−2930,793740	**17,62**	**11,81**	−2933,422037	−2932,642436	**18,52**	**12,70**
B	−3280,325621	−3279,391725	**−8,79**	**5,23**	−3282,471602	−3281,537706	**1,24**	**15,27**
hTS1	−3280,314307	−3279,380621	**−1,69**	**12,20**	−3282,462793	−3281,529107	**6,77**	**20,66**
C	−3280,316797	−3279,382703	**−3,26**	**10,89**	−3282,466440	−3281,532346	**4,48**	**18,63**
hTS2	−3280,319090	−3279,384635	**−4,70**	**9,68**	−3282,469878	−3281,535424	**2,33**	**16,70**
D	−3280,328120	−3279,391277	**−10,36**	**5,51**	−3282,476788	−3281,539945	**−2,01**	**13,86**
A'	−2777,825256	−2777,098779	**22,00**	**17,00**	−2779,539394	−2778,812916	**18,99**	**13,99**
B'	−3390,426132	−3389,522319	**−8,97**	**6,29**	−3392,698352	−3391,794539	**3,91**	**19,18**
TS1	−3390,415718	−3389,513224	**−2,44**	**12,00**	−3392,689760	−3391,787266	**9,30**	**23,74**
C'	−3390,420431	−3389,515825	**−5,39**	**10,37**	−3392,695574	−3391,790968	**5,66**	**21,42**
TS2	−3390,421721	−3389,517104	**−6,20**	**9,57**	−3392,693530	−3391,788913	**6,94**	**22,71**
D'	−3390,43404	−3389,529569	**−13,93**	**1,75**	−3392,704807	−3391,800336	**−0,14**	**15,54**
A''	−2774,654675	−2773,967035	**22,78**	**16,02**	−2776,387928	−2775,700289	**18,35**	**11,59**

Table S15. Evaluation of the 1,2-shift side-reaction depending on the alkene substituents with their corresponding relative free-energies (in kcal/mol, only reagents shown explicitly on the left side of the arrow).

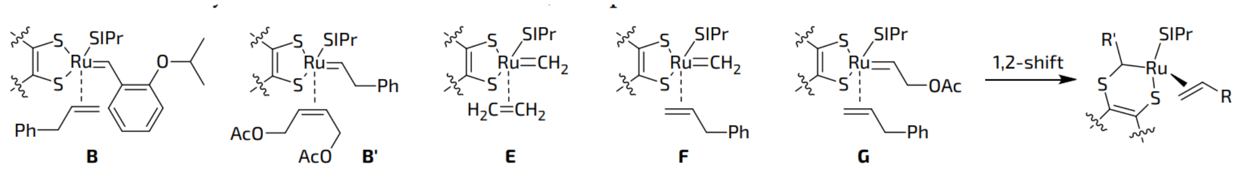

	B		B’		E		F		G	
	Ru2	Ru3	Ru2	Ru3	Ru2	Ru3	Ru2	Ru3	Ru2	Ru3
**R**	15.0	15.3	18.8	19.2	15.5	13.3	11.9	10.2	14.1	14.2
$TS_{1,2\text{-shift}}$	**29.7**	**29.9**	**31.7**	**31.2**	**26.4**	**23.9**	**14.4**	**18.9**	**26.5**	**27.0**
**P**	20.1	20.6	17.3	20.3	12.3	11.9	1.9	2.7	19.7	20.1

3. Supplementary References

1. Szczepaniak, G., Ruszczyńska, A., Kosiński, K., Bulska, E. & Grela, K. Highly efficient and time economical purification of olefin metathesis products from metal residues using an isocyanide scavenger. *Green Chem.* **20**, 1280–1289 (2018).
2. Szczepaniak, G. *et al.* Semiheterogeneous Purification Protocol for the Removal of Ruthenium Impurities from Olefin Metathesis Reaction Products Using an Isocyanide Scavenger. *Org. Process Res. Dev.* **23**, 836–844 (2019).
3. Szczepaniak, G. *et al.* An isocyanide ligand for the rapid quenching and efficient removal of copper residues after Cu/TEMPO-catalyzed aerobic alcohol oxidation and atom transfer radical polymerization. *Chem. Sci.* **11**, 4251–4262 (2020).
4. Grudzień, K., Nogaś, W., Szczepaniak, G. & Grela, K. Larger scale Stahl oxidation with instant Cu removal in convenient synthesis of chiral bidentate N–heterocyclic carbene precursor. *Polyhedron* **199**, 115090 (2021).
5. Szczepaniak, G. *et al.* High-Performance Isocyanide Scavengers for Use in Low-Waste Purification of Olefin Metathesis Products. *ChemSusChem* **8**, 4139–4148 (2015).
6. Koh, M. J. *et al.* High-value alcohols and higher-oxidation-state compounds by catalytic Z-selective cross-metathesis. *Nature* **517**, 181–186 (2015).
7. Mikus, M. S., Torker, S. & Hoveyda, A. H. Controllable ROMP Tacticity by Harnessing the Fluxionality of Stereogenic-at-Ruthenium Complexes. *Angew. Chem. Int. Ed.* **55**, 4997–5002 (2016).
8. García-Álvarez, I. *et al.* Synthesis of Antimitotic Thioglycosides: In Vitro and in Vivo Evaluation of Their Anticancer Activity. *J. Med. Chem.* **54**, 6949–6955 (2011).
9. da Silva Miranda, F. *et al.* Synthesis of substituted dipyrido[3,2-*a*:2′,3′-*c*]phenazines and a new heterocyclic dipyrido[3,2-*f*:2′,3′-*h*]quinoxalino[2,3-*b*]quinoxaline. *Tetrahedron* **64**, 5410–5415 (2008).

10. Fathalla, W., Ali, I. A. I. & Pazdera, P. A novel method for heterocyclic amide–thioamide transformations. *Beilstein J. Org. Chem.* **13**, 174–181 (2017).

11. Sytniczuk, A. *et al.* At Long Last: Olefin Metathesis Macrocyclization at High Concentration. *J. Am. Chem. Soc.* **140**, 8895–8901 (2018).

12. Sytniczuk, A., Milewski, M., Dąbrowski, M., Grela, K. & Kajetanowicz, A. Schrock molybdenum alkylidene catalyst enables selective formation of macrocyclic unsaturated lactones by ring-closing metathesis at high-concentration. *Green Chem.* **25**, 2299–2304 (2023).

13. Grzesiński, Ł., Milewski, M., Nadirova, M., Kajetanowicz, A. & Grela, K. Unexpected Latency of *Z*-Stereoretentive Ruthenium Olefin Metathesis Catalysts Bearing Unsymmetrical N-heterocyclic Carbene or Cyclic(alkyl)(amino)carbene Ligands. *Organometallics* **42**, 2453–2459 (2023).

14. Morvan, J. *et al.* Continuous Flow Z-Stereoselective Olefin Metathesis: Development and Applications in the Synthesis of Pheromones and Macrocyclic Odorant Molecules**. *Angew. Chem. Int. Ed.* **60**, 19685–19690 (2021).

15. Rosenblatt, W., Osipow, L. I. & Snell, F. D. Chemical intermediates and derivatives from oleyl alcohol. *J. Am. Oil Chem. Soc.* **43**, 245–248 (1966).

16. Xia, L. *et al.* Grubbs Catalysts Immobilized on Merrifield Resin for Metathesis of Leaf Alcohols by using a Convenient Recycling Approach. *ChemistryOpen* **8**, 45–48 (2019).

17. Obando, D. *et al.* Synthesis and Evaluation of a Series of Bis(pentylpyridinium) Compounds as Antifungal Agents. *ChemMedChem* **13**, 1421–1436 (2018).

18. Al-Shuhaib, Z. *et al.* Intramolecular palladium mediated π-allyl cyclisation of bis-Cbz- and bis-Boc-protected guanidines. *Tetrahedron Lett.* **54**, 6716–6718 (2013).

19. Dowden, J. & Savović, J. Olefin metathesis in non-degassed solvent using a recyclable, polymer supported alkylideneruthenium. *Chem. Commun.* 37–38 (2001) doi:10.1039/B007304K.

20. Gawin, R., Czarnecka, P. & Grela, K. Ruthenium catalysts bearing chelating carboxylate ligands: application to metathesis reactions in water. *Tetrahedron* **66**, 1051–1056 (2010).

21. Liu, M.-S., Du, H.-W. & Shu, W. Metal-free allylic C–H nitrogenation, oxygenation, and carbonation of alkenes by thianthrenation. *Chem. Sci.* **13**, 1003–1008 (2022).

22. Vinczer, P., Novák, L. & Szántay, C. APPLICATION OF POTASSIUM t-BUTOXIDE IN TOLUENE AS A BASE IN THE WITTIG REACTION IN LARGE-SCALE PHEROMONE SYNTHESES. *Org. Prep. Proced. Int.* **23**, 443–447 (1991).

23. Zheng, Y. *et al.* *trans*-2-Aminocyclohexanol-based amphiphiles as highly efficient helper lipids for gene delivery by lipoplexes. *Biochim. Biophys. Acta BBA - Biomembr.* **1848**, 3113–3125 (2015).

24. Herbert, M. B., Marx, V. M., Pederson, R. L. & Grubbs, R. H. Concise Syntheses of Insect Pheromones Using Z-Selective Cross Metathesis. *Angew. Chem. Int. Ed.* **52**, 310–314 (2013).

25. Wang, G.-Z. *et al.* Copper-Catalyzed Cross-Coupling Reaction of Allyl Boron Ester with 1°/2°/3°-Halogenated Alkanes. *Org. Lett.* **17**, 3682–3685 (2015).

26. Zhu, Q., Luo, Y., Guo, Y., Zhang, Y. & Tao, Y. Saegusa Oxidation of Enol Ethers at Extremely Low Pd-Catalyst Loadings under Ligand-free and Aqueous Conditions: Insight into the Pd(II)/Cu(II)-Catalyst System. *J. Org. Chem.* **86**, 5463–5476 (2021).

27. Gaussian 16, Revision C.01, M. J. Frisch, G. W. Trucks, H. B. Schlegel, G. E. Scuseria, M. A. Robb, J. R. Cheeseman, G. Scalmani, V. Barone, G. A. Petersson, H. Nakatsuji, X. Li, M. Caricato, A. V. Marenich, J. Bloino, B. G. Janesko, R. Gomperts, B. Mennucci, H. P. Hratchian, J. V. Ortiz, A. F. Izmaylov, J. L. Sonnenberg, D. Williams-Young, F. Ding, F. Lipparini, F. Egidi, J. Goings, B. Peng, A. Petrone, T. Henderson, D. Ranasinghe, V. G. Zakrzewski, J. Gao, N. Rega, G. Zheng, W. Liang, M. Hada, M. Ehara, K. Toyota, R. Fukuda, J. Hasegawa, M. Ishida, T. Nakajima, Y. Honda, O. Kitao, H. Nakai, T. Vreven, K. Throssell, J. A. Montgomery, Jr., J. E. Peralta, F. Ogliaro, M. J. Bearpark, J. J. Heyd, E. N. Brothers, K. N. Kudin, V. N. Staroverov, T. A. Keith, R. Kobayashi, J. Normand, K. Raghavachari, A. P. Rendell, J. C. Burant, S. S. Iyengar, J. Tomasi, M. Cossi, J. M. Millam, M. Klene, C. Adamo, R. Cammi, J. W. Ochterski, R. L. Martin, K. Morokuma, O. Farkas, J. B. Foresman, and D. J. Fox, Gaussian, Inc., Wallingford CT, 2016.

28. Becke, A. D. Density-functional exchange-energy approximation with correct asymptotic behavior. *Phys. Rev. A* **38**, 3098–3100 (1988).

29. Perdew, J. P. Density-functional approximation for the correlation energy of the inhomogeneous electron gas. *Phys. Rev. B* **33**, 8822–8824 (1986).

30. Grimme, S., Antony, J., Ehrlich, S. & Krieg, H. A consistent and accurate ab initio parametrization of density functional dispersion correction (DFT-D) for the 94 elements H-Pu. *J. Chem. Phys.* **132**, 154104 (2010).

31. Grimme, S. Semiempirical hybrid density functional with perturbative second-order correlation. *J. Chem. Phys.* **124**, 034108 (2006).

32. Weigend, F. & Ahlrichs, R. Balanced basis sets of split valence, triple zeta valence and quadruple zeta valence quality for H to Rn: Design and assessment of accuracy. *Phys. Chem. Chem. Phys.* **7**, 3297–3305 (2005).

33. Häussermann, U. *et al.* Accuracy of energy-adjusted quasirelativistic ab initio pseudopotentials: All-electron and pseudopotential benchmark calculations for Hg, HgH and their cations. *Mol. Phys.* **78**, 1211–1224 (1993).

34. Küchle, W., Dolg, M., Stoll, H. & Preuss, H. Energy-adjusted pseudopotentials for the actinides. Parameter sets and test calculations for thorium and thorium monoxide. *J. Chem. Phys.* **100**, 7535–7542 (1994).

35. Leininger, T., Nicklass, A., Stoll, H., Dolg, M. & Schwerdtfeger, P. The accuracy of the pseudopotential approximation. II. A comparison of various core sizes for indium pseudopotentials in calculations for spectroscopic constants of InH, InF, and InCl. *J. Chem. Phys.* **105**, 1052–1059 (1996).

36. Zhao, Y. & Truhlar, D. G. The M06 suite of density functionals for main group thermochemistry, thermochemical kinetics, noncovalent interactions, excited states, and transition elements: two new functionals and systematic testing of four M06-class functionals and 12 other functionals. *Theor. Chem. Acc.* **120**, 215–241 (2008).

37. Schäfer, A., Horn, H. & Ahlrichs, R. Fully optimized contracted Gaussian basis sets for atoms Li to Kr. *J. Chem. Phys.* **97**, 2571–2577 (1992).

38. Barone, V. & Cossi, M. Quantum Calculation of Molecular Energies and Energy Gradients in Solution by a Conductor Solvent Model. *J. Phys. Chem. A* **102**, 1995–2001 (1998).

39. Tomasi, J. & Persico, M. Molecular Interactions in Solution: An Overview of Methods Based on Continuous Distributions of the Solvent. *Chem. Rev.* **94**, 2027–2094 (1994).
